# The scale of the evidence base on the health effects of conventional yogurt consumption: findings of a scoping review

**DOI:** 10.3389/fphar.2015.00246

**Published:** 2015-10-30

**Authors:** Julie M. Glanville, Sam Brown, Raanan Shamir, Hania Szajewska, Jacqualyn F. Eales

**Affiliations:** ^1^York Health Economics Consortium, University of YorkYork, UK; ^2^Sackler Faculty of Medicine, Schneider Children's Medical Center, Institute of Gastroenterology, Nutrition and Liver Diseases, Tel-Aviv UniversityTel-Aviv, Israel; ^3^Department of Paediatrics, The Medical University of WarsawWarsaw, Poland

**Keywords:** yogurt, health outcomes, review, effects, evidence

## Abstract

**Background:** The health effects of conventional yogurt have been investigated for over a century; however, few systematic reviews have been conducted to assess the extent of the health benefits of yogurt.

**Objective:** The aim of this scoping review was to assess the volume of available evidence on the health effects of conventional yogurt.

**Methods:** The review was guided by a protocol agreed *a priori* and informed by an extensive literature search conducted in November 2013. Randomized controlled trials were selected and categorized according to the eligibility criteria established in the protocol.

**Results:** 213 studies were identified as relevant to the scoping question. The number of eligible studies identified for each outcome were: bone health (14 studies), weight management and nutrition related health outcomes (81 studies), metabolic health (6 studies); cardiovascular health (57 studies); gastrointestinal health (24 studies); cancer (39 studies); diabetes (13 studies), Parkinson's disease risk (3 studies), all-cause mortality (3 studies), skin complaints (3 studies), respiratory complaints (3 studies), joint pain/function (2 studies); the remaining 8 studies reported a variety of other outcomes. For studies of a similar design and which assessed the same outcomes in similar population groups, we report the potential for the combining of data across studies in systematic reviews.

**Conclusions:** This scoping review has revealed the extensive evidence base for many outcomes which could be the focus of systematic reviews exploring the health effects of conventional yogurt consumption.

## Introduction

Yogurt consumption has been associated with health and well-being for hundreds of years, but scientific research efforts on the potential health effects of conventional yogurt mainly started during the last century. Conventional yogurt contains a large quantity of nutrients essential for health and has relatively low calorie content, making it a high nutritional density product. In addition, the changes in milk constituents that occur during lactic acid fermentation influence the nutritional and physiological value of yogurt (Hewitt and Bancroft, 1985; Bianchi-Salvadori, 1986; Bourlioux and Pochart, 1988; Adolfsson et al., 2004). The efficacy of yogurt has been investigated in relation to a wide range of separate and overlapping outcomes including weight management (Burns et al., 1998), type 2 diabetes (O'Connor et al., 2014), cardiovascular disease risk (Buyuktuncer et al., 2013), bone health (Heaney et al., 2002), dental health (Telgi et al., 2013), the risk of various forms of cancer (Kurahashi et al., 2008), gastrointestinal (GI) health (Pashapour and Iou, 2006; Ballesta et al., 2008), lactose intolerance (Adibi et al., 2009), malnutrition (Sazawal et al., 2013), immunological parameters (Olivares et al., 2006), and overall mortality (Goldbohm et al., 2011). While a range of research designs have been employed to examine the health effects of yogurt, including observational studies (Cramer et al., 1989; Arslantas et al., 2008; Dawczynski and Jahreis, 2009) and experimental studies (Bonjour et al., 2013; Douglas et al., 2013), systematic reviews (and meta-analyses when appropriate) have been conducted in relation to only a few health outcomes (Tong et al., 2011; Aune et al., 2012, 2013; Soedamah-Muthu et al., 2012; Gao et al., 2013; O'sullivan et al., 2013).

The objective of this scoping review was to assess the volume of evidence for the health effects of yogurt consumption. The scoping review focused on conventional yogurt as defined by the Codex Alimentarius. The Codex Alimentarius Commission was established in the 1960s by the Food and Agriculture Organization of the United Nations (FAO) and the World Health Organization (WHO) and represents an international reference point for food safety and consumer protection (WHO, 2006). The standard for fermented milks (CODEX STAN 243-2003) (Codex Committee on Milk and Milk Products, 2010) defines yogurt as specifically characterized by the presence of the symbiotic starter cultures *Streptococcus thermophilus* and *Lactobacillus delbrueckii* subsp. *bulgaricus* (Codex Committee on Milk and Milk Products, 2010). Furthermore, it states that yogurt obtained through fermentation of milk by cultures of *Streptococcus thermophilus* and any other *Lactobacillus species* should be named through the use of an appropriate qualifier in conjunction with the word yogurt. This has commonly lead to the designation “probiotic yogurt” for fermented milk products containing a different micro-organism with a proven health benefit when taken in adequate amounts (WHO, 2001), and based on the rather widespread opinion that the conventional yogurt starter cultures should not be considered as probiotics.

This scoping review does not concern the tremendous amount of research findings on specific probiotic strains that has been generated during the past 20 years but concentrates on identifying the available evidence base on the generic health effects of conventional yogurt; an analysis undertaken as the first stage to inform potential future systematic reviews.

## Methods

This scoping review was conducted using relevant methods of the systematic review process up to the point of data extraction. It is reported in accordance with the PRISMA reporting guidelines statement and checklist (Liberati et al., 2009) up to that point. The scoping review does not include the detailed data extraction, quality assessment and synthesis of a full systematic review, but is conducted with the aim of being objective, extensive and transparent. The scoping review was guided by a protocol (available for download from www.yhec.co.uk), which was agreed before the searches were conducted.

Studies considered eligible for the scoping review were epidemiological studies, cohort studies, open label studies and randomized controlled trials (RCTs). Case reports, letters, comments, and editorials were not eligible for inclusion. Eligible studies had to fulfill the requirement of examining the effect of oral consumption of conventional yogurt in the general population, in relation to a range of health outcomes, with a few specific exclusions. Studies that only examined the health effects of yogurt in the following situations were excluded: human populations with specific diseases; any animal population; *in vitro* studies and studies using technologies that simulate the stomach environment. We also excluded studies if they only assessed the yogurt interventions in relation to any of the following: fecal count outcomes; outcomes relating to stomach flora; overall assessments of diets where yogurt is only one factor and not reported separately; dental health, lactose intolerance; contagious diseases; treatment of infectious GI/respiratory tract diseases; studies reporting laboratory or immunological parameters only; inflammatory diseases; autoimmune diseases; eye diseases (e.g., age-related macular degeneration) and cataracts; vaginitis; or studies of yogurt interference with antibiotic uptake.

Studies investigating conventional yogurt as either a single intervention or in combination with any other non-probiotic substance were eligible for inclusion. Studies that compared conventional yogurt with any non-probiotic yogurt, any non-yogurt substance, or placebo were also eligible. Studies of yogurt supplemented with probiotics, fermented milk products, such as kefir and kumys, fermented baby formula, or milk were not eligible for the scoping review.

### Data sources and search strategy

An extensive literature search was conducted during November 2013 in a range of relevant databases to identify studies investigating the health effects of yogurt. The search was limited to conventional yogurt as defined by the Codex Alimentarius international food standards for fermented milks (CODEX STAN 243-2003) (Codex Committee on Milk and Milk Products, 2010).

**Figure 1:**
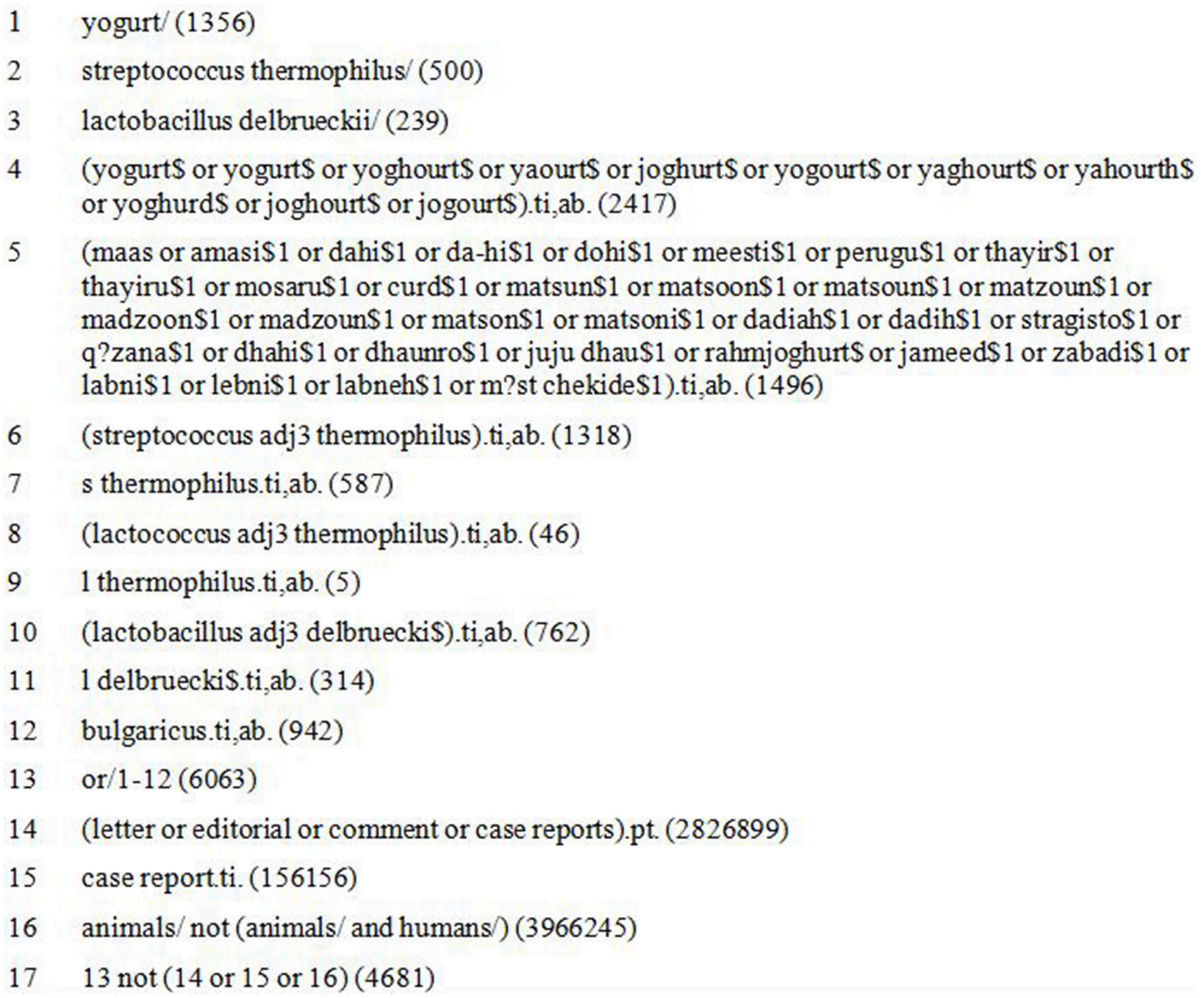
**Medline search strategies to identify studies reporting the health benefits of yogurt**.

The full list of databases searched is included in Supplementary Table B. The searches were not limited by date or language. Information on ongoing or recently completed studies, unpublished research, and research reported in the gray literature was identified by searching selected major relevant conference proceedings from the past 3 years. Gray literature was identified via OAISTER, OpenGray and NTIS. The search strategy involved only search terms for conventional yogurts, including many synonyms used in different parts of the world for this traditional foodstuff, as shown in the Medline search strategy (Figure 1).

Reference lists of relevant reviews, trials and studies were used to identify any additional studies that might be eligible for inclusion.

### Study selection

Record selection was undertaken using several passes. The first pass was undertaken by one reviewer (SB) in order to rapidly remove obviously irrelevant records such as research undertaken in animals or case reports. Second pass record selection was undertaken by two reviewers (JE, SB) independently, using the title and abstract of records. The full text of included studies was then assessed for relevance by one of the authors (JE) and checked by a second independent reviewer (SB). Discrepancies were resolved through discussion and where necessary by consulting a third reviewer.

### Data extraction

One researcher (JE) extracted selected data from the full papers of each of the included studies using a standardized template into an Excel spreadsheet, and a second researcher (SB) checked the extraction. In the absence of full paper copies or when only abstracts were available, data were extracted from the abstracts alone. Details of the information extracted from the included studies are listed in Supplementary Table C.

**Figure 2:**
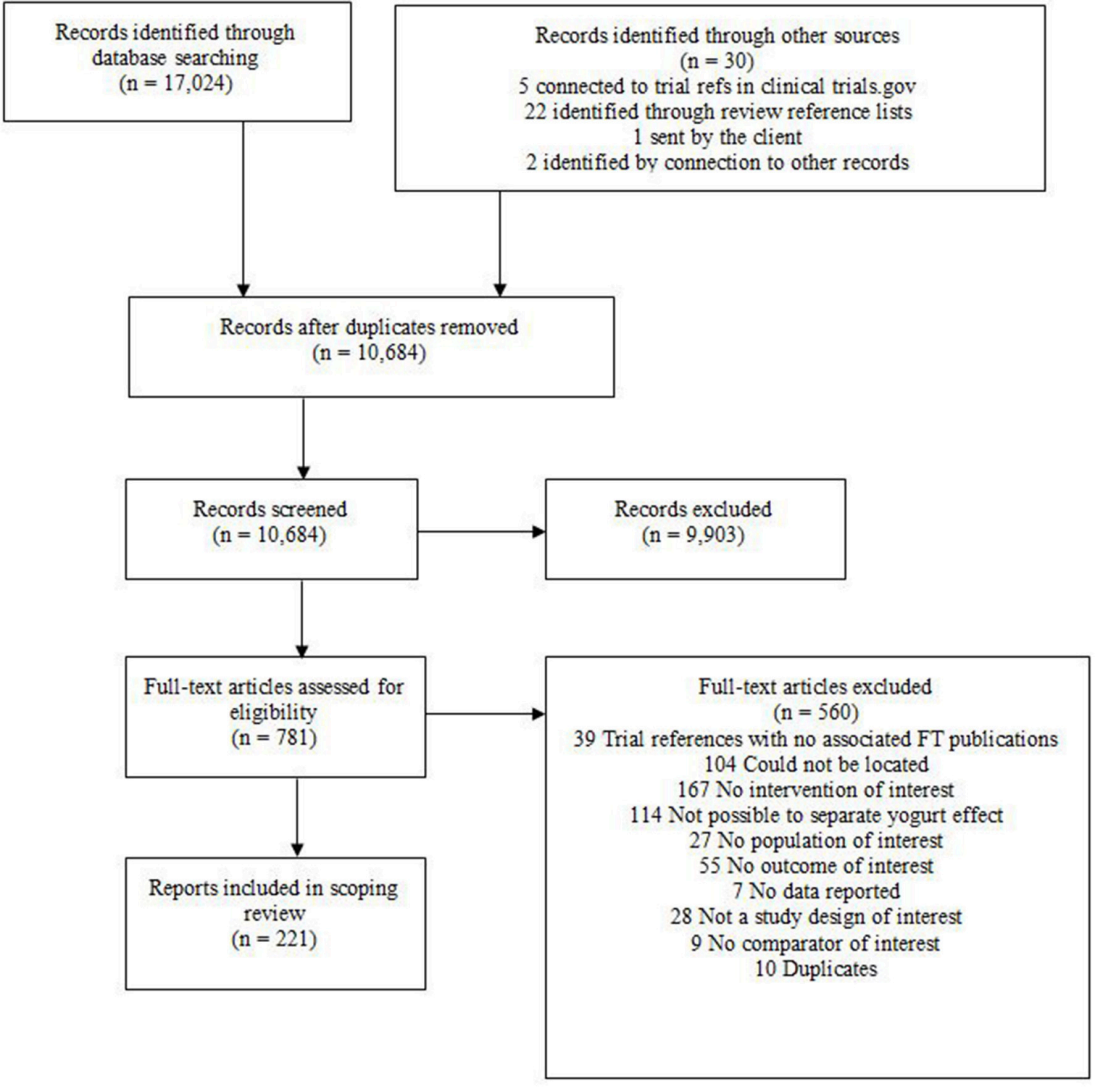
**PRISMA flowchart**.

At the data extraction stage some studies, on closer inspection, proved ineligible. The number of records lost at this stage of the review process is documented in the PRISMA flow diagram (Figure 2).

## Results

A total of 17,024 records were identified by the searches and an additional 30 records through other sources. After de-duplication 10,684 records were taken forward for title and abstract screening. A further 9,903 records were excluded by the second pass, leaving 781 records for full text assessment. Of these 560 were excluded in the third pass leaving a total of 221 eligible studies. The number of studies identified by the searches at the various selection stages is reported in a PRISMA study flow diagram (Figure 2). Of these, 213 studies were available with either an English abstract or English full text and are included in the categorization. Studies available only as abstracts were included in this scoping review, because there was generally sufficient information to suggest potential eligibility.

We grouped study designs into broad categories for the purpose of this report, to provide information on the type of study, rather than the internal quality of the study. Generally, the RCTs, cohort studies (not population-based cohort studies) and cross-over trials had small study sizes, with 100 participants or fewer entering the studies. Cross-sectional studies and population-based (or large hospital-based) cohort studies had the largest study numbers, ranging from hundreds to tens of thousands of participants being enrolled. Case-control and case-cohort studies generally involved numbers of participants in the hundreds or thousands.

### Reported outcomes

The outcomes reported in eligible studies were: bone health (14 studies) (Motegi et al., 2001; Heaney et al., 2002; Berberidis et al., 2004; Sorenson et al., 2004; Arslantas et al., 2008; Jha et al., 2010; Uenishi and Nakamura, 2010; Bener and El Ayoubi, 2012; Nasrollahi et al., 2012; Sahni et al., 2012, 2013a,b; Bonjour et al., 2013; Feart et al., 2013), weight management and nutrition-related health outcomes (81 studies) (Jordan et al., 1981; Thompson et al., 1982; Bazzarre et al., 1983; Massey, 1984; McNamara et al., 1989; Sullivan et al., 1989; Rolls et al., 1991, 1994, 1995; Trapp et al., 1993; Vandewater and Vickers, 1996; Oosthuizen et al., 1998; Campbell et al., 1999, 2000; Burns et al., 2000, 2001, 2002; Hoffman et al., 2000; Zandstra et al., 2000; Mensink et al., 2002; Mossavar-Rahmani et al., 2002; O'Donovan et al., 2003; Rodriguez-Artalejo et al., 2003; Chien et al., 2004; Sorenson et al., 2004; King et al., 2005; Rosado et al., 2005; Yae et al., 2005; Zemel et al., 2005; Logan et al., 2006; Nobre et al., 2006; Tsuchiya et al., 2006; Albertson et al., 2007; Dewan et al., 2007, 2009; Diepvens et al., 2007, 2008; Nazare et al., 2007; Snijder et al., 2007; Beydoun et al., 2008; Bonet Serra et al., 2008; Vergnaud et al., 2008; van der Zander et al., 2008; Almiron-Roig et al., 2009; Berkey et al., 2009; Jordão et al., 2009; White et al., 2009; Chapelot and Payen, 2010; Hursel et al., 2010; Keast et al., 2010, 2013; Lluch et al., 2010; Ortinau et al., 2010, 2012a,b, 2013; Pounis et al., 2010; Blom et al., 2011; Clegg et al., 2011; Jodkowska et al., 2011; Joshi et al., 2011; Margolis et al., 2011; Mozaffarian et al., 2011; Pordeus Luna et al., 2011; Schusdziarra et al., 2011; Smit et al., 2011; Thomas et al., 2011; Bener and El Ayoubi, 2012; Dougkas et al., 2012; Hogenkamp et al., 2012; Salakidou et al., 2012; Azadbakht et al., 2013; Buyuktuncer et al., 2013; Douglas et al., 2013; Meneton et al., 2013; Mensah and Otoo, 2013; Sazawal et al., 2013; Stritecka and Hlubik, 2013; Wang et al., 2013; Dawczynski et al., 2013; O'Connor et al., 2014), metabolic health (6 studies) (Snijder et al., 2007; Beydoun et al., 2008; Bonet Serra et al., 2008; White et al., 2009; Troy et al., 2010; Kim, 2013), cardiovascular health (57 studies) (Hepner et al., 1979; Rossouw et al., 1981; Thompson et al., 1982; Bazzarre et al., 1983; Jaspers et al., 1984; Massey, 1984; Cramer et al., 1989; McNamara et al., 1989; Sullivan et al., 1989; Freudenheim et al., 1991; Trapp et al., 1993; Oosthuizen et al., 1998; Iso et al., 1999; Mensink et al., 2002; Nakamura et al., 2002; Tavani et al., 2002; Steffen and Jacobs, 2003; Chien et al., 2004; Sorenson et al., 2004; Trautwein et al., 2004, 2005; Ganji and Kafai, 2004a,b; Steffen et al., 2005; Yae et al., 2005; Korpela et al., 2006; Rudkowska et al., 2007, 2008; Snijder et al., 2007; Bonet Serra et al., 2008; Masala et al., 2008; Niittynen et al., 2008; van der Zander et al., 2008; Wang et al., 2008, 2012, 2013; Dawczynski and Jahreis, 2009; Khandelwal et al., 2009; Larsson et al., 2009; Bonthuis et al., 2010; Sadrzadeh-Yeganeh et al., 2010; Clegg et al., 2011; Goldbohm et al., 2011; Ivey et al., 2011; Radler et al., 2011; Recio et al., 2011; Zhang et al., 2011; Amir Shaghaghi, 2012; Gouni-Berthold et al., 2012; Soedamah-Muthu et al., 2012; Azadbakht et al., 2013; Buyuktuncer et al., 2013; Javed et al., 2013; Kim, 2013; Meneton et al., 2013; Dawczynski et al., 2013), GI health (24 studies) (Niv et al., 1963; Dehesa et al., 1986; Porkka et al., 1988; Boudraa et al., 1989, 1990, 2001; Karabocuoglu et al., 1993; Trapp et al., 1993; Bhatnagar et al., 1998; Teuri and Korpela, 1998; Nakamura et al., 2000, 2001; Vazquez Martinez et al., 2005; Pashapour and Iou, 2006; Conway et al., 2007; Ranasinghe et al., 2007; Ballesta et al., 2008; Rafeey et al., 2008; Haenni et al., 2009; Pilipenko et al., 2009; Eren et al., 2010; Clegg et al., 2011; Frank et al., 2012; Isakov et al., 2013), cancer (39 studies) (Cook-Mozaffari et al., 1979; Le et al., 1986; Cramer et al., 1989; van't Veer et al., 1989; Peters et al., 1992; Kampman et al., 1994, 2000; Boutron et al., 1996; Shannon et al., 1996; Kocic et al., 1997; Ronco et al., 2002; Radosavljevic et al., 2003; Vlajinac et al., 2003; Juarranz Sanz et al., 2004; Kojima et al., 2004; Sakauchi et al., 2004; Sorenson et al., 2004; Kesse et al., 2005, 2006; Lin et al., 2005; Gallus et al., 2006; Genkinger et al., 2006; Mommers et al., 2006; Hsu et al., 2007; Janoutova et al., 2007; Matsumoto et al., 2007; Ornelas et al., 2007; Park et al., 2007; Heck et al., 2008; Kurahashi et al., 2008; Bonthuis et al., 2010; Karagianni et al., 2010; Djonovic and Arsenijevic, 2011; Pala et al., 2011; Faber et al., 2012; Kawakita et al., 2012; Reyhani et al., 2012; Duarte-Salles et al., 2013; Murphy et al., 2013), and diabetes (13 studies) (Nakamura et al., 2002; Sorenson et al., 2004; Choi et al., 2005; Liu et al., 2006; Kirii et al., 2009; Margolis et al., 2011; Dougkas et al., 2012; Sluijs et al., 2012; Soedamah-Muthu et al., 2012; Gheller et al., 2013; Grantham et al., 2013; Wang et al., 2013; O'Connor et al., 2014). Supplementary Tables D–J provide details of the studies identified in this scoping review for the outcomes of interest, presented in separate tables for each outcomecategory.

Twenty-two studies assessed other outcomes: Parkinson's disease risk (3 studies) (Chen et al., 2007; Miyake et al., 2011; Kyrozis et al., 2013), all-cause mortality (3 studies) (Bonthuis et al., 2010; Goldbohm et al., 2011; Soedamah-Muthu et al., 2012), skin complaints (3 studies) (Uenishi et al., 2004, 2008; Kim et al., 2010), respiratory complaints (3 studies) (Miyake et al., 2010, 2012; Maslova et al., 2012), joint pain/function (2 studies) (Martinez-Puig et al., 2013; Morina et al., 2013). The remaining 8 studies assessed a variety of other health outcomes: benign breast disease risk (Berkey et al., 2013), estrogen metabolism (Campbell et al., 1999), general mental/ psychological health (Crichton et al., 2010), minor health complaints (Hyland and Sodergren, 1998), immune function (Makino et al., 2010), general health (Mossavar-Rahmani et al., 2002), age of menarche (Ramezani Tehrani et al., 2013), and allergic symptoms (Trapp et al., 1993). Details of these studies are included in Supplementary Table K.

RCTs made up a large proportion of the weight management and nutritional health (23%), cardiovascular health (26%) and GI health studies (33%). Cross-sectional studies made up a large proportion of the weight management and nutritional health (18%), cardiovascular health (18%), metabolic health (50%) and bone health (50%) studies. Cohort studies made up a large proportion of the cancer (21%), cardiovascular health (26%), GI health (29%), other (41%), and diabetes (46%) studies. Cross-over trials were a common study design for cardiovascular health (19%) and weight management and nutritional (40%) health studies. Case-control and case-cohort studies were generally less common, although case-control studies were the most common study design in the cancer studies (59%) group.

We identified which studies might be similar enough to be suitable for combination in meta-analyses; details are included in Supplementary Table A.

## Discussion

Our scoping review shows that there is a substantial evidence base for investigating the health effects of conventional yogurt and that this evidence base is largest around weight management and nutrition-related health outcomes, cardiovascular health, GI health and cancer.

In principle, a systematic review with a narrative (textual) synthesis can be undertaken for outcomes with no evidence or little evidence, but when there is a larger evidence base there is greater opportunity for more robust assessments of effects. We suggest there are opportunities for meta-analyses among this evidence base where we have found studies that seem to be of similar design, investigating similar interventions and populations, and using the same outcome measures. The study population size is important, due to the inability to achieve significant results with small sample size.

Our results provide a useful evidence base for those interested in developing future nutritional interventions with conventional yogurt. Researchers planning new studies should ideally design them in the light of well-conducted systematic reviews (Al-Shahi Salman et al., 2014). This evidence base provides information which can both inform study design and provide the information for such systematic reviews. Based on this scoping review, three systematic reviews have already been undertaken: two of these included meta-analyses on the role of conventional yogurt in GI health in children (Patro-Golab et al., 2015a,b), and the third is ongoing and examines the effect of conventional yogurt on weight management outcomes.

### Limitations of this scoping review

Studies only available as abstracts (e.g., conference abstracts) were included in this scoping review. Many of these studies would require further information from study authors to confirm their full eligibility to contribute to individual systematic review questions.

Some studies, particularly those by the same author(s) may contain the same, or part of the same, populations. As part of a full systematic review, duplicated or partly duplicated study populations would be identified and not pseudoreplicated. Because a scoping review involves only a high-level overview of study characteristics, we could not identify such duplicated populations with certainty, so there may exist a low degree of double counting in this scoping review.

Due to the limited level of data extraction undertaken in a scoping review, the assessment of the potential for combining studies in a meta-analysis must come with the *caveat* that some studies, especially those for which full study details were not available or reported may not, following assessment of the full study details, be eligible for meta-analysis. We highlight the need to contact authors where there is a lack of clarity about the eligibility of studies with respect to the design, population, intervention, comparator and/or outcomes. This scoping review did not assess the quality of eligible studies. In a full systematic review, the impact of including studies that are categorized with a high risk of bias may be explored in the meta-analyses via subgroup analyses.

The searches were conducted in November 2013. The volume of studies was such that processing took much longer than expected. However, the full strategies are presented in the supplementary files and this should make updating the scoping review relatively straightforward.

## Conclusion

This scoping review identified a number of outcomes for which there exists substantial primary evidence that may be suitable for systematic review and potentially meta-analysis. Future systematic reviews of selected outcomes may provide further evidence for the health effects of yogurt consumption. Our results provide a useful evidence base for those interested in developing future nutritional interventions with conventional yogurt.

## Author contributions

RS developed the idea for the study; JG designed the research. JE, JG, and SB conducted the research; JE and JG analyzed data. SB, JE, and JG co-wrote the report and the paper, with comments and edits from RS and HS. All authors take responsibility for final content. All authors read and approved the final manuscript.

### Conflict of interest statement

YHEC was commissioned to conduct the scoping review with funding provided by Danone Institute International. Danone Institute International had no role in the design, analysis or writing of this article. RS has participated as a clinical investigator, or advisory board member, or consultant or speaker for Abbott, Danone, Enzymotec, Ferrero, Nestle Nutrition Institute, Nutricia and Teva. HS has received funding from Danone Institute International. YHEC (JG, JE, SB) has received funding from Danone Institute International to conduct this scoping review.

## Abbreviations

GI: gastrointestinal
RCT: randomized controlled trial.
