# Supplementary material for: The scale of the evidence base on the health effects of conventional yogurt consumption: findings of a scoping review
Source: Front Pharmacol. 2015 Oct 30;6:246. doi: 10.3389/fphar.2015.00246 (PMC4626633; doi:10.3389/fphar.2015.00246)
Supplement: Supplementary file 1 [file Table1.DOCX]

***Supplementary Material***

**The scale of the evidence base on the health effects of conventional yogurt consumption: findings of a scoping review**

Julie M. Glanville*, Sam Brown, Raanan Shamir, Hania Szajewska, Jacqualyn F. Eales

* **Correspondence:** Julie Glanville, York Health Economics Consortium (YHEC), Enterprise House, University of York, Heslington, YO10 5NQ, UK. Email: julie.glanville@york.ac.uk

**Supplementary Table A: Studies potentially suitable for combination in meta-analyses**

| **Number of studies potentially suitable for combination** | **References** | **Study design** | **Eligible outcomes** | **Age group** |
| --- | --- | --- | --- | --- |
| ***Bone health*** | | | | |
| 3 | Arslantas et al. (2008) ([17](#_ENREF_17)); Sorenson et al. (2004) ([41](#_ENREF_41));  Nasrollahi et al. (2012) ([37](#_ENREF_37)) | Cross-sectional | Osteoporosis | Adults; Older adults |
| 3 | Berberidis et al. (2004) ([33](#_ENREF_33)); Motegi et al. (2001) ([36](#_ENREF_36)); Sahni et al. (2012) ([38](#_ENREF_38)) | Cross-sectional | Bone density | Adults; Older adults |
| 2* | Sahni et al. (2013a) ([39](#_ENREF_39)); Sahni et al. (2013b) ([40](#_ENREF_40)) | Population-based cohort | Bone density | Adults; Older adults |
| ***Weight management*** | | | | |
| 9 | Azadbakht et al. (2013) ([45](#_ENREF_45)); Blom et al. (2011) ([49](#_ENREF_49)); Buyuktuncer et al. (2013) ([7](#_ENREF_7)); Diepvens et al. (2007) ([63](#_ENREF_63));  Mensink et al. (2002) ([82](#_ENREF_82)); Oosthuizen et al. (1998) ([88](#_ENREF_88));  Thomas et al. (2011) ([106](#_ENREF_106)); van der Zander et al. (2008) ([110](#_ENREF_110));  Zemel et al. (2005) ([117](#_ENREF_117)) | RCT | Body weight | Adults |
| 3 | Nazare et al. (2007) ([85](#_ENREF_85)); Diepvens et al. (2007) ([63](#_ENREF_63));  Oosthuizen et al. (1998) ([88](#_ENREF_88)) | RCT | BMI | Adults |
| 4 | Diepvens et al. (2007) ([63](#_ENREF_63)); Thomas et al. (2011) ([106](#_ENREF_106)); White et al. (2009) ([114](#_ENREF_114)); Zemel et al. (2005) ([117](#_ENREF_117)) | RCT | Total fat | Adults |
| 5 | Azadbakht et al. (2013) ([45](#_ENREF_45)); Buyuktuncer et al. (2013) ([7](#_ENREF_7));  Diepvens et al. (2007) ([63](#_ENREF_63)); Thomas et al. (2011) ([106](#_ENREF_106)); Zemel et al. (2005) ([117](#_ENREF_117)) | RCT | Waist circumference | Adults |
| 6 | Azadbakht et al. (2013) ([45](#_ENREF_45)); Campbell et al. (1999) ([54](#_ENREF_54));  Campbell et al. (2000) ([55](#_ENREF_55)); Dawczynski et al. (2013) ([59](#_ENREF_59));  Salakidou et al. (2012) ([100](#_ENREF_100)); Yae et al. (2005) ([115](#_ENREF_115)) | RCT | Nutrient intake | Adults |
| 2 | Azadbakht et al. (2013) ([45](#_ENREF_45)); Blom et al. (2011) ([49](#_ENREF_49)) |  |  |  |
| 2 | Dewan et al. (2007) ([60](#_ENREF_60)); Dewan et al. (2009) ([61](#_ENREF_61)) | RCT | Recovery from malnutrition | Young children |
| 3 | Margolis et al. (2011) ([77](#_ENREF_77)); Mozaffarian et al. (2011) ([84](#_ENREF_84));  Snijder et al. (2007) ([103](#_ENREF_103)) | Population-based cohort | Body weight | Adults |
| 4 | Chien et al. (2004) ([57](#_ENREF_57)); Jordan et al. (1981) ([69](#_ENREF_69));  Thompson et al. (1982) ([107](#_ENREF_107)); Trapp et al. (1993) ([108](#_ENREF_108)) | Cohort | Body weight | Adults; Older adults |
| 8 | Beydoun et al. (2008) ([48](#_ENREF_48)); Joshi et al. (2011) ([71](#_ENREF_71));  Meneton et al. (2013) ([80](#_ENREF_80)); Mossavar-Rahmani et al. (2002) ([83](#_ENREF_83)); Pounis et al. (2010) ([94](#_ENREF_94)); Sorenson et al. (2004) ([41](#_ENREF_41));  Vergnaud et al. (2008) ([112](#_ENREF_112)); Wang et al. (2013) ([113](#_ENREF_113)) | Cross-sectional | BMI/weight/obesity | Adults; Older adults |
| 2 | Keast et al. (2010) ([72](#_ENREF_72)); Rodriguez-Artalejo et al. (2003) ([95](#_ENREF_95)) | Cross-sectional | BMI/weight/obesity | Older children |
| 4 | Albertson et al. (2007) ([43](#_ENREF_43)); Joshi et al. (2011) ([71](#_ENREF_71));  Vergnaud et al. (2008) ([112](#_ENREF_112)); Wang et al. (2013) ([113](#_ENREF_113)) | Cross-sectional | Nutrient intake | Adults; Older adults |
| 3 | Keast et al. (2010) ([72](#_ENREF_72)); Keast et al. (2013) ([73](#_ENREF_73));  Pordeus Luna et al. (2011) ([93](#_ENREF_93)) | Cross-sectional | Nutrient intake | Older children |
| 5 | Albertson et al. (2007) ([43](#_ENREF_43)); Joshi et al. (2011) ([71](#_ENREF_71));  Keast et al. (2010) ([72](#_ENREF_72)); Vergnaud et al. (2008) ([112](#_ENREF_112));  ang et al. (2013) ([113](#_ENREF_113)) | Cross-sectional | Waist circumference | Adults; older adults |
| 2 | Keast et al. (2013) ([73](#_ENREF_73)); Rodriguez-Artalejo et al.(2003) ([95](#_ENREF_95)) | Cross-sectional | Energy intake | Older children |
| 3 | Bazzarre et al. (1983) ([46](#_ENREF_46)); McNamara et al. (1989) ([79](#_ENREF_79));  Sullivan et al. (1989) ([105](#_ENREF_105)) | Crossover | Body weight | Adults |
| 22 | Almiron-Roig et al. (2009) ([44](#_ENREF_44)); Burns et al. (2002) ([53](#_ENREF_53));  Chapelot & Payen (2010) ([56](#_ENREF_56)); Clegg et al. (2011) ([58](#_ENREF_58));  Diepvens et al. (2008) ([62](#_ENREF_62)); Dougkas et al. (2012) ([64](#_ENREF_64));  Douglas et al. (2013) ([21](#_ENREF_21)); Hogenkamp et al. (2012) ([66](#_ENREF_66));  King et al. (2005) ([74](#_ENREF_74)); Lluch et al. (2010) ([75](#_ENREF_75));  Logan et al. (2006) ([76](#_ENREF_76)); Nobre et al. (2006) ([86](#_ENREF_86));  Ortinau et al. (2010) ([89](#_ENREF_89)); Ortinau et al. (2012a) ([90](#_ENREF_90));  rtinau et al. (2012b) ([91](#_ENREF_91)); Ortinau et al. (2013) ([92](#_ENREF_92));  Rolls et al. (1991) ([96](#_ENREF_96)); Rolls et al.(1994) ([97](#_ENREF_97));  Rolls et al. (1995) ([98](#_ENREF_98)); Smit et al. (2011) ([102](#_ENREF_102));  Tsuchiya et al. (2006) ([109](#_ENREF_109)); Vandewater & Vickers (1996) ([111](#_ENREF_111)) | Crossover | Satiation | Adults; Older adults |
| 1^†^ | Zandstra et al. (2000) ([116](#_ENREF_116)) | Crossover | Satiation | Older children |
| 7 | Burns et al. (2001) ([52](#_ENREF_52)); Burns et al. (2002) ([53](#_ENREF_53));  Chapelot & Payen (2010) ([56](#_ENREF_56)); Lluch et al. (2010) ([75](#_ENREF_75));  Logan et al. (2006) ([76](#_ENREF_76)); Rolls et al. (1991) ([96](#_ENREF_96));  Smit et al. (2011) ([102](#_ENREF_102)) | Crossover | Food intake | Adults; Older adults |
| 1^†^ | Zandstra et al. (2000) ([116](#_ENREF_116)) | Crossover | Food intake | Older children |
| 14 | Burns et al. (2000) ([51](#_ENREF_51)); Burns et al. (2001) ([52](#_ENREF_52));  Diepvens et al. (2008) ([62](#_ENREF_62)); Dougkas et al. (2012) ([64](#_ENREF_64));  Hogenkamp et al. (2012) ([66](#_ENREF_66)); King et al. (2005) ([74](#_ENREF_74));  Lluch et al. (2010) ([75](#_ENREF_75)); Nobre et al. (2006) ([86](#_ENREF_86));  O'Donovan et al. (2003) ([87](#_ENREF_87)); Rolls et al.(1994) ([97](#_ENREF_97));  Rolls et al. (1995) ([98](#_ENREF_98)); Smit et al. (2011) ([102](#_ENREF_102));  Sullivan et al. (1989) ([105](#_ENREF_105)); Tsuchiya et al. (2006) ([109](#_ENREF_109)) | Crossover | Energy intake | Adults; Older adults |
| 8 | Burns et al. (2000) ([51](#_ENREF_51)); Burns et al. (2001) ([52](#_ENREF_52));  assey (1984) ([78](#_ENREF_78)); McNamara et al. (1989) ([79](#_ENREF_79));  Rolls et al. (1991) ([96](#_ENREF_96)); Rolls et al.(1994) ([97](#_ENREF_97));  Rolls et al. (1995) ([98](#_ENREF_98)); Sullivan et al. (1989) ([105](#_ENREF_105)) | Crossover | Nutrient intake | Adults; Older adults |
| ***Metabolic health*** | | | | |
| 3 | Beydoun et al. (2008) ([48](#_ENREF_48)); Kim (2013) ([118](#_ENREF_118)); Troy et al. (2010) ([119](#_ENREF_119)) | Cross-sectional | Metabolic disorder/metabolic syndrome | Adults |
| ***Cardiovascular health*** | | | | |
| 12 | Azadbakht et al. (2013) ([45](#_ENREF_45)); Buyuktuncer et al. (2013) ([7](#_ENREF_7));  Dawczynski et al. (2013) ([122](#_ENREF_122)); Dawczynski et al. (2013) ([59](#_ENREF_59));  Gouni-Berthold et al. (2012) ([126](#_ENREF_126)); Khandelwal et al. (2009) ([132](#_ENREF_132)); Korpela et al. (2006) ([133](#_ENREF_133)); Mensink et al. (2002) ([82](#_ENREF_82));  Niittynen et al. (2008) ([137](#_ENREF_137)); Oosthuizen et al. (1998) ([88](#_ENREF_88));  Radler et al. (2011) ([138](#_ENREF_138)); Sadrzadeh-Yeganeh et al. (2010) ([143](#_ENREF_143)) | RCT | Serum lipids | Adults; Older adults |
| 7 | Chien et al. (2004) ([57](#_ENREF_57)); Dawczynski & Jahreis (2009) ([19](#_ENREF_19));  Hepner et al. (1979) ([127](#_ENREF_127)); Javed et al. (2013) ([131](#_ENREF_131));  Thompson et al. (1982) ([107](#_ENREF_107)); Trapp et al. (1993) ([108](#_ENREF_108));  Trautwein et al. (2005) ([148](#_ENREF_148)) | Cohort | Serum lipids | Adults; Older adults |
| 4 | Snijder et al. (2007) ([103](#_ENREF_103)); Steffen et al. (2005) ([145](#_ENREF_145));  Wang et al. (2008) ([149](#_ENREF_149)); Wang et al. (2012) ([150](#_ENREF_150)) | Cohort | Hypertension | Adults; Older adults |
| 3 | Iso et al. (1999) ([128](#_ENREF_128)); Larsson et al. (2009) ([134](#_ENREF_134));  Soedamah-Muthu et al. (2012) ([26](#_ENREF_26)) | Cohort | Stroke/coronary heart disease risk | Adults; Older adults |
| 4 | Freudenheim et al. (1991) ([123](#_ENREF_123)); Ivey et al. (2011) ([129](#_ENREF_129));  Kim (2013) ([118](#_ENREF_118)); Wang et al. (2013) ([113](#_ENREF_113)) | Cross-sectional | Serum lipids | Adults; Older adults |
| 4 | Ganji & Kafai (2004a) ([124](#_ENREF_124)); Masala et al. (2008) ([135](#_ENREF_135));  Meneton et al. (2013) ([80](#_ENREF_80)); Sorenson et al. (2004) ([41](#_ENREF_41)) | Cross-sectional | Hypertension | Adults; Older adults |
| 1^†^ | Ganji & Kafai (2004b) ([125](#_ENREF_125)) | Cross-sectional | Hypertension | Adults; Older children |
| 1^†^ | Zhang et al. (2011) ([151](#_ENREF_151)) | Cross-sectional | Hypertension | Older children |
| 12 | Amir Shaghaghi (2012) ([120](#_ENREF_120)); Bazzarre et al. (1983) ([46](#_ENREF_46));  Clegg et al. (2011) ([58](#_ENREF_58)); Jaspers et al. (1984) ([130](#_ENREF_130));  Massey (1984) ([78](#_ENREF_78)); McNamara et al. (1989) ([79](#_ENREF_79));  Nakamura et al. (2002) ([136](#_ENREF_136)); Niittynen et al. (2008) ([137](#_ENREF_137));  Rudkowska et al. (2007) ([141](#_ENREF_141)); Rudkowska et al. (2008) ([142](#_ENREF_142));  Sullivan et al. (1989) ([105](#_ENREF_105)); Trautwein et al. (2004) ([147](#_ENREF_147)) | Crossover | Serum lipids | Adults; Older adults |
| 2 | Cramer et al. (1989) ([18](#_ENREF_18)); Recio et al. (2011) ([139](#_ENREF_139)) | Case-control | Hypertension | Adults; Older adults |
| ***Gastrointestinal health*** | | | | |
| 4 | Bhatnagar et al. (1998) ([152](#_ENREF_152)); Boudraa et al. (2001) ([155](#_ENREF_155));  Boudraa et al. (1990) ([154](#_ENREF_154)); Pashapour & Iou (2006) ([12](#_ENREF_12)) | RCT | Diarrhoea symptoms | Young children; Infants |
| 2 | Rafeey et al. (2008) ([168](#_ENREF_168)); Ranasinghe et al. (2007) ([169](#_ENREF_169)) | RCT | Diarrhoea symptoms | Young children |
| 1^†^ | Boudraa et al. (1989) ([153](#_ENREF_153)) | RCT | Diarrhoea symptoms | Infants |
| 3 | Isakov et al. (2013) ([161](#_ENREF_161)); Nakamura et al. (2000) ([163](#_ENREF_163));  Trapp et al. (1993) ([108](#_ENREF_108)) | Cohort | Constipation | Adults; Older adults |
| 2 | Niv et al. (1963) ([165](#_ENREF_165));Karabocuoglu et al. (1993) ([162](#_ENREF_162)) | Cohort | Diarrhoea | Young children; Infants |
| 3 | Clegg et al. (2011) ([58](#_ENREF_58)); Frank et al. (2012) ([159](#_ENREF_159));  Haenni et al. (2009) ([160](#_ENREF_160)) | Crossover | Gastrointestinal transit time | Adults |
| ***Cancer*** | | | | |
| 7 | Boutron et al. (1996) ([172](#_ENREF_172)); Gallus et. al. (2006) ([177](#_ENREF_177));  Juarranz Sanz et al. (2004) ([182](#_ENREF_182)); Kampman et al. (2000) ([183](#_ENREF_183)); Karagianni et al. (2010) ([185](#_ENREF_185)); Ornelas et al. (2007) ([196](#_ENREF_196));  Peters et al. (1992) ([199](#_ENREF_199)) | Case-control | Colorectal cancer/adenoma | Adults; Older adults |
| 5 | Cook-Mozaffari et al. (1979) ([173](#_ENREF_173)); Djonovic & Arsenijevic (2011) ([174](#_ENREF_174)); Gallus et. al. (2006) ([177](#_ENREF_177)); Heck et al. (2008) ([179](#_ENREF_179));  Kawakita et al. (2012) ([186](#_ENREF_186)) | Case-control | Aerodigestive/esophageal/larynx | Adults; Older adults |
| 3 | Cramer et al. (1989) ([18](#_ENREF_18)); Faber et al. (2012) ([176](#_ENREF_176));  Gallus et. al. (2006) ([177](#_ENREF_177)) | Case-control | Ovarian cancer | Adults; Older adults |
| 2 | Hsu et al. (2007) ([180](#_ENREF_180)); Janoutova et al. (2007) ([181](#_ENREF_181)) | Case-control | Kidney cancer | Adults; Older adults |
| 5 | Kocic et al. (1997) ([189](#_ENREF_189)); Le et al. (1986) ([191](#_ENREF_191));  Reyhani et al. (2012) ([201](#_ENREF_201)); Ronco et al. (2002) ([202](#_ENREF_202));  van't Veer et al. (1989) ([205](#_ENREF_205)) | Case-control | Breast cancer | Adults; Older adults |
| 4 | Kesse et al. (2005) ([188](#_ENREF_188)); Kojima et al. (2004) ([190](#_ENREF_190));  Lin et al. (2005) ([192](#_ENREF_192)); Murphy et al. (2013) ([195](#_ENREF_195)) | Cohort | Colorectal cancer/adenoma | Adults; Older adults |
| 2 | Kampman et al. (1994) ([183](#_ENREF_183)); Pala et al. (2011) ([197](#_ENREF_197)) | Case-cohort | Colorectal cancer | Adults; Older adults |
| 2 | Kesse et al. (2006) ([188](#_ENREF_188)); Kurahashi et al. (2008) ([10](#_ENREF_10)) | Case-cohort | Prostate cancer | Adults; Older adults |
| ***Diabetes*** | | | | |
| 6 | Choi et al. (2005) ([207](#_ENREF_207)); Grantham et al. (2013) ([209](#_ENREF_209));  Kirii et al. (2009) ([210](#_ENREF_210)); Liu et al. (2006) ([211](#_ENREF_211));  Margolis et al. (2011) ([77](#_ENREF_77)); Soedamah-Muthu et al. (2012) ([26](#_ENREF_26)) | Population-based cohort | Diabetes risk | Adults; Older adults |
| 2^‡^ | O'Connor et al. (2014) ([6](#_ENREF_6)); Sluijs et al. (2012) ([212](#_ENREF_212)) | Case-cohort | Diabetes risk | Adults; Older adults |
| ***Other outcomes*** | | | | |
| 2 | Kyrozis et al. (2013) ([214](#_ENREF_214)); Chen et al. (2007) ([213](#_ENREF_213)) | Cohort | Parkinson’s disease risk | Adults; Older adults |
| 2 | Bonthuis et al. (2010) ([121](#_ENREF_121)); Soedamah-Muthu et al. (2012) ([26](#_ENREF_26)) | Cohort | All-cause mortality risk | Adults; Older adults |
| 2^‡^ | Martinez-Puig et al. (2013) ([222](#_ENREF_222)); Morina et al. (2013) ([223](#_ENREF_223)) | RCT | Joint function | Adults; Older adults |
| 1^†^ | Maslova et al. (2012) ([219](#_ENREF_219)) | Prospective cohort | Respiratory complaints | Adults; Older children |
| 1^†^ | Miyake et al. (2010) ([220](#_ENREF_220)) | Prospective cohort | Respiratory complaints | Young children; Infants |

*Further information from the study authors may be required to ensure no pseudoreplication of participants; ^†^Could be included in a meta-analysis of a wider age group; ^‡^Further information required from authors to ensure that the intervention groups were comparable.

RCT, randomized controlled trial.

**Supplementary Table B: Databases and resources searched**

| **Database** | **Interface** |
| --- | --- |
| MEDLINE and MEDLINE In-Process | OvidSP |
| EMBASE | OvidSP |
| Cochrane Database of Systematic Reviews (CDSR) | Cochrane Library/Wiley Interscience |
| Cochrane Central Register of Controlled Trials (CENTRAL) | Cochrane Library/Wiley Interscience |
| DARE Database of Abstracts of Reviews of Effects (DARE) | CRD interface |
| Health Technology Assessment Database (HTA) | CRD interface |
| Science Citation Index (SCI) | Web of Knowledge |
| Conference Proceedings Citation Index – Science (CPCI-S) | Web of Knowledge |
| African Index Medicus | http://indexmedicus.afro.who.int/ |
| LILACS | http://search.bvsalud.org/portal/ |
| OAISTER | http://oaister.worldcat.org/ |
| OpenGrey | http://www.opengrey.eu/ |
| NTIS | Proquest Dialog |
| Biosis | Proquest Dialog |
| CAB Abstracts | Proquest Dialog |
| Food Science and Technology Abstracts | Proquest Dialog |
| ClinicalTrials.gov | www.clinicaltrials.gov |
| ICTRP (trials) | http://www.who.int/ictrp/en/ |

**Supplementary Table C: Data extracted from the included studies**

| Subheading | Information required |
| --- | --- |
| Study Identification | Reference (full citation) |
| Study Details | Publication type  Study location  Study dates  Study design  Inclusion/exclusion criteria  Numbers randomised/enrolled into the study  Intervention details  Number analysed in intervention arm  Duration of intervention  Comparator details  Number analysed in comparator arm(s) |
| Participant Characteristics | Population baseline characteristics  Age group |
| Outcomes | Eligible outcomes  Eligible outcome measures  Comments |

**Supplementary Table D: Summary table for yogurt studies assessing bone health outcomes**

| **Reference** | **Study**  **design** | **Total sample size: (Intervention; Comparator)** | **Intervention details (duration)** | **Comparator details**  **(duration)** | **Eligible outcomes** | **Comments** | **Age group** |
| --- | --- | --- | --- | --- | --- | --- | --- |
| Arslantas  et al. (2008) ([17](#_ENREF_17)) | Cross-sectional | 1437 (NR; NR) | Yogurt; NR | Milk vs cheese; NR | Bone health | Non yogurt consumption is significantly correlated with occurrence of osteoporosis  (p < 0.001.) | Adults;  Older adults |
| Bener &  El Ayoubi (2012) ([32](#_ENREF_32)) | Observational cohort hospital based | 635 (NR; NR) | Yogurt consumption; NR | Other foods including dairy,  as part of a food frequency questionnaire; NR | Bone health; Nutrient status | Intake of yogurt was significantly lower in women with breast cancer with Vitamin D deficiency and in women with breast cancer with osteoporosis/osteopenia | Adults  (18-65 yr) |
| Berberidis et al. (2004) ([33](#_ENREF_33)) | Cross- sectional | 639 (NR; NR) | Yogurt; NR | Meat vs milk vs white cheese vs eggs vs fruits vs vegetables; NR | Bone health | No association between yogurt consumption and bone density | Older adults (65+) |
| Bonjour  et al. (2013) ([20](#_ENREF_20)) | Randomized double-blind controlled trial | 59 (27; 32) | Control yogurt, Yoplait France  (0 g supplemental vitamin D3 and  280 mg calcium;  2 daily yogurts of  125 g each);  ( 56 days) | Fortified yogurt, Yoplait France (10 µg supplemental vitamin D3 and 800 mg calcium; 2 daily yogurts of 125 g each); (56 days) | Bone health | At day 56 markers of bone resorption increased in both groups from baseline, a stronger response was detected in the fortified yogurt group. | Older adults (65+) |
| Feart et al. (2013) ([34](#_ENREF_34)) | Population based cohort | 1482 (NR; NR) | Yogurt; NR | Other various foods (identified as part of a food frequency questionnaire) | Bone health | Low consumption of yogurts  (<1 serving/day) was significantly associated with a doubled risk of fracture of the hip. | Older adults (> 65 yr) |
| Heaney  et al. (2002) ([8](#_ENREF_8)) | Randomized crossover trial | 29 (29; 29) | Fruit flavoured yogurt (Yoplait custard style, General Mills, Inc, Minneapolis, Minn),  3 servings/ day.  Total of 570 kcal  (21g protein,  96g carbohydrate, 10.5g fat);  ( between  7 and 11 days) | Jelled fruit flavoured snack (300kcal total);( between 7 and 11 days) | Bone health | Significant reduction in urinary excretion of N-telepeptide for yogurt (marker of bone reabsorbtion) compared with jellied fruit-flavoured snack within 7-10 days. | Adults  (18-65 yr) |
| Jha et al. (2010) ([35](#_ENREF_35)) | Case-control | 200 (NR; NR) | Yogurt (curd) consumption; NR | Other items as part of a food frequency questionnaire; NR | Bone health | Curd (yogurt) was associated with a protective effect against hip fracture. | Adults  (18-65 yr) |
| Motegi et al. (2001) ([36](#_ENREF_36)) | Cross- sectional | 713 (NR; NR) | Yogurt intake (Frequency of intake of yogurt in the 20s, 30s and 40s (years of age); NR | Other various food group intake (Frequency of intake of milk; cheese; small fish; eggs; cooked beans; tofu; natto; green veg and others in the 20s, 30s and 40s (years of age)); NR | Bone health | A greater proportion of subjects in the higher bone mineral density group had eaten yogurt 1-2 times/week when they were in their 20s, 30s and 40s, and there were more women in the low bone mineral density group who had seldom eaten yogurt in their 30s and 40s. | Adults  (18-65 yr) and Older adults  (>65 yr) |
| Nasrollahi  et al. (2012) ([37](#_ENREF_37)) | Cross- sectional | 500 (NR; NR) | Yogurt consumption; NR | Other items, including dairy as part of a questionnaire; NR | Osteoporosis risk | A statistically significant relationship between osteoporosis and 'pattern of yogurt drink'. Direction of association is not reported. | NR - Likely to be Adults and Older adults  (>65 yr) |
| Sahni et al. (2012) ([38](#_ENREF_38)) | Population based cross- sectional | 831 (NR; NR) | Yogurt; NR | Other various food items, including total dairy, milk cheese and cream (part of a food frequency questionnaire); NR | Bone health | Yogurt intakes were positively associated with multiple bone mineral density sites; still significant when adjusted for age, weight, height, sex and total energy intake; in the high vitamin D group, yogurt intakes were positively associated only with LS-BMD both in Model 1 and full-models. | Older adults (> 65 yr) |
| Sahni et al. (2013a) ([39](#_ENREF_39)) | Cohort | 3212 Framingham Offspring study members for the hip fracture analyses and 2733 members for the BMD analyses;  (NR; NR) | Yogurt; NR | Milk, cheese, cream, most dairy (total dairy without cream) and fluid dairy (milk + yogurt); NR | Bone health | Yogurt intake was associated with trochanteric bone mineral density alone. Yogurt intake showed a weak non-significant protective trend for hip fracture. | Adults  (18-65 yr) and Oder adults  (> 65 yr) |
| Sahni et al. (2013b) ([40](#_ENREF_40)) | Population based cohort | 597 (NR; NR) | Yogurt; NR | Other various food items, including total dairy, milk cheese and cream (part of a food frequency questionnaire); NR | Bone health | No relations were seen for yogurt intakes and % change in any bone mineral density sites. | Older adults (> 65 yr) |
| Sorenson  et al. (2004) ([41](#_ENREF_41)) | Cross- sectional | 1000 (Approximately) (NR; NR) | Yogurt; NR | Other dairy products (as part of a questionnaire asking about intake of 15 dairy products); NR | Association with disease (heart disease, cancer, diabetes, hypertension, osteoporosis and obesity) | No negative associations were found in this study other than yogurt in men. Lacking detail of exact outcome association with yogurt. | Older adults (>65 yr) |
| Uenishi & Nakamura (2010) ([42](#_ENREF_42)) | Cross- sectional | 38719 (NR; NR) | Yogurt; NR | Milk and cheese; NR | Bone health | After adjusting for exercise frequency, weight, gender, age, and area of residence, regression analyses indicated that yogurt intake was independently associated with OSI and QUS values (bone stiffness). | Older children  (6-18 yr) |

BMD, bone mineral density; LS-BMD, lower spine-BMD; NR, not reported; OSI, osteo sono-assessment index; QUS, quantitative ultrasound.

**Supplementary Table E: Summary table for yogurt studies assessing weight management and nutritional health outcomes**

| **Reference** | **Study**  **design** | **Total sample size: (Intervention; Comparator)** | **Intervention details (duration)** | **Comparator details**  **(duration)** | **Eligible outcomes** | **Comments** | **Age group** |
| --- | --- | --- | --- | --- | --- | --- | --- |
| Albertson  et al. (2007) ([43](#_ENREF_43)) | Cross- sectional | 8552 (4.4% (approximately 376); 96% (approximately 8176)) | Yogurt eater; NR. | Non yogurt eater; NR. | Nutrient intake; weight management | Yogurt eaters had more favourable nutrient intake profiles with lower percent of daily energy from fat and higher amounts of calcium, potassium, vitamin A, vitamin C and riboflavin. Males and female yogurt eaters had significantly lower BMI and waist circumference and females had a significantly lower percent overweight. | Adults (18-65 yr) and Older adults  (> 65 yr) |
| Almiron-Roig et al. (2009) ([44](#_ENREF_44)) | Randomized, crossover trial | 30 (30; 30) | Strawberry flavoured drinking yogurt enriched with fibre, mainly inulin and pectin (167 g); a strawberry flavoured regular drinking yogurt (184 g). Given as a pre-load to a meal; One serving. | Fresh banana (124 g); three plain crackers  (24 g); an isovolumetric serving (compared with yogurt, 160 g) of non-carbonated water. Given as a pre-load to a meal; one serving | Weight management | Yogurts were more satiating than water and crackers; Only the fibre-enriched yogurt produced higher satiety scores than crackers at 60 min; yogurt significantly reduced consumption over crackers did at the next meal; For all variables except thirst, there was a consistent tendency for yogurt to produce higher satiety ratings and lower appetite ratings; liking ratings. | Adults  (18-65 yr) |
| Azadbakht  et al. (2013) ([45](#_ENREF_45)) | Randomized controlled clinical trial | 68 (34; 34) | Yogurt as a preload before lunch and dinner, 0.5-1 serving, with 2 servings of vegetable salad and 1 glass of water; as part of a calorie restricted diet (-200 to -500 kcal/day); 3 months. | Yogurt with lunch and dinner,  0.5-1 serving and 2 servings of vegetable salad; as part of a calorie restricted diet (-200 to  -500 kcal/day);  3 months | Cardiovascular disease risk; weight management | Consuming the preload (yogurt, vegetables and water) compared with the meal (yogurt and vegetables) leads to a significant increase in percent body weight decrease and waist circumference decrease. Subjects in the preload group ate significantly less calories and carbohydrate, but not fat or fibre, than in the with-meal group. | Adults (18-65 yr) |
| Bazzarre et al. (1983) ([46](#_ENREF_46)) | Crossover trial | 30, 21 completers  (30; 30) | Yogurt, 3 x 8 oz servings of Danone low fat yogurt at morning, noon and evening) in addition to normal diet; 1 week | Normal diet; Normal diet with  3 x 250 mg calcium supplement pills at morning, noon and evening meals; 1 week | Cardiovascular risk factors; Weight management | Mean body weight measured at weekly intervals did not change significantly for males or females. | NR - likely to be Adults (18-65 yr) |
| Bener &  El Ayoubi (2012) ([32](#_ENREF_32)) | Observational cohort hospital based | 635 (NR; NR) | Yogurt consumption; NR | Other foods including dairy,  as part of a food frequency questionnaire; NR | Bone health; Nutrient status | Intake of yogurt was significantly lower in women with breast cancer with Vitamin D deficiency and in women with breast cancer with Osteoporosis/osteopenia | Adults (18-65 yr) |
| Berkey  et al. (2009) ([47](#_ENREF_47)) | Prospective Cohort | 5101 (NR; NR) | Yogurt; NR | Milk vs cheese; NR | Growth | Yogurt consumption was associated with greater height growth. | Older children (6-18 yr) |
| Beydoun  et al. (2008) ([48](#_ENREF_48)) | Cross-sectional | 17061, 4519 with complete datasets (NR; NR) | Yogurt; NR | Dairy products (including cheese, low-fat milk, whole milk, fluid milk, skim milk) and nutrients (including phosphorous); NR | Obesity, central obesity, and metabolic syndrome | Significant inverse association between intake of yogurt and obesity and central obesity. Results also analysed by ethnic group, sex and age. | Adults (18-65 yr) |
| Blom et al. (2011) ([49](#_ENREF_49)) | Randomized, double-blind, placebo-controlled, parallel-group. | 42 (20; 22) | Raspberry-flavoured yogurt drink; twice daily,  1 hour before breakfast and dinner; 15 days | Raspberry-flavoured yogurt drink that contained  1110 mg HgPE per serving. Twice daily, 1 hour before breakfast and dinner;  15 days | Weight management | Mean effects on *ad libitum* energy intakes and body weights did not differ significantly between the HgPE yogurt and yogurt-only treatment groups (p > 0.05); the number of TEAEs reported in the HgPE group was higher than in the placebo group. | Adults (18-65 yr) |
| Bonet Serra et al. (2008) ([50](#_ENREF_50)) | Double-blind case-controlled | 39 (NR; NR) | Yogurt (liquid, 200 g, supplemented with 3 g of CLA); 16 weeks | Placebo;  16 weeks | Metabolic disorder risk factors; Cardiovascular risk factors; Weight management | No significant differences were observed in weight, height or BMI between the placebo group and the CLA-supplemented yogurt group at any time point. | Adults (18-65 yr) |
| Burns et al. (2000) ([51](#_ENREF_51)) | Two;  double-blind, placebo-controlled, within-subject crossover | Study 1: 29 (29; 29); Study 2: 30 (30; 30) | Control yogurt (200 g with 6 g milk fat) 1300 h; one serving | Test yogurt  (200 g with 5 g  of a novel fat emulsion + 1 g milk fat) at  300 h; one serving | Weight management; Nutrient intake | Mean energy intakes and corresponding fat, protein and carbohydrate intakes were significantly lower after the yogurt with fat emulsion compared with the non-supplemented yogurt in each separate study, and for both studies combined. | Adults (18-65 yr) |
| Burns et al. (2001) ([52](#_ENREF_52)) | Randomized double-blind, placebo-controlled, within-subject crossover design | 60 (60; 60) | Control yogurt (Skane Mejerier, Lunnarp, Sweden) as a breakfast (200 g); one serving | Yogurt with Olibra TM fat (Skane Mejerier, Lunnarp, Sweden) as a breakfast (200 g); one serving | Weight management; Nutrient intake | Mean energy intakes and corresponding macronutrient intakes were significantly lower after the Olibra fat yogurt compared with the non-supplemented yogurt in non-overweight and overweight subjects 4 h post-consumption, and in all subjects 8 h post-consumption. In the total group, energy intakes over the following 24 h were also significantly reduced after the Olibra fat yogurt relative to the non-supplemented yogurt. For all subjects, energy, fat, protein and carbohydrate intakes and the total weight of food consumed were significantly reduced at 4 and at 8 h post-consumption of the test yogurt, relative to the control yogurt. | Adults (18-65 yr) |
| Burns et al. (2002) ([53](#_ENREF_53)) | Randomized, single-blind, placebo controlled, within-subject crossover | 50 (50; 50) | Yogurt, 200 g; One serving containing 15 g of milk fat (40% fat emulsion), as a pre-load to a meal; One serving | 200 g portion of yogurt containing a total of 15 g of fat, which varied in quantity of OlibraTM fat (2, 4, 6 g), as a pre-load to a meal; one serving | Weight management | Yogurt with Olibra fat reduced the effect of overeating during an *ad libitum* lunch meal and subsequent food intake up to 36 h post-consumption, compared with control yogurt. No significant difference between yogurts in hunger, desire to eat or perceived fullness. | Adults (18-65 yr) |
| Buyuktuncer et al. (2013) ([7](#_ENREF_7)) | Randomized, double-blind, placebo-controlled | 70 (35; 35) | 115 g/day spoonable low-fat yogurt (Yıldız Holding), in addition to normal diet; 4 weeks | 115 g/day spoonable low-fat yogurt with 1.9 g plant stanols as esters (Yıldız Holding), in addition to normal diet; 4 weeks | Cardiovascular risk factors; Weight management | No clinically significant change in anthropometrical measurements was observed during the intervention. | Adults (18-65 yr) |
| Campbell  et al. (1999) ([54](#_ENREF_54)) | Randomized trial | 16 (9; 7) | Low-fat, vanilla (2,092 kJ, 90 g carbohydrate, 8 g fat, 18 g protein) or plain (1,422 kJ, 36 g carbohydrate, 9 g fat, 26 g protein), non-pasteurized yogurt (Darigold, Issaquah, WA), containing a commercially produced yogurt culture strain, daily. The yogurt culture was a 1:1 ratio of L. delbruekeii ssp. bulgaricus and  S. salivarius ssp. thermophilus. Serving size was two cups  (454 g); approximately  3 months | No yogurt products; approximately  3 months | Nutrient intake; oestrogen metabolism | No significant differences were observed within or between groups. | Adults (18-65 yr) |
| Campbell  et al. (2000) ([55](#_ENREF_55)) | Randomized trial | 25 (13; 12) | Low-fat, vanilla or plain, nonpasteurized yogurt (Darigold, a 1:1 ratio of  L. delbrueckii ssp. bulgaricus and  S. salivarius ssp. thermophilus (>107 colony-forming units/g yogurt)), containing a commercially produced yogurt culture strain,  daily throughout three menstrual cycles. Serving size was two cups (454 g) daily; 4 months | No yogurt products; 4 months | Nutrient intakes | Macronutrient intakes differed between groups only for carbohydrate. Calcium intake increased for yogurt consumers during intervention. Linoleic acid intake increased for control and decreased for yogurt. | Adults (18-65 yr) |
| Chapelot & Payen (2010) ([56](#_ENREF_56)) | Randomized crossover | 48 (48; 48) | Yogurt as a pre-load to a meal: The ‘gouˆter’ consisted of either a strawberry-flavoured liquid yogurt commercialized under the name of Yopw (Yoplait Co); one serving | A popular chocolate bar (Twix; Mars Inc., McLean, VA, USA) as a pre-load to a meal; one serving | Weight management | Yogurt induced a lower subjective motivation to eat than chocolate bars during the hour preceding the spontaneous onset of a meal, without affecting subsequent food intake; these differences in satiety ratings at dinner request were not followed by a consistent difference in intake. The duration of post-‘gouˆter’ satiety did not differ between the test products, i.e. about 170 min long for both foods. | Adults (18-65 yr) |
| Chien et al. (2004) ([57](#_ENREF_57)) | Cohort | 70 (NR; NR) | Yogurt commercially packaged meals; total energy intake was 1200 kcal/day; 6 weeks | Traditional low- calorie diet; total energy intake was 1200 kcal/day;  6 weeks | Weight management; Cardiovascular risk factors | The average weight loss in yogurt meal group was 0.9 kg/week significantly less than the traditional diet group. | Adults (18-65 yr) |
| Clegg et al. (2011) ([58](#_ENREF_58)) | Randomized single-blind crossover trial | 11 (11; 11; 11) | Low fat yogurt, 260 g once daily, in addition to a normal diet; 3 days | High-fat yogurt (260 g yogurt with 90 g of sunflower oil), once daily,  in addition to a normal diet; No additional yogurt; 3 days | Weight management; Cardiovascular risk factors | There were no differences in satiety between the interventions. | Adults (18-65 yr) |
| Dawczynski et al. (2013) ([59](#_ENREF_59)) | Randomized, placebo-controlled, double-blind, parallel designed | 47 (14; Group 2= 17; Group 3= 16) | Commercial fruit yogurt (125 g, 3.5% w/w fat);  10 weeks | Group 2 = 125 g yogurt with approx. 3.5% fat (approx. 2.5% fish oil and 1% milk fat), 0.8 g of n-3 PUFA vs Group 3 = 200 g yogurt with approx. 5.5% fat (approx. 5% fish oil and 0.5% milk fat), 3 g of  n-3 PUFA;  10 weeks | Cardiovascular risk factors; Micronutrient intake | No significant changes in cardiovascular risk factors or blood cholesterol levels were observed in the non-supplemented yogurt group when comparing week 0 to week 10. No significant difference in micronutrient intake between baseline and week 10 for the non-supplemented yogurt group, except for protein intake (decrease). | Adults (18-65 yr) |
| Dewan et al. (2007) ([60](#_ENREF_60)) | Randomized controlled | 68 (32 completers;  36 completers) | Curd (yogurt) 200 g with 6 g of protein per day;  15 days | Micronutrient-rich leaf protein concentrate (LPC) in a powder sachet, 6 g of protein per day; 15 days | Recovery from malnutrition | A significant average increase in weight was seen in both the groups; haemoglobin rose significantly in both the groups after supplementation. There was a significant fall in serum ferritin levels in both the groups at the end of the study period. | Young children (1-5 yr) |
| Dewan et al. (2009) ([61](#_ENREF_61)) | Randomized control trial | 68 (40,  32 completers; 40,  36 completers) | Curd (Indian Dahi) in addition to WHO recommended two-step diet for severe malnutrition, modified according to local dietary habits; 15 days | LPC in addition to WHO recommended two-step diet for severe malnutrition, modified according to local dietary habits; 15 days | Recovery from malnutrition | Weight increased significantly in both yogurt and leaf protein concentrate groups. | Young children (1-5 yr) |
| Diepvens  et al. (2008) ([62](#_ENREF_62)) | Randomized, double-blind, placebo-controlled, crossover design | 41 (41; 41) | Placebo yogurt (250 g yogurt containing 4.2 g milk fat) as breakfast consumed within 5 minutes; one serving | 250 g yogurt containing  0.2 g milk fat and 4 g vegetable fat, provided by adding 10 g Olibra® emulsion (Lipid Technologies Provider AB, Karlshamn, Sweden) to the yogurt, as breakfast consumed within 5 minutes; one serving | Weight management | In the junior-normal weight subjects, consumption of test yogurt reduced hunger and desire to eat during the morning and increased the time elapsed between consumption of the yogurt and the point at which hunger scores returned to baseline; in the test condition compared with the  non-supplemented yogurt. No significant differences in appetite scores between the test and  non-supplemented yogurt were seen for the senior-overweight subjects. No effect on energy intake was seen in the total group, in the junior-normal weight and senior-overweight subjects. | Adults (18-65 yr) |
| Diepvens  et al. (2007) ([63](#_ENREF_63)) | Randomized, placebo-controlled, double-blind, parallel design | 50 (28; 22) | Yogurt (250 g yogurt containing 5 g milk fat), twice daily (breakfast and afternoon); 18 weeks | Yogurt (250 g) containing 3 g milk fat and  2 g vegetable fat, provided by  5 g Olibra emulsion, twice daily (breakfast and afternoon); 18 weeks | Weight management | The milk fat yogurt group gained weight, were hungrier than vegetable fat yogurt and showed increased satiety hormone values 180 min after yogurt consumption. Fat mass was significantly more decreased in the vegetable fat yogurt group compared to the milk fat yogurt group; BMI and waist circumference did not increase in the test group, but significantly increased in the placebo group  (p < 0.05) during the weight maintenance period; GLP-1 values at 180 min after yogurt consumption were significantly increased at week 25 compared with week 1 in the test group, but not in the placebo group (treatment over time effect, p < 0.05) | Adults (18-65 yr) |
| Dougkas  et al. (2012) ([64](#_ENREF_64)) | Randomized crossover trial | 40 (40; 40) | Natural set biopot yogurt (Dr Oetker) as a pre-load to a meal; one serving | Isoenergetic semi-skimmed milk (Cravendale; Arla Foods), a mild Cheddar cheese (Sainsbury’s); isovolumetric serving (compared with milk) of non-carbonated water as a pre-load to a meal; one serving | Weight management; Diabetes risk factors | Hunger rating was lower after the intake of yogurt than cheese, milk and water, respectively. Energy intake was lower after the intake of yogurt compared with water. Yogurt had the greatest effect on suppressing subjective appetite ratings, but did not affect subsequent food intake compared with milk or cheese. | Adults (18-65 yr) |
| Douglas  et al. (2013) ([21](#_ENREF_21)) | Randomized crossover design | 22 (15; 15) | Afternoon yogurt snack (160 kcal) containing Low Protein (LP, 5 g protein), Moderate Protein (MP,  14 g protein), and High Protein (HP, 24 g protein), with 8 oz. of water. Specifically, the LP yogurt was 14% protein,  76% CHO, and 10% fat; the MP yogurt was  6% protein, 64% CHO, and 0% fat; and, the HP yogurt contained  60% protein, 40% CHO, and 0% fat. Yogurts were given as a pre-load to a meal; One serving (after 3 acclimation days with 4 oz water) | No snack and  8 oz. of water as a pre-load to a meal; one serving (after  3 acclimation days with 4 oz water) | Weight management | An afternoon snack of Greek yogurt, containing 24 g protein, led to reduced hunger, increased fullness, and delayed subsequent eating compared to lower protein snacks in healthy women. | Adults (18-65 yr) |
| Hoffman  et al. (2000) ([65](#_ENREF_65)) | Case-control with crossover trial | 56 (56; 56) | Yogurt supplement (small cup of yogurt (753 kJ)  2–3 min after breakfast);  3 days, 1 day with supplement | No yogurt supplement;  3 days | Weight management | No significant effect of yogurt breakfast supplement on daily energy intake in either stunted or non-stunted children. | Older children (6-18 yr) |
| Hogenkamp et al. (2012) ([66](#_ENREF_66)) | Randomized crossover trial | 12 (12; 12) | Pre-load before breakfast was: 300 g of low-calorie yogurt (60 kcal/100 g) including 1.92 g sweetener; 4 days (perhaps non-consecutive) | Pre-load before breakfast was: 300 g of high-calorie yogurt (177 kcal/100 g) including 66 g table sugar and 15 g sunflower oil; 4 days (perhaps non-consecutive) | Weight management | *Ad libitum* intake was lower after high-calorie yogurt preloads (as compared with low-calorie preloads). No significant difference between LC yogurt and HC yogurt in appetite rating, blood glucose, insulin or cortisol. Both preloads were provided once with LC information (LC-info) and once with HC information (HC-info). Intake after LC yogurt preloads was higher when provided with (consistent) LC information as compared wiht (inconsistent) HC-info, but intake after the HC preloads did not depend on the information provided. No significant difference between LC yogurt and HC yogurt in appetite rating, blood glucose, insulin or cortisol. | Adults (18-65 yr) |
| Hursel et al. (2010) ([67](#_ENREF_67)) | Randomized, three-arm, crossover design | 35 (35; 35) | Normal milk protein yogurt drink with whole milk protein (Friesland Foods); 15, 47 and 38% energy from protein, carbohydrate and fat, respectively; one serving | High-protein (HP) yogurt with added total whey protein; or a HP yogurt that contained caseinomacropeptide-depleted  α-lactalbumin enriched whey protein; one serving | Weight management | Resting energy expenditure did not differ between the three conditions. High protein yogurt increased diet-induced thermogenesis significantly more compared with a normal protein yogurt at breakfast. After the normal protein yogurt a positive fat balance occurred. Whey protein added to the yogurt drink is the focus of investigation; The results of the present study confirmed that high-protein breakfasts enhance thermogenesis more compared with a normal-protein breakfast. | Adults (18-65 yr) |
| Jodkowska et al. (2011) ([68](#_ENREF_68)) | Case-control | 1906 (NR; NR) | Yogurt and kefir; NR | Other foods including snack foods and dairy (yellow cheese, vegetables, fruits); NR | Weight management | The overweight teenagers less often chose snacks with high fat content, sugar and salt, and more often vegetables, fruits, as well as yogurt and kefir. Note: natural yogurt data may be grouped with kefir (not eligible). | Older children (6-18yr) |
| Jordan et al. (1981) ([69](#_ENREF_69)) | Cohort | 1056 (472 completers) | Recommended yogurt for meals and snacks (in addition to normal diet) | Behavioural instruction only vs Recommended soup for lunch vs Recommended soup for any meal or snack; 10 weeks | Weight management | No significant relationship between frequency of yogurt ingestion and weight loss, calories consumed or rate of eating. | Adults (18-65 yr) and Older adults  (> 65 yr) |
| Jordão et al. (2009) ([70](#_ENREF_70)) | Cross- sectional survey | 354 (NR; NR) | Yogurt; NR | Other foods (including dairy: liquid milk, power milk, milk based formula); NR | Anaemia | Anaemia was associated to early introduction of yogurt (p=0.005). | Infants (<1 year) |
| Joshi et al. (2011) ([71](#_ENREF_71)) | Cross- sectional | 2672 (193; 2479) | Yogurt eaters (eating yogurt at least once on a 24 hr recall diet); NR | Non yogurt eaters; NR | Nutrient intake;  Weight management | For yogurt eaters, BMI was lower by 1.3 kg/m^2^ (p=0.03), waist circumference was lower by 3.5 cm (p=0.02), body fat was lower by 1.5% (p=0.03) and waist-to-height ratio was lower by 2% (p=0.02) than non-yogurt eaters. Women who ate yogurt had significantly higher intakes of protein, vitamin D, calcium, potassium, and magnesium, compared with non-yogurt eaters (p<0.05). | Adults (18-65 yr) and Older adults  (> 65 yr) |
| Keast et al. 2010) ([72](#_ENREF_72)) | Cross- sectional | 3786 (approximately 322 (8.5%); 3464 (approximately 91.5%)) | Yogurt consumers; NR | Non yogurt consumers; NR | Nutrient intake;  Weight management | Yogurt consumption was positively associated with intake of calcium and vitamin D. Yogurt consumers had lower BMI, less overweight/obesity prevalence, smaller waist circumference and subscapular skinfold than non-yogurt consumers. These trends were seen after adjusting for gender, ethnicity, age, and energy; and also when physical activity and other covariates were added. | Older children (6-18 yr) |
| Keast et al. (2013) ([73](#_ENREF_73)) | Cross- sectional | 3786 (approximately 322 (8.5%); 3464 (approximately 91.5%)) | Yogurt consumers; NR | Non yogurt consumers; NR | Nutrient intake; Weight management | After adjusting for gender, ethnicity, age, and energy, yogurt consumption was associated with higher protein intake and lower dietary fat, with no differences in energy, carbohydrate, total sugars, and added sugar intake. Data on obesity measures are reported in the sister study by the same authors (using the same data), Keast et al 2010). | Older children (6-18 yr) |
| King et al. (2005) ([74](#_ENREF_74)) | Crossover trial | 15 (15; 15) | Yogurt, strawberry flavoured, containing  25 g sucrose/day (Lintech (Reading, UK); 200 g as a pre-load, daily; 10 days | Yogurt containing 25 g xylitol/day; Yogurt containing 25 g polydextrose/day; Yogurt containing 12.5 g xylitol and 12.5 g polydextrose/day Xylitol and Litessew polydextrose supplied by Danisco Inc. (Ardsley, NY, USA); 200g as a pre-load, daily;  10 days | Weight management | When energy content was controlled for, there was a significant suppression of energy intake for polydextrose enriched yogurt compared with non-supplemented yogurt. Xylitol-polydextrose enriched yogurt induced a significantly stronger satiating effect (increase in subjective fullness) compared with the non-supplemented yogurt (this effect was also present when energy content was not controlled for). | Adults (18-65 yr) |
| Lluch et al. (2010) ([75](#_ENREF_75)) | Single-blind, controlled trials (randomized within-subject crossover) | Study 1: 24; Study 2: 121 | Strawberry low-fat yogurt (midmorning snack, 2 h after the beginning of consumption of a fixed breakfast); one serving | Strawberry low-fat yogurt enriched with milk protein and fibre (guar gum) (Shape Lasting Satisfaction, Danone), midmorning snack, 2 h after the beginning of consumption of a fixed breakfast; one serving | Weight management | The enriched yogurt reduced subjective appetite compared to the non-enriched yogurt. Energy intake at lunch was reduced by 274 kJ after the enriched yogurt compared to the non-enriched yogurt; Overall, when combining total morning intakes, the total quantity of food and energy consumed in the enriched yogurt was significantly lower than in the non-enriched yogurt. | Adults (18-65 yr) and Older adults  (> 65 yr) |
| Logan et al. (2006) ([76](#_ENREF_76)) | Randomized, double-blind, placebo- controlled, within-subject crossover design | 28 (28; 28) | Yogurt containing milk fat (200 g at breakfast); each intervention lasted  3 weeks; there were 6 test days across each  3 weeks period | Yogurt containing Olibra fat (200 g at breakfast); each intervention lasted 3 weeks; there were 6 test days across each 3 weeks period | Weight management | No significant difference between groups. | Adults (18-65 yr) |
| Margolis  et al. (2011) ([77](#_ENREF_77)) | Prospective cohort | 82076  (NR; NR) | Yogurt; NR | High-fat dairy intake; low-fat dairy intake; total dairy intake; NR | Type 2 diabetes risk; Body weight | Yogurt consumption was associated with a decreased risk of type 2 diabetes, not significantly associated with body weight change after adjustment for energy intake. | Adults (18-65 yr) and Older adults (> 65 yr) |
| Massey (1984) ([78](#_ENREF_78)) | Crossover trial | 30 (30; 30) | Plain low fat yogurt  2 x 240 ml cartons, daily); 4 weeks | No yogurt;  4 weeks | Cardiovascular disease risk factors; Nutrient intake | Yogurt supplementation significantly increased intake of calcium and carbohydrate. Although some serum lipid means differed significantly among some sampling points, there was no effect on total cholesterol, total triglycerides, high density lipoprotein cholesterol, or distribution of electrophoretic lipoprotein fractions | Adults (18-65 yr) |
| McNamara et al. (1989) ([79](#_ENREF_79)) | Prospective crossover trial | 18 (18; 18) | Low-fat yogurt (16 oz./day) as part of a  low-fat, low-cholesterol baseline diet; 4 weeks | Non-fermented dairy product (16 oz. low-fat milk plus 10% milk solids) as part of a low-fat, low-cholesterol baseline diet; duration = 4 weeks. No yogurt supplementation to the low-fat, low- cholesterol diet;  3 weeks | Cardiovascular risk factors; Weight management; Macronutrient intake | Plasma total, LDL- and HDL-cholesterol were unaffected by either the yogurt of low-fat milk concentrate. Average body weights and dietary intakes of fat, cholesterol and polyunsaturated/saturated fat ratios were not significantly different for the 3 dietary phases. | Adults (18-65 yr) |
| Meneton  et al. (2013) ([80](#_ENREF_80)) | Cross- sectional | 6167  (NR; NR) | Yogurt; NR | Various other foodstuffs (identified by food frequency questionnaire); NR | Cardiovascular disease risk; Weight management | Overweight patients eat more yogurts than non-overweight. | Adults (18-65 yr) |
| Mensah & Otoo (2013) ([81](#_ENREF_81)) | Cross- sectional | 106 (NR; NR) | Yogurt; NR | Other various foods, including milk, cheese and ice cream (identified as part of a food frequency questionnaire); NR | Nutritional status | No significant association between yogurt and nutritional status (malnourishment) of the children. | Young children (1-5 yr) |
| Mensink  et al. (2002) ([82](#_ENREF_82)) | Double blind, placebo-controlled, parallel design | 60 (30; 30) | Low-fat yogurt (0.7% fat); 3 x 150 ml cups daily, with meals; 4 weeks | Low-fat yogurt (0.7% fat, 1 g of plant stanol per cup); 3 x 150 ml cups daily, with meals; 4 weeks | Cardiovascular risk factors; Weight management | Changes in body weight of the subjects during the experimental period were small and non-significant for the non-supplemented yogurt group. During the experimental period, serum total cholesterol and LDL cholesterol concentrations increased slightly for the non-supplemented yogurt group. HDL cholesterol and triglyceride concentrations did not change and total to HDL cholesterol ratio hardly changed in the non-supplemented yogurt group. | Adults (18-65 yr) |
| Mossavar-Rahmani et al. (2002) ([83](#_ENREF_83)) | Cross- sectional | 3396 (2173; 1223) | Yogurt (as eaten by consumers); NR | No yogurt (non-consumers of yogurt); NR | Weight management; Nutrient intake; General health | Yogurt consumers were, on average, taller and lighter than non-consumers. They also exhibited healthier behaviour including being more physically active, having a lower rate of smoking, and consuming a healthier diet, notably a significantly higher intake of calcium, fibre, vitamins A and D, lycopene, fruits, and vegetables and lower intake of total and saturated fats. | Adults (18-65 yr) and Older adults  (> 65 yr) |
| Mozaffarian et al. (2011) ([84](#_ENREF_84)) | Cohort | 120887  (NR; NR) | Yogurt; NR | Other various food; NR | Weight management | 4-year weight change was inversely associated with yogurt. | Adults (18-65) and Older adults  (> 65 yr) |
| Nazare et al. (2007) ([85](#_ENREF_85)) | Randomized double-blind placebo-controlled | 44 (23; 21) | Yogurt, Danone Company (skimmed milk inoculated with 10^7^ colony-forming units of yogurt bacillus [Streptococcus thermophilus, Lactobacillus bulgaricus]) one consumed daily;  8 days | Yogurt, Danone Company (skimmed milk inoculated with 10^7^ colony-forming units of yogurt bacillus (Streptococcus thermophilus, Lactobacillus bulgaricus) and 3·76 g triacylglycerols), one consumed daily; 98 days | Weight management | No significant difference in BMI, weight fat mass or free fat mass between baseline and end of study for non-supplemented yogurt group; no changes were observed in blood cell count, liver enzyme levels, bleeding time and prothrombin concentration; activated cephalin time was significantly increased in men from the CLA group but not in women; Thiobarbituric acid reactive substance formation was unaltered by CLA consumption, in men and in women. | Adults (18-65 yr) |
| Nobre et al. (2006) ([86](#_ENREF_86)) | Crossover trial (though unclear description) | 20 (20; 20) | Yogurt mixed with a commercially available product, Emustab,  6 g/300 ml) aerated to 300, 450 and 600 ml; one serving | N/A | Weight management | Light yogurt volumes affected the satiety of both studied groups and the greater volume was more effective. The highest hunger score was obtained after the 300 ml intake, followed by 450 and 600 ml (p<0.01). The desire for sweet, salty and fatty foods and snacks was not influenced by time and yogurt volume in either studied group. Energy intake between the days which yogurt was consumed and yogurt was not consumed was not statistically different (p<0.05). | Adults (18-65 yr) |
| O'Connor  et al. (2014) ([6](#_ENREF_6)) | Nested case-cohort | 4749  (NR; NR) | Yogurt; NR | Various food stuffs identified by food diaries; NR | Diabetes (type 2) risk;  Weight management | Inverse association between diabetes and yogurt intake. Amount of yogurt consumption did not vary with BMI or waist circumference. Substituting yogurt for snacks was associated with 47% lower hazard of type 2 diabetes. | Adults (18-65 yr) and Older adults  (> 65 yr) |
| O'Donovan et al. (2003) ([87](#_ENREF_87)) | Randomized, double- blind, crossover trial | 14 (14; 14) | High-fat (70% energy from fat) yogurt ‘preload’; one serving | High-fat (70% energy from fat) yogurt ‘preload’ (males 400 g (2562 kJ); females 300 g (1923 kJ)), which included 120 mg orlistat (Xenicalw; Hoffmann-La Roche); one serving | Weight management | Energy intake for the remainder of the day was greater following orlistat yogurt versus non-supplemented yogurt; There was no difference in total (calculated) energy intake after orlistat yogurt versus non-supplemented yogurt. Fat excretion was greater following orlistat yogurt versus non-supplemented yogurt; Change in plasma CCK levels over time showed no overall difference between orlistat supplemented yogurt versus non-supplemented yogurt. | Adults (18-65 yr) |
| Oosthuizen et al. (1998) ([88](#_ENREF_88)) | Double-blind randomized | 21 (7; 7; 7) | Frozen yogurt, 175 g/d as part of a high-fibre, low-fat diet; 4 weeks | Frozen yogurt, 175 g/d with 20 g soya bean lecithin as part of a high-fibre, low-fat diet; Frozen yogurt, 175 g/d with 17 g sunflower oil as part of a high-fibre, low-fat diet; 4 weeks | Cardiovascular risk factors; Weight management | Significant decrease from baseline levels in HDL cholesterol and LDL/HDL cholesterol in the non-supplemented yogurt group; Plasma palmitic (C16:0) and palmitoleic acid (C16:1) levels increased significantly in the yogurt group; No significant change in body weight or BMI from baseline in the non-supplemented yogurt group | Adults (18-65 yr) |
| Ortinau et al. (2010) ([89](#_ENREF_89)) | Randomized crossover trial | 46 (46; 46) | Lower-protein yogurt  (5 g protein) 160 kcal consumed 3 h post-lunch; one serving (after 3 days acclimation) | Higher-protein yogurt (14 g protein) 160 kcal consumed 3 h post-lunch; no snack; one serving (after 3 days acclimation) | Weight management | Both 160 kcal afternoon yogurt snacks led to reduced hunger, increased fullness, and delayed eating vs. no snacking, no differences were found between the low vs. higher protein yogurts. | Adults (18-65 yr) |
| Ortinau et al. (2012a) ([90](#_ENREF_90)) | Randomized crossover trial | 23 (23; 23) | Higher-protein yogurt  (14 g protein in 160 kcal, consumed 3 h post-lunch); one serving (after 3 days acclimation) | Pretzels; Crackers; Chocolate; and No Snack (consumed 3 h post-lunch); One serving (after 3 days acclimation) | Weight management | Higher protein yogurt had a greater delay in eating than chocolate and crackers. Dinner was requested later after high protein yogurt than no snack. | Adults (18-65 yr) |
| Ortinau et al. (2012b) ([91](#_ENREF_91)) | Randomized crossover trial | 46 (46; 46) | Lower-protein yogurt  (5 g protein in 160 kcal, consumed 3 h post-lunch); one serving (after 3 days acclimation) | Higher-protein yogurt (14 g protein in 160 kcal) (consumed 3 h post-lunch); no snack; one serving (after 3 days acclimation) | Weight management | Yogurt reduced hunger and increased fullness up to 2 h post-snack vs. no snack; No snack led to the earliest request for dinner vs. high-protein and low protein yogurt; no difference was found between snacks no differences were observed between high and low protein yogurts. This cohort may be similar participants in cohorts in other studies by the same author. | Adults (18-65 yr) |
| Ortinau et al. (2013) ([92](#_ENREF_92)) | Randomized crossover | 32 (32; 32) | Yogurt: normal protein  (5 g, NP) taken in the afternoon with 8 oz water, as a pre-load to a meal; one serving (after 3 days acclimation) | Higher-protein  (14 g, HP) taken in the afternoon with 8 oz water, as a pre-load to a meal; one serving (after 3 days acclimation) | Weight management | No differences in post-snack perceived hunger or fullness were observed between the normal protein and high protein yogurt snacks. Time of voluntary dinner request was not significantly different between the normal protein and high protein yogurts. *Ad libitum* dinner intake was not different between the snacks. | Adults (18-65 yr) |
| Pordeus Luna et al. (2011) ([93](#_ENREF_93)) | Cross- sectional | 170 (NR; NR) | Yogurt; NR | Various other foods (identified in a food frequency questionnaire); NR | Nutrient consumption | Children who had adequate vitamin E intake regularly consumed yogurt. | Older children (6-18 yr) |
| Pounis et al. (2010) ([94](#_ENREF_94)) | Cross- sectional | 4786 (NR; NR) | Yogurt; NR | Various other foodstuffs (identified by food frequency questionnaire); NR | Weight management | Overweight/obese participants had 2.25 times greater likelihood of consuming yogurt than normal-weight children. | Adults (18-65 yr) and Older adults  (> 65 yr) |
| Rodriguez-Artalejo  et al.(2003) ([95](#_ENREF_95)) | Cross- sectional | 1112 (NR; NR) | Yogurt; NR | Bakery products, sweetened soft-drinks; NR | Weight management | Consumption of yogurt, while associated with significantly higher energy intake from saturated fats and sugars, showed no association with the Healthy Eating index. Consumption of yogurt was significantly positively associated with total energy intake, though not with BMI. | Older Children (6-18 yr) |
| Rolls et al. (1991) ([96](#_ENREF_96)) | Single-blind, crossover trial | 28 (28; 28) | High carbohydrate, raspberry flavoured yogurt (81% carbohydrate- maltose); 350 g for females, 500 g for males; High-fat, raspberry- flavoured yogurt (65%  fat-corn oil and cream); 350 g for females, 500 g for males. Yogurts given as a preload to a meal; one serving on 3 occasions | No yogurt as a pre-load before a meal; 3 occasions | Weight management | In the 30-min delay condition, subjects accurately compensated for the calories in the preload yogurts compared with a no-preload condition, but as the interval increased to 90 and 180min, compensation was less precise. No significant differences in subsequent food intake were found between the high-carbohydrate and high-fat yogurts at any time interval. Also, there were no differences in ratings of hunger and fullness between the yogurts. The macronutrient composition of the preload yogurt did not affect the types of foods, or macronutrients, consumed at lunch. | Adults (18-65 yr) |
| Rolls et al.(1994) ([97](#_ENREF_97)) | Balanced crossover | 72 (72; 72) | Yogurt, raspberry- flavoured, containing a minimal amount of fat and carbohydrate and was relatively low in energy, consumed as a preload before a set lunch; one serving | A medium-fat yogurt; a high-fat yogurt; a medium-carbohydrate yogurt; a high-carbohydrate yogurt - all consumed as a preload before a set lunch; no yogurt preload; one serving | Weight management | Normal weight unrestrained males consumed significantly more at lunch after standard yogurt than after high fat or high carbohydrate conditions. The percent of energy consumed at lunch derived from fat and carbohydrate did not differ significantly across the six interventions for any population group; Satiety did not differ significantly across the six interventions for any population group. | Adults (18-65 yr) |
| Rolls et al. (1995) ([98](#_ENREF_98)) | Double-blind crossover repeated measures | 32 (32; 32) | Three types of yogurts (raspberry-flavoured) varying in energy and macronutrient content, matched for sensory properties. High fat (from corn oil and cream) and low carbohydrate (2134 kJ; 65% of energy as fat); high-carbohydrate (from maltose) and low-fat (2134 kJ; 81% of energy as carbohydrate); low-fat and low-carbohydrate (962 kJ). 500 g serving as a pre-load to a meal; the protein content was kept as similar as possible among the three yogurts and the same volume (500 g) of yogurt was given to all participant; one serving on 4 occasions | No yogurt as a pre-load before a meal;  4 occasions | Weight management | Young men consumed ± 10% total energy (lunch + yogurt) in the yogurt preload conditions compared with their baseline (no yogurt) intake; elderly men overate between 10% and 30% in relation to their baseline (no yogurt) intake; both differences were significant. The proportions of macronutrients consumed in the test meals did not differ significantly across preload conditions or between the young and elderly participants. Hunger showed clear changes with decreases after preloads and meals in both age groups, whereas fullness increased. | Adults (18-65 yr) and Older adults  (> 65 yr) |
| Rosado  et al. (2005) ([99](#_ENREF_99)) | Controlled trial | 48 (16; Low fat milk group = 16; Water group = 16) | Plant based test meal plus 150 g of flavoured yogurt (Danvive, Danone de Mexico), twice daily; 13 days | Plant based test meal plus 250 mL of low-fat ultra-high-temperature-treated milk; Plant based test meal plus 250 mL of water; 13 days | Nutrient absorption | Including yogurt in the diet increased zinc absorption by 68%, respectively. The total amount of zinc absorbed was increased by 78% when yogurt was added to a meal. The total amount of iron absorbed did not differ among the groups. | Adults (18-65 yr) |
| Salakidou  et al. (2012) ([100](#_ENREF_100)) | Randomized controlled trial | 60 (30; 30) | Yogurt (non-fortified),  200 g daily; 30 days | Yogurt, fortified with 200 IU of vitamin D, 200 g daily; 30 days | Micronutrient levels | At the end of the study, serum 25(OH) D levels increased significantly in both groups, but the level in the Vitamin D fortified yogurt group was higher than non-fortified yogurt group. | Adults (18-65 yr) |
| Sazawal et al. (2013) ([14](#_ENREF_14)) | Double-blind randomized controlled trial | 1010 (509, 293 completers; 501, 278 completers) | Non-fortified yogurt, 60 g daily; 1 year | Micronutrient fortified yogurt  60 g daily (providing 30% RDA for iron, zinc, iodine and vitamin A); 1 year | Growth; Nutrient status | Children in the fortified yogurt group showed improvement in Hb as compared to the non-fortified yogurt group. The fortified yogurt group had better height gain velocity and height-for-age z-scores compared with the non-fortified yogurt group. There was no difference in weight gain velocity, weight-for-age z-scores or BMI z-scores. Retinol binding protein and iodine levels decreased from baseline in both groups but the decrease was significantly less in the fortified yogurt group. | Older children (6-18 yr) |
| Schusdziarra et al. (2011) ([101](#_ENREF_101)) | Cross- sectional | 480 (NR; NR) | Yogurt; NR | Other various foods, including cheese and butter (identified by food frequency questionnaire); NR | Weight management | Yogurt contributed significantly to the observed changes of breakfast energy intake, but not intake for the whole day. Unclear reporting on which direction the trend is in. | NR - likely to be Adults (18-65 yr) and Older adults  (> 65 yr) |
| Smit et al. (2011) ([102](#_ENREF_102)) | Balanced, randomized, placebo-controlled, and double-blind crossover | 24 (24; 24) | Yogurt-based meal replacement drink  (325 ml, containing 5 g of milk fat and corn oil);  one serving | Yogurt-based meal replacement drink (325 ml with 12.5 g Fabuless (5 g fat; added during manufacture, thus ‘processed’), Yogurt-based meal replacement drink (325 ml with 12.5 g Fabuless (5 g fat; added at the end of manufacture, thus ‘unprocessed’); one serving | Weight management | No effect on appetite or satiety after any of the yogurt drinks. Neither energy intake nor food intake at lunch were significantly affected by either of the Fabuless yogurt drinks when compared with non-supplemented yogurt drink. Energy intake at dinner was significantly lower following the unprocessed Fabuless yogurt drink compared with non-supplemented yogurt, but not following processed Fabuless yogurt drink compared with non-supplemented yogurt. | Adults (18-65 yr) |
| Snijder et al. (2007) ([103](#_ENREF_103)) | Population based cohort | 1896 (NR; NR) | Yogurt (low fat, skim and whole); NR | Dairy desserts, (including curds, custard) milk (low fat skim and whole) and cheese); NR | Metabolic health; Body weight; Blood pressure | Borderline significant (p<0.10) inverse associations were observed for yogurt with systolic and diastolic blood pressure, no other significant associations. | Adults (18-65 yr) and older adults  (> 65 yr) |
| Sorenson et al. (2004) ([41](#_ENREF_41)) | Cross- sectional | 1000 (approximately) (NR; NR) | Yogurt; NR | Other dairy products (as part of a questionnaire asking about intake of 15 dairy products); NR | Association with disease (heart disease, cancer, diabetes, hypertension, osteoporosis and obesity) | No negative associations were found in this study other than yogurt in men. Lacking detail of exact outcome association with yogurt. | Older adults  (> 65 yr) |
| Stritecka & Hlubik (2013) ([104](#_ENREF_104)) | Controlled trial | 200 (50; 150) | Normal yogurt (1 pot/day); 14 days | Milk product enriched with protein and fibre (1 pot/day);  14 days | Weight management | Yogurt group felt satiated for a shorter time period after consumption compared to the enriched milk group. | Adults (18-65 yr) |
| Sullivan  et al. (1989) ([105](#_ENREF_105)) | Double-blind Crossover trial | 16 (16; 16) | Standard yogurt (500 g/day); 28 days | Whey-based test yogurt prepared from commercial whey protein concentrate (500g/day);  28 days | Cardiovascular risk factors; macronutrient intake; weight management | No significant differences between test and standard yogurt for either group in mean energy, fat, or cholesterol intake, body weight, and lack of evidence for significant differences in blood lipid parameters between test and control yogurts. | Adults (18-65 yr) |
| Thomas  et al. (2011) ([106](#_ENREF_106)) | Randomized controlled trial | 35 (15 completers;  14 completers) | Yogurt, 6 oz serving, fat free (Yoplait light thick and creamy) as part of a diet with a reduced energy intake (-1,046 kJ); 16 weeks | Isoenergetic sucrose beverage, 6 oz, as part of a diet with a reduced energy intake  (-1046 kJ);  16 weeks | Weight management | Significant weight losses and reductions in total fat in both yogurt and sucrose groups. Total lean weight increased significantly in both groups. Waist circumference, sagittal diameter, and trunk fat decreased significantly in both groups. Strength significantly increased in both groups. No significant differences between groups were observed. | Adults (18-65 yr) |
| Thompson  et al. (1982) ([107](#_ENREF_107)) | Cohort study | 68 (13; Skim milk (n = 11), 2% milk (n = 11), whole milk (n = 10), sweet acidophilus milk (n = 12), buttermilk (n = 11)) | Yogurt (1 L/d) in addition to 0.25 L of 2% butterfat milk. 3-week intervention period followed by a 7-week acclimation period with varying amounts of 2% milk; 3 weeks | Skim milk, 2% milk, whole milk, sweet acidophilus milk or buttermilk (1 litre daily) in addition to 0.25 L of 2% butterfat milk. 3-week intervention period followed  a 7-week acclimation period with varying amounts of 2% milk; 3 weeks | Cardiovascular disease risk factors; Body weight | No significant increases were found in total, low-density, and high-density lipoprotemn cholesterol. A significant weight gain was seen in subjects taking yogurt. Weight gain was most marked in the yogurt and acidophilus groups; thes groups showed significant rises in triglyceride levels. | Adults (18-65 yr) |
| Trapp et al. (1993) ([108](#_ENREF_108)) | Cohort study, double-blind | 98 (NR; NR) | Live-active yogurt,  1.5% milk fat, 200 g/d vs heat-killed yogurt,  1.5% milk fat, 200 g/d. Yogurt provided by the National Yogurt Foundation; 12 months | No yogurt consumed (non-regular yogurt eaters told not to consume yogurt); 12 months | Cardiovascular risk factors;  GI discomfort; Weight management; Nutrient intake;  Allergic symptoms | There was a decrease in allergic symptoms in both the senior and young live-active yogurt groups, compared to the heat-killed and no yogurt groups. Seniors not consuming yogurt experienced an increase in both total and LDL cholesterol, while those consuming yogurt remained stable over the year. Seniors consuming yogurt experienced a significant decrease in GGT (liver enzyme associated with fat and/or alcohol metabolism). Subjects eating yogurt consumed more calcium than non-yogurt consumers. Body weight did not change over time or between groups. Trend towards less gastrointestinal distress (gas, diarrhoea, constipation) in live active yogurt consumers compared to heat killed and no yogurt. | Adults (18-65 yr) and Older adults  (> 65 yr) |
| Tsuchiya  et al. (2006) ([109](#_ENREF_109)) | Crossover trial | 32 (32; 32) | Semisolid yogurt (brand Taillefine) containing pieces of peach, eaten with a spoon (378 g as a preload, 200 kcal); Same yogurt in a drinkable homogenized form (378 g as a preload, 200kcal); one serving | A peach flavoured milk-based beverage (brand Danao) (400 mL as a preload, 200 kcal), a peach juice beverage (brand Teisseire) (400 mL as a preload, 200 kcal); one serving | Weight management | The two yogurts (semisolid and liquid) led to lower hunger and higher fullness ratings compared with the fruit drink or dairy fruit drink. There was no difference in satiety profiles between the two yogurts. Energy intakes at lunch were the same across all four conditions. | Adults (18-65 yr) |
| van der Zander et al. (2008) ([110](#_ENREF_110)) | Multicentre, randomized, double-blind, parallel, placebo- controlled trial | 271 (134; 137) | Pasteurized Yogurt;  8 weeks | Pasturized yogurt containing 200 g ELTP (Arla Foods, Copenhagen, Denmark), casein hydrolysate powder, enzymatically hydrolyzed by an Aspergillus oryzae protease; 8 weeks | Hypertension symptom relief;  Weight management | Body weight did not change significantly during the 8-wk study period in either group. A minor (non-significant) decrease in blood pressure between the ELTP and control treatments after 8 weeks; not affected by confounders in a post-hoc analysis. | Adults (18-65 yr) |
| Vandewater & Vickers (1996) ([111](#_ENREF_111)) | Crossover | 40 (40; 40) | Low protein (7% weight by volume) yogurt (close to commercial strawberry yogurt); one serving | High-protein  (15% weight by volume) strawberry yogurt (strawberry yogurt that contained whey protein isolate); one serving | Weight management | High protein yogurt produced more sensory-specific satiety (reduced hunger and reduced liking) than low protein yogurt. High protein yogurt significantly reduced hunger and significantly increased stomach fullness compared to low protein yogurt. | Adults (18-65 yr) |
| Vergnaud  et al. (2008) ([112](#_ENREF_112)) | Cross- sectional | 2267 (NR; NR) | Yogurt; NR | Non yogurt consumers; total dairy products (milk, cheese) | Weight management | Positive relations were found between yogurt consumption and weight change in normal-weight women. Inverse relationship between overweight men and yogurt consumption for weight and waist circumference changes. | Adults (18-65 yr) |
| Wang et al. (2013) ([113](#_ENREF_113)) | Cross- sectional | 6526 (3510; 3016) | Yogurt (with 9 frequency categories); NR | Non yogurt consumers; NR | Nutrient status; Cardiovascular disease risk factors; Diabetes risk factors;  Weight management | Yogurt consumers had a higher DGAI score (i.e. better diet quality) than non-consumers. Adjusted for demographic and lifestyle factors and DGAI, yogurt consumers, compared with non-consumers, had higher potassium intakes and were 47%, 55%, 48%, 38%, and 34% less likely to have inadequate intakes (based on Dietary Reference Intake) of vitamins B2 and B12, calcium, magnesium, and zinc, respectively (all p ≤ 0.001). In addition, yogurt consumption was associated with lower levels of circulating triglycerides, glucose, and lower systolic blood pressure and insulin resistance (all p < 0.05); compared with non-consumers, yogurt consumers appeared to have better metabolic profile, such as lower BMI, waist circumference, levels of triglycerides, fasting glucose and insulin, and blood pressure but higher HDL cholesterol. | Adults (18-65 yr) |
| White et al. (2009) ([114](#_ENREF_114)) | Randomized control trial | 35 (12 completers; Protein group = 12 completers; Carbohydrate group = 11 completers) | Yogurt, Yoplait light  (3 x 6 oz servings, containing Vitamin D per day) one serving consumed post exercise; 8 weeks | Low-calcium diet with protein group consumed an isocaloric product without calcium or vitamin D, post exercise; low-calcium diet with carbohydrate group consumed an isocaloric product without protein, post exercise; 8 weeks | Metabolic health;  Weight management | Yogurt group did not experience significantly different changes in body composition compared with a low-dairy diet. Yogurt group decreased % body fat as a result of training (more than the other groups), despite increased calorie and protein consumption compared to baseline. Increase in total overall strength was greater in yogurt than in protein group. No changes over time in Fat Oxidation and Resting Metabolic Rate. | Adults (18-65 yr) |
| Yae et al. (2005) ([115](#_ENREF_115)) | Randomized, double-blind, and placebo- controlled | 51 | 1 cup (150 mL) daily of low fat strawberry yogurt at breakfast; 4 weeks | 1 cup (150 mL) daily of low-fat strawberry yogurt with 3.3 g of plant stanol ester at breakfast; 4 weeks | Cardiovascular disease risk factors;  Weight management | No significant difference between the two groups for daily nutrient intake and total energy expenditure. Serum β-carotene and retinol were unchanged in both groups; and there were no significant changes in the normal yogurt group for all outcomes. | Adults (18-65 yr) |
| Zandstra  et al. (2000) ([116](#_ENREF_116)) | Crossover | 87 | Low-fat, low-carbohydrate and low energy strawberry yogurt (500 g serving). Children, young adults and elderly consumed 200, 340 and 300 g of the preload, respectively; one serving | High-fat, medium energy (71% of energy of fat;  500 g serving); High-fat, high-carbohydrate, high energy (42 of energy of fat,  53 of energy of carbohydrate;  500 g serving); No yogurt preload. Children, young adults and elderly consumed 200, 340 and  300 g of the preload, respectively; one serving | Weight management | Compared to the no preload condition, all children, young adults and elderly ate significantly less after the high-fat and high-carbohydrate yogurt. Data is not discussed for the low-fat yogurt condition; The young adults gave lower ratings after the high-fat yogurt, the high-carbohydrate yogurt and the high-fat and high-carbohydrate yogurt compared to the no-preload condition for all appetite items (all F(4950) >15.8, p<0.0001). The elderly gave only lower ratings after the consumption of the high-fat and high-carbohydrate yogurt compared to the no-preload condition for all appetite items, except for `appetite for something sweet', where the appetite ratings were lower after the consumption of the high-fat yogurt. | Older children (6-18 yr), Adults (18-65 yr) and Older adults  (> 65 yr) |
| Zemel et al. (2005) ([117](#_ENREF_117)) | Randomized control trial | 34 (19; 18 completers); 19 (16 completers) | Commercial fat-free yogurt (Yoplait Light),  3 x 6 oz servings daily as part of a 500 kcal/day-deficit diet; 12 weeks | A control diet providing a  500 kcal/day deficit, 0–1 servings of dairy products/day and 400–500 mg calcium per day, and a placebo  (3 daily servings of a sugar-free, calcium-free, prepackaged flavoured gelatin dessert containing 10 kcal/serving); 12 weeks | Weight management | Weight and fat loss was markedly increased in the yogurt group, lean tissue loss was reduced by 31%. Trunk fat loss, reduction in waist circumference and the fraction of fat lost from the trunk was higher on the yogurt diet vs control; Participants on the yogurt diet exhibited a significant increase in circulating glycerol, indicating an increase in lipolysis in individuals on this diet, while there was no significant change in circulating glycerol in participants on the control diet; patients on the yogurt diet exhibited a significant reduction in diastolic pressure (p=0.01), consistent with the modest blood pressure lowering. | Adults (18-65 yr) |

CCK, cholecystokinin; CLA; conjugated linoleic acid; DGAI, Dietary Guidelines Adherence Index; ELTP, enzymatically hydrolyzed lactotripeptides; GGT, gamma-glutamyl transferase, GLP-1, glucagon-like peptide-1; HgPE, H. gordonii purified extract; NR, not reported; PUFA, polyunsaturated fatty acids.

**Supplementary Table F: Summary table for yogurt studies assessing metabolic health outcomes**

| **Reference** | **Study**  **design** | **Total sample size: (Intervention; Comparator)** | **Intervention details (duration)** | **Comparator details**  **(duration)** | **Eligible outcomes** | **Comments** | **Age group** |
| --- | --- | --- | --- | --- | --- | --- | --- |
| Beydoun  et al. (2008) ([48](#_ENREF_48)) | Cross-sectional | 17061, 4519 with complete datasets (NR; NR) | Yogurt; NR | Dairy products (including cheese, low-fat milk, whole milk, fluid milk, skim milk) and nutrients (including phosphorous); NR | Obesity, central obesity, and metabolic syndrome | Significant inverse association between intake of yogurt and obesity, central obesity and metabolic disorders. Results also analysed by ethnic group, sex and age. | Adults (18-65 yr) |
| Bonet Serra et al. (2008) ([50](#_ENREF_50)) | Double-blind case-controlled | 39 (NR; NR) | Yogurt (liquid, 200 g, supplemented with 3 g of CLA); 16 weeks | Placebo;  16 weeks | Metabolic disorder risk factors; Cardiovascular risk factors; Weight management | After 16 weeks, CLA-supplemented yogurt decreased the plasma glucose levels and insulin resistance index (HOMA), independently of weight changes and BMI changes, compared to placebo. No significant differences were observed in weight, height, BMI, total cholesterol and in HDL and adiponectin between the placebo group and the CLA-supplemented yogurt group at any time point. | Adults (18-65 yr) |
| Kim (2013) ([118](#_ENREF_118)) | Cross- sectional | 4862 (NR; NR) | Yogurt consumption (all low-fat, skim and whole yogurt) | Dairy food; milk consumption (included low-fat, skim and whole milk) | Metabolic syndrome risk; Cardiovascular disease risk factors | Prevalence of the metabolic syndrome was significantly lower for high yogurt consumers. A high yogurt intake was associated with a reduced risk of low HDL cholesterol and the metabolic syndrome after adjusting for potential risk factors. | Adults (18-65 yr) |
| Snijder  et al. (2007) ([103](#_ENREF_103)) | Population based cohort | 1896 (NR; NR) | Yogurt (low fat, skim and whole); NR | Dairy desserts, (including curds, custard) milk (low-fat skim and whole) and cheese); NR | Metabolic health;  Body weight; Blood pressure | Borderline significant (p < 0.10) inverse associations were observed for yogurt with systolic and diastolic blood pressure, no other significant associations. | Adults (18-65 yr) and Older adults  (> 65 yr) |
| Troy et al. (2010) ([119](#_ENREF_119)) | Cross- sectional | 3104 (NR; NR) | Yogurt intake; NR | Dairy intake vs milk intake; NR | Metabolic syndrome | After adjustment for age, sex, smoking, total energy and alcohol intakes, yogurt intake was inversely associated with metabolic syndrome, but the effect was no longer significant after adjustment for other aspects of a healthy dietary pattern (whole grain, fruit and vegetable intakes). Inverse association was observed between yogurt intakes and prevalence of hypertension, this association remained significant after adjustment for aspects of a healthy dietary pattern and supplemental calcium. | Adults (18-65 yr) |
| White et al. (2009) ([114](#_ENREF_114)) | Randomized Control Trial | 35 (12 completers; Protein group = 12 completers; Carbohydrate group = 11 completers) | Yogurt, Yoplait light (3 x 6 oz servings, containing Vitamin D per day) one serving consumed post exercise;  8 weeks | Low-calcium diet with protein group consumed an isocaloric product without calcium or vitamin D, post exercise; Low-calcium diet with carbohydrate group consumed an isocaloric product without protein, post exercise; 8 weeks | Metabolic health; Weight management | Yogurt group did not experience significantly different changes in body composition compared with a low-dairy diet. Yogurt group decreased % body fat as a result of training (more than the other groups), despite increased calorie and protein consumption compared to baseline. Increase in total overall strength was greater in yogurt than in protein group. No changes over time in Fat Oxidation and Resting Metabolic Rate. | Adults (18-65 yr) |

CLA; conjugated linoleic acid; HOMA, homeostasis model assessment of insulin resistance; NR, not reported.

**Supplementary Table G: Summary table for yogurt studies assessing cardiovascular health outcomes**

| **Reference** | **Study**  **design** | **Total sample size: (Intervention; Comparator)** | **Intervention details (duration)** | **Comparator details**  **(duration)** | **Eligible outcomes** | **Comments** | **Age group** |
| --- | --- | --- | --- | --- | --- | --- | --- |
| Amir Shaghaghi (2012) ([120](#_ENREF_120)) | Double-blind, randomized, crossover | 47 (47; 47; 47) | Yogurt (no supplement);  29 days | WD-PS-enriched yogurt (2g/d) vs PS-esters-enriched yogurt (2g/d); duration= 29 days | Cardiovascular disease risk factors | WD-PS and PS-esters reduced (p< 0.001) endpoint total and LDL cholesterol levels compared with control. | Adults (18-65 yr) |
| Azadbakht et al. (2013) ([45](#_ENREF_45)) | Randomized controlled clinical trial | 68 (34;34) | Yogurt as a preload before lunch and dinner, 0.5-1 serving, with 2 servings of vegetable salad and 1 glass of water; as part of a calorie restricted diet (-200 to -500kcal/day); 3 months. | Yogurt with lunch and dinner, 0.5-1 serving and 2 servings of vegetable salad; as part of a calorie restricted diet (-200 to -500kcal/day); 3 months. | Cardiovascular disease risk; weight management | Consuming the preload (yogurt, vegetables and water) compared to with the meal (yogurt and vegetables) leads to a lowering of triglyceride fasting blood sugar, total cholesterol and LDL-C. | Adults (18-65 yr) |
| Bazzarre et al. (1983) ([46](#_ENREF_46)) | Crossover trial | 30, 21 completers (30;30) | Yogurt, 3 x 8oz servings of Dannon low fat yogurt at morning, noon and evening) in addition to normal diet; 1 week. | Normal diet; Normal diet with 3x 250mg calcium supplement pills at morning, noon and evening meals; 1 week. | Cardiovascular risk factors; Weight management | Mean total cholesterol decreased significantly (P < 0.001) for females following yogurt supplementation. Total cholesterol ratios for females were significantly higher (P < 0.0001) following yogurt than baseline values. | NR-Likely to be Adults (18-65 yr) |
| Bonet Serra et al. (2008) ([50](#_ENREF_50)) | Double blind case-controlled | 39 (NR; NR) | Yogurt (liquid, 200g, supplemented with 3 g of CLA); 16 weeks. | Placebo; 16 weeks. | Metabolic disorder risk factors; Cardiovascular risk factors (blood lipids); Weight management | No significant differences were observed in total cholesterol and in HDL and adiponectin between the placebo group and the CLA-supplemented yogurt group at any time point. | Adults (18-65 yr) |
| Bonthuis et al. (2010) ([121](#_ENREF_121)) | Prospective cohort | 1529 (NR; NR) | Yogurt; NR. | Other various foods, identified by food frequency questionnaire; NR. | Cancer mortality; cardiovascular mortality; all-cause mortality | Yogurt was not associated with cardiovascular mortality. | Adults (18-65 yr) and Older adults (over 65) |
| Buyuktuncer et al. (2013) ([7](#_ENREF_7)) | Randomised, double-blind, placebo-controlled study design | 70 (35; 35) | 115 g/day spoonable low-fat yogurt (Yıldız Holding), in addition to normal diet; 4 weeks. | 115 g/day spoonable low-fat yogurt with 1.9 g plant stanols as esters (Yıldız Holding), in addition to normal diet; 4 weeks. | Cardiovascular risk factors; Weight management | Serum total cholesterol, LDL cholesterol, and non-HDL cholesterol concentrations were reduced significantly from baseline in the plant stanol yogurt group compared to the non-supplemented yogurt group. | Adults (18-65 yr) |
| Chien et al. (2004) ([57](#_ENREF_57)) | Cohort | 70 (NR; NR) | Yogurt commercially packaged meals; total energy intake was 1200 kcal/day; 6 weeks. | Traditional low calorie diet; total energy intake was 1200 kcal/day; 6 weeks. | Weight management; Cardiovascular risk factors | After 6 weeks, serum total cholesterol, triglycerides, LDL, GOT, and GPT concentrations had significantly decreased in yogurt group. | Adults (18-65 yr) |
| Clegg et al. (2011) ([58](#_ENREF_58)) | Randomised single-blind crossover trial | 11 (11; 11; 11) | Low fat yogurt, 260g once daily, in addition to a normal diet; 3 days. | High fat yogurt (260g yogurt with 90g of sunflower oil), once daily, in addition to a normal diet; No additional yogurt; 3 days. | Weight management; Cardiovascular risk factors; GI transit time | The high fat yogurt reduced triglycerides, total cholesterol and low-density lipoprotein cholesterol, and increased high-density lipoprotein cholesterol. | Adults (18-65 yr) |
| Cramer et al. (1989) ([18](#_ENREF_18)) | Case-control | 474 (NR; NR) | Yogurt; NR. | Other dairy products including Whole milk; Skimmed milk; Ice milk; Ice Cream; Cottage cheese; Cream Cheese; NR. | Ovarian cancer risk; Cardiovascular risk factors | Excluding the anti-hypertensive medication users at follow-up strengthened the longitudinal association between yogurt intake and annualized SBP change; Yogurt intake was not related to DBP change over time. | Adults (18-65 yr) and Older adults (over 65) |
| Dawczynski & Jahreis (2009) ([19](#_ENREF_19)) | Cohort | NR | Low fat (less enriched) yogurt; 125g; 5 or 10 weeks. | 08 g n-3-LC-PUFA (Omega 3)/125 g yogurt; 5 or 10 weeks. | Cardiovascular disease risk | No favourable changes in HDL-cholesterol and in the LDL/HDL quotient in the low fat yogurt group. Yogurt group had a trend for decrease TAG | Adults (18-65 yr) and Older adults (over 65) |
| Dawczynski et al. (2013a) ([122](#_ENREF_122)) | Placebo-controlled, randomized double-blind parallel | 53 (14; 33 (17 in group 2 and 16 in group 3)) | Commercial fruit yogurt (125 g, 3.5% w/w fat) daily; 10 weeks. | Group 2= 125 g yogurt with approx. 3.5% fat (approx. 2.5% fish oil and 1% milk fat), 0.8g of n-3 PUFA vs Group 3= 200 g yogurt with approx. 5.5% fat (approx. 5% fish oil and 0.5% milk fat), 3 g of n-3 PUFA; 10 weeks. | Cardiovascular disease risk factors | No significant changes where observed in the commercial fruit yogurt group from baseline to week 10. | Adults (18-65 yr) and Older adults (over 65) |
| Dawczynski et al. (2013b) ([59](#_ENREF_59)) | Randomized, placebo-controlled, double-blind, parallel | 47 (14; Group 2= 17; Group 3= 16) | Commercial fruit yogurt (125 g, 3.5% w/w fat); 10 weeks. | Group 2= 125 g yogurt with approx. 3.5% fat (approx. 2.5% fish oil and 1% milk fat), 0.8g of n-3 PUFA vs Group 3= 200 g yogurt with approx. 5.5% fat (approx. 5% fish oil and 0.5% milk fat), 3 g of n-3 PUFA; 10 weeks. | Cardiovascular risk factors; Micronutrient intake | No significant changes in cardiovascular risk factors or blood cholesterol levels were observed in the non-supplemented yogurt group when comparing wk. 0 to wk. 10. | Adults (18-65 yr) |
| Freudenheim et al. (1991) ([123](#_ENREF_123)) | Cross-sectional | 1790 (NR; NR) | Yogurt as a form of calcium intake; NR. | Various other foods that provide calcium (assessed by questionnaire); NR. | Cardiovascular risk factors | Diastolic blood pressure was inversely associated with calcium from yogurt for white females, same non-significant trend for black females, no trend for males. | Adults (18-65 yr) and Older adults (over 65) |
| Ganji & Kafai (2004a) ([124](#_ENREF_124)) | Cross- sectional | 6615 (NR; NR) | Yogurt; NR. | Other various foods, including milk and cheese (identified by food frequency questionnaire); NR. | Cardiovascular disease risk factor | Sex and age adjusted tHcy concentrations were 25% higher in subjects who never consumed yogurt than those who consumed yogurt >30 times/mo (p=0.001), | Adults (18-65 yr) |
| Ganji & Kafai (2004b) ([125](#_ENREF_125)) | Cross- sectional | 5996 (NR; NR) | Yogurt (including frozen yogurt); NR. | Milk (milk to drink or on cereal); cheeses (all types) and various other foods and food groups; NR. | Cardiovascular disease risk factor | tHcy concentrations were 6.4% higher in subjects who never consumed yogurt than in those who consumed yogurt greater than 15 times/month. | Older children (6-18); Adults (18-65) and Older Adults (>65) |
| Goldbohm et al. (2011) ([16](#_ENREF_16)) | Case-cohort | 20782 (NR; NR) | Yogurt (full fat (3.6% fat) and skim yogurt (0.1% fat)) | Milk, buttermilk, quark and dishes were dairy products were used.  3 items of cheese and 3 of butter. These were further specified by fat content (full, low or skim) | Total (all-cause) and cardiovascular mortality (Ischemic heart disease and stroke) | In men, yogurt was inversely associated with mortality. Exclusion of the first 2 y of follow-up or prevalent cases with cardiovascular disease did not change these estimates. In women, no association between yogurt and mortality was observed. | NR-Likely to be Adults (18-65 yr) and Older adults (over 65) |
| Gouni-Berthold et al. (2012) ([126](#_ENREF_126)) | Multi-centre, double-blind, randomised, placebo controlled trial with two parallel treatment groups | 180 (92; 88) | Low fat spoonable yogurt (Technologie Biolactis) supplemented with small amounts of hydrolysed gelatine in 150g servings. One serving taken twice daily at morning and evening mealtimes; 3 months. | Low-fat spoonable yogurt (WheygurtTM, Technologie Biolactis), supplemented with 7g of malleable protein matrix (MPM) per serving, in 150g servings. One serving taken twice daily at morning and evening mealtimes; 3 months. | Cardiovascular risk factors | MPM- supplemented yogurt resulted in a significantly larger reduction of TAG concentrations, and significantly lower blood pressure in comparison to non-supplemented yogurt. | Adults (18-65 yr) |
| Hepner et al. (1979) ([127](#_ENREF_127)) | Cohort | 56 (Unpasteurized (11); Pasteurized (10); Milk with 2% butterfat (10); no supplementation (5)) | Pasteurised yogurt or unpasteurised yogurt (Dannon Milk Products; both contain live culture of live culture of Lactobacillus bulgaricus and Streptococcus thermophilus). Three 240ml cups/day; either constant supplementation or supplementation in first and last 4 weeks of a 12 week study. | Milk with 2% butterfat (720ml/day); or no supplementation (n=5); either constant supplementation or supplementation in first and last 4 weeks of a 12 week study. | Cardiovascular disease risk factors | Serum cholesterol was significantly reduced by 5 to 10% after 1 week of supplementation with either nonpasteurized or pasteurized yogurt. Note: this was two relevant studies (different supplementation regimes) reported in one paper; The data indicate that yogurt supplementation of diet causes a significant reduction of serum cholesterol, without affecting serum triglycerides or weight. | Adults (18-65 yr) |
| Iso et al. (1999) ([128](#_ENREF_128)) | Population based cohort | 85764 (NR; NR) | Yogurt consumption; NR. | Other items, including dairy, as part of a food frequency questionnaire; duration NR. | Stroke risk | Inverse relation between yogurt intake and the age- and smoking-adjusted risk of ischemic stroke. | Adults (18-65 yr) |
| Ivey et al. (2011) ([129](#_ENREF_129)) | Cross sectional | 1080 (NR; NR) | Yogurt; NR. | Milk vs cheese; NR. | Cardiovascular disease risk factors | Yogurt consumption was negatively associated with CCA-IMT (significant). Participants who consumed greater than 100 g yogurt/day had a significantly lower CCA-IMT than did participants with lower consumption (significant). This relation remained significant after adjustment for baseline, dietary, and lifestyle risk factors; High yogurt consumption was associated with lower CCA-IMTand higher HDL cholesterol. | Older Adults (>65) |
| Jaspers et al. (1984) ([130](#_ENREF_130)) | Cross-over trial | 10 (10; 10) | Non-fat Unpasteurized yogurts (2 types) with either of 2 commercial starter strains of L. bulgaricus and S. thermophilius, CHR Hansen Laboratories; 14 days for each yogurt. | Non-fat Unpasteurized yogurts with patented starter strains of L. bulgaricus and S. thermophilius, supplied by Dr K.M.Shanni, Univ. of Nebraska; 21 days. | Cardiovascular disease risk | Daily yogurt significantly reduced fasting total serum cholesterol by 10-12% in human adult male on some days, but serum cholesterol returned towards control values with continued yogurt consumption. No significant effect on serum triglycerides and the proportions of serum lipoproteins. | Adults (18-65 yr) and older adults (>65yr) |
| Javed et al. (2013) ([131](#_ENREF_131)) | Controlled trial | 30 (Group1=10; Group2= 10; 10) | Group1: 300g/day of branded yogurt; Group2 300g/day of conventional yogurt (dahi); 60 days. | No yogurt; 60 days. | Cardiovascular disease risk factors | Yogurt (branded and conventional) consumption for 60 days significantly reduced total cholesterol and LDL cholesterol and significantly elevated HDL cholesterol in comparison with control group (no yogurt). For the reductions, the effect was more profound in branded yogurt as compared to conventional yogurt. | Adults (18-65 yr) |
| Khandelwal et al. (2009) ([132](#_ENREF_132)) | Double-blind, randomised, controlled, parallel trial with a 2 x 2 factorial design | 178 (46; 40; 45; 47) | 100 ml portion of yogurt drink with control (non-active safflower seed oil) capsules; 4 weeks. | 100 ml portion of yogurt drink with fish oil capsules; 100 ml portion of plant sterol-enriched yogurt drink with fish oil capsules;100 ml portion of plant sterol-enriched yogurt drink with control (non-active safflower oil) capsules; 4 weeks. | Cardiovascular risk factors | Significant reduction in total cholesterol and LDL cholesterol in plant sterol enriched vs non-supplemented yogurt. Plasma TAG and the TC:HDL-C ratio was decreased in the plant sterol yogurt treatment compared to the non-supplemented yogurt. No significant effect between enriched and non-supplemented yogurt for apolipoproteins. | Adults (18-65 yr) |
| Kim (2013) ([118](#_ENREF_118)) | Cross- sectional | 4862 (NR; NR) | Yogurt consumption (all low-fat, skim and whole yogurt) | Dairy food; milk consumption (included low-fat, skim and whole milk) | Metabolic syndrome risk; Cardiovascular disease risk factors | A high yogurt intake was associated with a reduced risk of low HDL cholesterol after adjusting for potential risk factors. | Adults (18-65 yr) |
| Korpela et al. (2006) ([133](#_ENREF_133)) | Parallel, double-blind controlled | 50 (25; 25) | Low-fat yogurt, flavoured, 150g/day; 6 weeks. | Low-fat yogurt enriched with plant sterol (less than 2g), flavoured, 150g/day;  6 weeks. | Cardiovascular risk factors | No significant difference between plant sterol yogurt group and non-supplemented yogurt group in total cholesterol change over the study. LDL cholesterol reduced significantly more during the study in the plant sterol yogurt group than the non-supplemented yogurt group. | Adults (18-65 yr) |
| Larsson et al. (2009) ([134](#_ENREF_134)) | Population based cohort study (randomized, double-blind, placebocontrolled, primary prevention trial) | 26556 (NR; NR) | Yogurt; NR. | Other dairy products, including total dairy, low-fat milk, sour milk, cheese, ice cream, butter, whole milk (identified by food frequency questionnaire); NR. | Stroke risk | Positive association between yogurt intake and subarachnoid haemorrhage. | Adults (18-65 yr) and older adults (>65yr) |
| Masala et al. (2008) ([135](#_ENREF_135)) | Cross sectional | 7601 (NR; NR) | Yogurt consumption; NR. | Various other food stuffs (assessed by questionnaire); NR. | Cardiovascular disease risk | Inverse association of yogurt with systolic blood pressure. | Adults (18-65 yr) |
| Massey (1984) ([78](#_ENREF_78)) | Crossover trial | 30 (30; 30) | Plain low fat yogurt (2 x 240ml cartons, daily); 4 weeks. | No yogurt;  4 weeks. | Cardiovascular disease risk factors; Nutrient intake | Although some serum lipid means differed significantly among some sampling points, there was no effect on total cholesterol, total triglycerides, high density lipoprotein cholesterol, or distribution of electrophoretic lipoprotein fractions. | Adults (18-65 yr) |
| McNamara et al. (1989) ([79](#_ENREF_79)) | Prospective cross-over trial | 18 (18; 18) | Low-fat yogurt (16 oz./day) as part of a low-fat, low-cholesterol baseline diet; 4 weeks. | Non-fermented dairy product (16 oz. low-fat milk plus 10% milk solids) as part of a low-fat, low-cholesterol baseline diet; duration= 4 weeks. No yogurt supplementation to the low fat, low cholesterol diet; 3 weeks. | Cardiovascular risk factors; weight management; macronutrient intake | Plasma total, LDL- and HDL-cholesterol were unaffected by either the yogurt of low-fat milk concentrate. | Adults (18-65) |
| Meneton et al. (2013) ([80](#_ENREF_80)) | Cross- sectional | 6167 (NR; NR) | Yogurt; NR. | Various other foodstuffs (identified by food frequency questionnaire); NR. | Cardiovascular disease risk; weight management | Overweight patients eat more yogurts than non-overweight; hypertensive patients eat less yogurt than patients without hypertension. No significant difference in yogurt consumption between patients with and without dyslipidmia. | Adults (18-65 yr) |
| Mensink et al. (2002) ([82](#_ENREF_82)) | Double blind, placebo-controlled, parallel design | 60 (30; 30) | Low-fat yogurt (0.7% fat); 3x 150ml cups daily, with meals; 4 weeks | Low-fat yogurt (0.7% fat, 1g of plant stanol per cup); 3x 150ml cups daily, with meals; 4 weeks. | Cardiovascular risk factors; weight management | During the experimental period, serum total cholesterol and LDL cholesterol concentrations increased slightly for the non-supplemented yogurt group. HDL cholesterol and triglyceride concentrations did not change and total to HDL cholesterol ratio hardly changed in the non-supplemented yogurt group. | Adults (18-65 yr) |
| Nakamura et al. (2002) ([136](#_ENREF_136)) | Crossover trial | 89 (89; 89) | Normal yogurt (200ml daily); 4 weeks. | Yogurt supplemented with insoluble dietary fibres (200ml daily); 4 weeks. | Cardiovascular disease risk factors; Diabetes risk factors | Total cholesterol of Brewer’s Yeast yogurt group was significantly lower than for the Normal yogurt group (but HDL and LDL levels were not significantly different). For the Normal yogurt group, Total cholesterol, HDL cholesterol, and free fatty acids were significantly lower and fasting blood sugar was significantly higher than baseline for cross-over group A. For cross-over group B, free fatty acid was significantly lower and fasting blood sugar was significantly higher than baseline, no significant difference for cholesterol; For all samples total cholesterol, HDL and LDL cholesterol, and fatty acid levels decreased significantly for both test and control groups. There were no significant differences between groups. | Adults (18-65 yr) |
| Niittynen et al. (2008) ([137](#_ENREF_137)) | Study I was a randomized double-blind, cross-over trial. Study II was a randomized, double-blind, parallel-group | Study 1= 15 (15; 15); Study 2= 26 (14;12) | Study 1= Yogurt (non-supplemented); 4 weeks. Study 2= Yogurts (non-supplemented) two daily; 8 weeks. | Study 1= Yogurt containing 1 g plant sterols; 4 weeks. Study 2= Yogurts, two daily (2 g/day plant sterols); 8 weeks. | Cardiovascular risk factors | Across both studies, a low-fat yogurt enriched with 1-2 g/day plant sterols reduced serum cholesterol levels in moderately hypercholesterolaemic subjects, compared to non-supplemented yogurt. Campesterol and sitosterol serum levels increased, but their concentration remained in the range of normal values. | Adults (18-65 yr) |
| Oosthuizen et al. (1998) ([88](#_ENREF_88)) | Double blind randomised | 21 (7; 7; 7) | Frozen yogurt, 175 g/d as part of a high-fibre, low-fat diet; 4 weeks. | Frozen yogurt, 175 g/d with 20 g soya bean lecithin as part of a high-fibre, low-fat diet; Frozen yogurt, 175 g/d with 17 g sunflower oil as part of a high-fibre, low-fat diet; 4 weeks. | Cardiovascular risk factors; Weight management | Significant decrease from baseline levels in HDL cholesterol and LDL/HDL cholesterol in the non-supplemented yogurt group; Plasma palmitic (C16:0) and palmitoleic acid (C16:1) levels increased significantly in the yogurt group. | Adults (18-65 yr) |
| Radler et al. (2011) ([138](#_ENREF_138)) | Prospective placebo-controlled double-blind | 42 (22; 20) | Yogurt, low fat, ingested twice daily; 12 weeks. | Yogurt, low fat, enriched with a combination of low-dose PUFAs, polyphenols and L-carnitine (PPC), ingested twice daily; 12 weeks. | Cardiovascular risk factors | In non-supplemented yogurt group, plasma lipid levels did not change. | NR, Likely to be Adults (18-65 yr) and older adults (>65 yr) |
| Recio et al. (2011) ([139](#_ENREF_139)) | Case control | 131 (NR; NR) | Yogurt, liquid, 150 ml once daily; 6 weeks. | Yogurt, liquid, enriched active casein hydrolysate, 150 ml once daily; 6 weeks. | Hypertension symptoms | No significant changes in blood pressure were detected in both the placebo and the normotensive groups, in the active ingredient group, systolic blood pressure was reduced for hypertensive patients. | Adults (18-65 yr) and older adults (>65 yr) |
| Rossouw et al. (1981) ([140](#_ENREF_140)) | Randomised controlled trial | 32 (11; Skimmed milk=11; Full cream milk=10) | Yogurt 1.8% fat (2 litres, in aliquots of 500ml across the day) in addition to normal diet; 3 weeks. | Skim milk 0.2% fat; full cream milk 3.3% fat (2 litres, in aliquots of 500ml across the day); 3 weeks. | Cardiovascular disease risk factors | Serum total cholesterol and low-density Iipoprotein cholesterol rose for the first 2 wk. on yogurt. These changes correlated with dietary fat and cholesterol intakes. Total cholesterol returned to base-line values during the 3rd wk. on yogurt. High-density lipoprotein cholesterol and percentage high-density lipoprotein/total cholesterol rose transiently in all three groups; For Serum TG In the yogurt group a transient significant rise was observed after I wk. (p < 0.01). | Older Children (6 - 18) |
| Rudkowska et al. (2007) ([141](#_ENREF_141)) | Randomised cross over trial | 26 (26; 26; 26) | Low fat yogurt, duration= NR | Yogurt (1.6 g/d of free Plant sterol, given in low-fat yogurt) consumed as an afternoon snack; Yogurt (1.6 g/d of Plant free sterol, given in low-fat yogurt) consumed prior to supper; NR. | Cardiovascular risk factors | Low density lipoprotein tended to be lower at the end of standard yogurt phase compared to beginning; endpoint total cholesterol was decreased after plant stanol snack compared to standard yogurt. | NR-Likely to be Adults (18-65 yr) |
| Rudkowska et al. (2008) ([142](#_ENREF_142)) | Single blind, randomized counter balanced, crossover, clinical trial | 30 (30; 30; 30) | Yogurt, consumed either with supper or as a snack; 30 days. | Yogurt (1.6 g/d of free Plant sterol, given in low-fat yogurt) consumed as an afternoon snack; Yogurt (1.6 g/d of Plant free sterol, given in low-fat yogurt) consumed prior to supper; 30 days. | Cardiovascular risk factors | Total cholesterol, LDL-cholesterol and HDL cholesterol, but not Triglycerides, all decreased from baseline at 30 days in the non-enriched yogurt group. The enriched yogurt snack and enriched yogurt with meal interventions resulted in lower total cholesterol, LDL-C and HDL-C compared to non-enriched yogurt. | Adults (18-65 yr) |
| Sadrzadeh-Yeganeh et al. (2010) ([143](#_ENREF_143)) | Randomised clinical trial (triple blinded) | 60 (30; 30) | Conventional yogurt (300g daily); 6 weeks. | No yogurt; 6 weeks. | Cardiovascular disease risk factors; Weight management | Results showed no significant difference in lipid profile, anthropometric measurements, or nutrient intake within any group throughout the study. For the total: HDL-cholesterol ratio the post hoc test showed that the decrease was significant for the conventional yogurt group compared with the control group; decrease in total cholesterol was significant for the conventional yogurt group compared with the control group. | Adults (18-65 yr) |
| Snijder et al. (2007) ([103](#_ENREF_103)) | Population based cohort | 1896 (NR; NR) | Yogurt (low fat, skim and whole); NR. | Dairy desserts, (including curds, custard) milk (low fat skim and whole) and cheese); NR. | Metabolic health; body weight; Blood pressure | Borderline significant (P < 0.10) inverse associations were observed for yogurt with systolic and diastolic blood pressure, no other significant associations. | Adults (18-65 yr) and older adults (>65 yr) |
| Soedamah-Muthu et al. (2012) ([26](#_ENREF_26)) | Cohort | 4526 (NR; NR) | Yogurt; NR. | Various food stuffs identified by a Food Frequency Questionnaire; NR. | Diabetes (type 2) risk; Coronary heart disease risk; all-cause mortality | Intakes of yogurt were not associated with incident diabetes (type 2) or coronary heart disease. | Adults (18-65 yr) |
| Sorenson et al. (2004) ([41](#_ENREF_41)) | Cross sectional | 1000 (Approximately) (NR; NR) | Yogurt; NR. | Other dairy products (as part of a questionnaire asking about intake of 15 dairy products); NR. | Association with disease (heart disease, cancer, diabetes, hypertension, osteoporosis and obesity) | No negative associations were found in this study other than yogurt in men. Lacking detail of exact outcome association with yogurt. | Older adults (>65yr) |
| Steffen & Jacobs (2003) ([144](#_ENREF_144)) | Population based cohort | 2778 (NR; NR) | Yogurt (consumers of yogurt); NR. | No yogurt (non-consumers of yogurt); NR. | Cardiovascular risk factors | Triglyceride levels decreased in Yogurt consumers compared to non-consumers. | Adults (18-65 yr) and older adults (>65yr) |
| Steffen et al. (2005) ([145](#_ENREF_145)) | Multicentre, population-based, prospective cohort | 4304 (NR; NR) | Yogurt; NR. | Various other food stuffs identified by questionnaire; NR. | Hypertension association | No association between yogurt and Elevated Blood Pressure. | Adults (18-65 yr) |
| Sullivan et al. (1989) ([105](#_ENREF_105)) | Double blind Cross over trial | 16 (16; 16) | Standard yogurt (500g/day); 28 days. | Whey-based test yogurt prepared from commercial whey protein concentrate (500g/day); 28 days. | Cardiovascular risk factors; macronutrient intake; weight management | Lack of evidence for significant differences in blood lipid parameters between standard and whey based yogurts. | Adults (18-65 yr) |
| Tavani et al. (2002) ([146](#_ENREF_146)) | Case control | 985 (NR; NR) | Yogurt; NR. | Other various foods and nutrients, including milk; cheese; calcium (identified as part of a food frequency questionnaire); NR. | Acute myocardial infarction (AMI) risk | A significant inverse trend in risk for an intake of at least 7 cups of yogurt/ week. | Adults (18-65 yr) and Older adults (over 65) |
| Thompson et al. (1982) ([107](#_ENREF_107)) | Cohort | 68 (13; Skim milk (n = 11), 2% milk (n = 11), whole milk (n = 10), sweet acidophilus milk (n = 12), buttermilk (n = 11)) | Yogurt (1 litre daily) in addition to 0.25l of 2% butterfat milk. Three week intervention period followed a 7 week acclimation period with varying amounts of 2% milk; 3 weeks. | Skim milk, 2% milk, whole milk, sweet acidophilus milk or buttermilk (1 litre daily) in addition to 0.25l of 2% butterfat milk. Three week intervention period followed a 7 week acclimation period with varying amounts of 2% milk; 3 weeks. | Cardiovascular disease risk factors; Body weight | Despite increases in caloric intakes on all supplements, no significant increases were found in total, low-density, and high-density lipoprotein cholesterol. The yogurt and acidophilus groups were the only two groups showing significant rises in triglyceride levels. | Adults (18-65 yr) |
| Trapp et al. (1993) ([108](#_ENREF_108)) | Cohort;double blinded | 98 (NR; NR) | Live-active yogurt, 1.5% milk fat, 200g daily vs Heat-killed yogurt, 1.5% milk fat, 200g daily. Yogurt provided by the National Yogurt Foundation; 12 months. | No yogurt consumed (non-regular yogurt eaters told not to consume yogurt); 12 months. | Cardiovascular risk factors; GI discomfort; weight management; nutrient intake; Allergic symptoms | Seniors not consuming yogurt experienced an increase in both total and LDL cholesterol, while those consuming yogurt remained stable over the year. | Adults (18-65 yr) and older adults (>65yr) |
| Trautwein et al. (2004) ([147](#_ENREF_147)) | Randomised double-blind cross-over | 40 (40; 40) | Placebo; 3 weeks. | Plant sterol enriched-yogurt (1.8 g/d); 150g portion, twice daily vs Plant stanol enriched-yogurt (1.7 g/d); 150g portion, twice daily; 3 weeks. | Cardiovascular risk factors | Both the enriched yogurts reduced LDL-cholesterol significantly compared to placebo; there was no effect on HDL-cholesterol and triglyceride concentrations. | Adults (18-65 yr) |
| Trautwein et al. (2005) ([148](#_ENREF_148)) | Double blind, parallel-arm | 184 (NR; NR) | Placebo; 4 weeks. | PS-ester enriched yogurt single-shot drink (100 g; 3.2 g PS/d) 0.1% dairy fat consumed with a meal vs PS-ester enriched yogurt single-shot drink (100 g; 2.8 g PS/d) 1.5% dairy fat consumed with a meal vs PS-ester enriched yogurt single-shot drink (100 g) 0.1% dairy fat consumed without a meal vs PS-ester enriched yogurt single-shot drink (100 g) 1.5% dairy fat consumed without a meal; 4 weeks. | Cardiovascular risk factors | LDL-C was significantly lowered when the single-shot drink was taken with the meal independent of its fat content as compared to placebo. When consumed on an empty stomach, LDL-C was significantly decreased as compared to placebo. | Adults (18-65 yr) and older adults (>65yr) |
| van der Zander et al. (2008) ([110](#_ENREF_110)) | Multicentre, randomized, double-blind, parallel, placebocontrolled trial | 271 (134; 137) | Pasteurized Yogurt; 8 weeks. | Pasteurised yogurt containing 200 g ELTP (Arla Foods, Copenhagen, Denmark), casein hydrolysate powder, enzymatically hydrolysed by an Aspergillus oryzae protease; 8 weeks. | Hypertension symptom relief; Weight management | A minor (non-significant) decrease in blood pressure between the ELTP and control treatments after 8 weeks; not affected by confounders in a post-hoc analysis. | Adults (18-65 yr) |
| Wang et al. (2008) ([149](#_ENREF_149)) | Prospective cohort | 28886 (NR; NR) | Yogurt consumption; NR. | Various other food stuffs identified by questionnaire including total dairy; Skim milk; Cottage Cheese; NR. | Hypertension risk | Statistically significant reduction in risk for yogurt (and skim milk). Multivariate adjustment substantially attenuated the inverse association for yogurt only. | Adults (18-65 yr) |
| Wang et al. (2012) ([150](#_ENREF_150)) | Population based cohort | 2197 (NR; NR) | Yogurt consumer group (one serving per month); high intake yogurt group (people who consumed >2% of total calories from yogurt); NR. | Other various foods (identified by food frequency questionnaire); NR. | Cardiovascular disease risk factors | High-intake yogurt group had lower risk of incident hypertension and smaller annualized elevation of systolic blood pressure than non-consumers. All analyses were adjusted for demographic and lifestyle factors (including diet quality) and trends remained significant when anti-hypertensive medication users were removed, and controlling for BMI and BMI change. Yogurt intake was not related to diastolic blood pressure change over time. | Adults (18-65 yr) and Older adults (over 65) |
| Wang et al. (2013) ([113](#_ENREF_113)) | Cross sectional | 6526 (3510; 3016) | Yogurt (with 9 frequency categories); NR. | Non yogurt consumers; NR. | Nutrient status; Cardiovascular disease risk factors; Diabetes risk factors; Weight management | Yogurt consumption was associated with lower levels of circulating triglycerides, glucose, and lower systolic blood pressure and insulin resistance. Compared with nonconsumers, yogurt consumers appeared to have lower levels of triglycerides but higher HDL cholesterol. | Adults (18-65 yr) |
| Yae et al. (2005) ([115](#_ENREF_115)) | Randomized, double blind, and placebo controlled | 51 | 1 cup (150 mL) daily of low fat strawberry yogurt at breakfast; 4 weeks | 1 cup (150 mL) daily of low fat strawberry yogurt with 3.3 g of plant stanol ester at breakfast; 4 weeks | Cardiovascular disease risk factors; Weight management | Serum b-carotene and retinol were unchanged in both groups; and there were no significant changes in the normal yogurt group for all outcomes. | Adults (18-65 yr) |
| Zhang et al. (2011) ([151](#_ENREF_151)) | Cross sectional | 5427 (NR; NR) | Yogurt; NR. | Cake, milk, ice-cream, vegetables, other foods; NR. | Cardiovascular disease risk factors | The consumption of yogurt was significantly associated with elevated blood pressure in the 3 ethnic paediatric populations. | Older children (6-18 yr) |

CLA; conjugated linoleic acid; DBP, diastolic blood pressure; ELTP, enzymatically hydrolyzed lactotripeptides; GOT, glutamate-oxaloacetate transaminase; GPT, glutamate-pyruvate transaminase; NR, not reported; PS, plant sterol; PUFA, polyunsaturated fatty acids; SBP, systolic blood pressure; TAG, triacylglycerol; TG, triglyceride.

**Supplementary Table H: Summary table for yogurt studies assessing gastrointestinal health outcomes**

| **Reference** | **Study**  **design** | **Total sample size: (Intervention; Comparator)** | **Intervention details (duration)** | **Comparator details**  **(duration)** | **Eligible outcomes** | **Comments** | **Age group** |
| --- | --- | --- | --- | --- | --- | --- | --- |
| Ballesta et al. (2008) ([11](#_ENREF_11)) | Double blind cohort | NR | Fresh yogurt; 75 days. | Pasteurised yogurt; 75 days. | GI discomfort | No significant differences between groups. | Adults (18-65 yr) |
| Bhatnagar et al. (1998) ([152](#_ENREF_152)) | Randomised trial | 102 (47; 49) | Yogurt formula (67 cal/100 ml was prepared by adding 90gm of a pre-prepared standard starter (Streptococcus thermophilus and Lactobacillus bulgaricus) to formula milk (450 gm milk powder in 2700 ml of boiled and cooled water), in addition to rice lentil oil gruel and ad libitum breast feeding; 120 ml/kg of body weight/24 hr in seven divided feedings starting from the time of randomization. | Milk formula (Lactogen-2; Nestle India Ltd) providing 67 cal/100 ml of the prepared formula) in addition to rice lentil oil gruel and ad libitum breast feeding; 120 ml/kg of body weight/24 hr in seven divided feedings starting from the time of randomization. | Diarrhoea symptoms | Duration of diarrhoea, treatment failure rates and stool weights were similar between groups. Stool-reducing substances (>1%) were detected more frequently in the milk formula group. The children consuming milk had higher median percent weight gain at the end of 72 hours of the study (p = 0.04) and at recovery (p = 0.02) compared to yogurt. | Infants <1 yr and Young children 1-5 yrs |
| Boudraa et al. (1989) ([153](#_ENREF_153)) | Randomised trial | 56 (24; 30) | Yogurt; NR. | Milk; duration NR. | Diarrhoea symptoms | Clinical advantage of yogurt over milk in children with persistent diarrhoea. | Infants (<1 year) |
| Boudraa et al. (1990) ([154](#_ENREF_154)) | RCT | 45 (21;24) | Yogurt (Streptococcus thermophilius; Lactobacillus bulgaricus) (between 2-4 servings/ day dependant on age) alongside cereals and other foods; 5 days. | Milk formula (between 2-4 servings/ day dependant on age) alongside cereals and other foods; 5 days. | Diarrhoea symptoms | Strong suggestion of clinical benefit of yogurt over milk; tendency to greater weight gain despite lower energy intake; smaller liquid stool output. | Babies/Young children (3-36 months) |
| Boudraa et al. (2001) ([155](#_ENREF_155)) | Randomized controlled-trial | 112 (56;56) | Infant formula (ENAPAL-SOPAD) fermented with Lactobacillus bulgaricus and Streptococcus thermophilus. Lactose concentration (40 to 42 g/L), pH 4.5. Children received vegetables (50% wt/vol) with 12% (wt/wt) fat added in the form of butter and cereals (Sinlac, Nestlé). In partially breast-fed children, breastfeeding was not discontinued; NR. | Infant formula (ENAPAL-SOPAD). Children received vegetables (50% wt/vol) with 12% (wt/wt) fat added in the form of butter and cereals (Sinlac, Nestlé). In partially breast-fed children, breastfeeding was not discontinued; NR. | Diarrhoea symptoms | Success rate was similar in both groups (82% in milk group vs. 84% in yogurt group). Duration of diarrhoea and number of stools were significantly less in yogurt compared with milk group; only in the patients with reducing sugars in stools did yogurt significantly reduce the duration of diarrhoea and number of stools per day. | Infants <1 yr and Young children 1-5 yrs |
| Clegg et al. (2011) ([58](#_ENREF_58)) | Randomised single-blind cross over trial | 11 (11; 11; 11) | Low fat yogurt, 260g once daily, in addition to a normal diet; 3 days. | High fat yogurt (260g yogurt with 90g of sunflower oil), once daily, in addition to a normal diet; No additional yogurt; 3 days. | Weight management; Cardiovascular risk factors; GI transit time | GI transit time was significantly shorter after the high-fat yogurt compared to low fat and no yogurt, low fat yogurt had the highest GI transit time. Gastric emptying was shortest following the high fat yogurt; low fat yogurt did not alter gastric emptying. | Adults (18-65 yr) |
| Conway et al. (2007) ([156](#_ENREF_156)) | Three-arm randomised controlled trial with double blinding of the two yogurt arms. | 407 (127; 143; 137) | Commercial yogurt (Streptococcus thermophillus; Lactobacillus delbrueckii subsp. bulgaris), 150g daily; 12 days. | 150ml daily of Bio yogurt (Streptococcus thermophillus; Lactobacillus acidophilus; Bifidobacteria anamalis subsp. lactus), no yogurt; 12 days. | Diarrhoea symptoms | No significant difference between groups. | Adults (18-65 yr) |
| Dehesa et al. (1986) ([157](#_ENREF_157)) | NR | 26 (NR; NR) | Yogurt (1-2 shots/day); NR. | Milk; NR. | Diarrhoea symptoms | Unclear outcome reporting in translation. | Infants (<1yr) and Younger children (1-5 yr) |
| Eren et al. (2010) ([158](#_ENREF_158)) | Randomized, prospective but open clinical trial | 55 (27; N/A) | A fluid extracted from Pinar yogurt made by a ferment containing Lactobacillus bulgaricus and Streptococcus thermophilus, 10^7^ microorganism/100 mL, provided by manufacturer; approximately 60 mL of YF. 15 mL twice a day for children < 2 years and 30 mL twice a day for children ≥ 2 years of age; duration= treatment was continued until diarrhoea resolved. | N/A | Diarrhoea symptoms | A non-significant reduction in hospital stay with yogurt fluid. A significant reduction in daily stool frequency change with yogurt fluid was achieved at day 2. In outpatient cases, yogurt treatment was cheaper than S. boulardii (a non-relevant comparator) whereas in hospitalized patients, treatment cost was similar. | Young children (1-5) Older children (6-18) |
| Frank et al. (2012) ([159](#_ENREF_159)) | Single blinded crossover trial | 11 (11; 11) | Low-fat (0.1%) yogurt (500ml) in less than or equal to 10 min. the protein content was set to 3.4 6 0.1% (wt:wt); calorific value was approx. 40kcal/100g. Lactobacillus delbrueckii subspecies bulgaricus and Streptococcus thermophilus; One serving | GI transit time | GI transit time | No difference in hunger between low and high fat yogurt. A pronounced reduction of cerebral blood flow in the hypothalamus in response to the intake of high-fat yogurt (correlated positively with insulin change), whereas the low-fat condition showed no such effect. | Adults (18-65 yr) |
| Haenni et al. (2009) ([160](#_ENREF_160)) | Controlled, double-blind, cross-over-designed | 15 (15; 15) | Yogurt with milk fat; one serving. | Yogurt with 8.5g of milk fat replaced with stable palm and oat oil emulsion (Fabuless, previously known as Olibra); one serving. | GI transit time | A statistically significant delay in the appearance of sulfapyridine in serum and 45-min longer orocecal transit time after yogurt with stable fat emulsion versus yogurt with milk fat. | Adults (18-65 yr) |
| Isakov et al. (2013) ([161](#_ENREF_161)) | Open randomized parallel group (cohort) | 40 (NR; NR) | Standard yogurt; 2 weeks. | Yogurt enriched with inulin; 2 weeks. | Constipation symptoms | Orocecal transit time did not reduce significantly in standard yogurt. | NR-Likely to be Adults (18-65 yr) and Older adults (over 65) |
| Karabocuoglu et al. (1993) ([162](#_ENREF_162)) | Cohort | 28 (8; 10 in regular milk formula group; 10 in Low lactose formula group) | Yogurt only diet; NR. | Regular milk formula used before the onset of diarrhoea vs Low lactose formula (Nutricia Almiron 1.3 g/dl lactose); NR. | Diarrhoea symptoms | On the third day of follow up, no significant differences were found between the three groups in the number of defecations, stool pH or amounts of reducing substance in the stool. Shorter duration of diarrhoea in the group receiving yogurt as compared to the group on the regular milk formula. | Infants (<1 year) |
| Nakamura et al. (2000) ([163](#_ENREF_163)) | Cohort | 18 (18; 6; 6; 6; 18) | Yogurt, 200ml daily (S.thermophilius and L. bulgaricus) as part of a diet with no probiotics; 1 week. | Yogurt supplemented with brewers cell yeast (2g, 4g, 6g per 200ml, daily) as part of a diet with no probiotics; No Yogurt (normal diet only); 1 week. | Constipation symptoms | The group given yogurt with 4g and 6g of brewers cell yeast had increased stool frequency and faecal quantity, compared the yogurt with no supplementation group. | Adults (18-65 yr) |
| Nakamura et al. (2001) ([164](#_ENREF_164)) | Cross over trial | 24 (24; 24) | Yogurt (standard), 200ml every day; 1 week. | Yogurt with brewers cell yeast, 6g in 200ml every day; No yogurt treatment (initial assessment); 1 week. | Constipation symptoms | Stool frequency, faecal quantities, faecal water content and levels of short-chain fatty acids were significantly higher for the brewer’s yeast yogurt group compared to the standard yogurt group. Faecal pH of subjects given the brewers cell yeast yogurt diet was significantly decreased in comparison with the control subjects. Stool frequency and faecal quantities were not significantly different when comparing standard yogurt to a non-treated control group. | Younger Adults (19 - 28) |
| Niv et al. (1963) ([165](#_ENREF_165)) | Cohort | 45 (25; 20) | Yogurt (100 ml, 3 times daily), supplied by Dannon Milk Products Inc.; variable, continued until recovery. | Neomycin (1 teaspoon of Neomycin-kao-pecate, three times daily); variable, continued until recovery. | Diarrhoea recovery | Yogurt group had a more rapid recovery, significantly more recovered within 3 days, compared to the Neomycin group. | Infants (<1 year) and Young children (1-5 years) |
| Pashapour & Iou (2006) ([12](#_ENREF_12)) | Randomized controlled Trial | 80 (40; 40) | Yogurt (pasteurised cow’s milk with Lactobacillus bulgaris and Streptococcus thermophilus) 15 ml/kg/day plus routine hospital treatment up to the time of hospital discharge | Routine hospital treatment up to the time of hospital discharge. | Diarrhoea symptoms | Average number of hospitalization days was significantly lower, weight gain was higher and reduction in frequency of diarrhoea was higher in yogurt than no yogurt group. | Infants (<1 year) |
| Pilipenko et al. (2009) ([166](#_ENREF_166)) | NR | 76 (NR; NR) | Drinking yogurt; NR. | Inulin-fortified spoon yogurt; kefir drink; NR. | GI symptoms and GI quality of life | The biggest effect was marked upon use of spoon yogurt and kefir drink. After use of drinking yogurt, dyspeptic effects (impaired indigestion) were observed in a third of patients. Other effects difficult to ascertain from the abstract. | Adults (18-65 yr) and older adults (>65yr) |
| Porkka et al. (1988) ([167](#_ENREF_167)) | Controlled trial | 11 (6; 5) | Yogurt (150ml) with lactulose, daily. Produced utilising lactose-hydrolysed fat free milk and Lactobacillus acidophilus starter bacteria; 10 days. | Glucose solution (150ml with 30g glucose); 10 days. | Digestive health | Intestinal transit time was faster after lactulose yogurt than after glucose solution and did not change over 10 days. | Adults (18-65) and Older Adults (>65) |
| Rafeey et al. (2008) ([168](#_ENREF_168)) | Randomised trial | 80 (40; 40) | Conventional yogurt (300g per day, semi-fluid); from hospital admission to discharge. | Placebo; from hospital admission to discharge. | Diarrhoea symptoms | Mean frequency of stool passing on day 3 post admission and mean amount of decrement in stool passing frequency 72h after hospitalisation were significantly higher and lower in the yogurt group, respectively. Compared to the control group the conventional yogurt group had a shorter stay in hospital, were less dehydrated and had a greater change in stool frequency. | Young children (1-5) |
| Ranasinghe et al. (2007) ([169](#_ENREF_169)) | Randomised controlled trial | 76 (39; 37) | Locally manufactured yogurt (75ml) every morning with the commencement of antibiotics; 3 days. | Unclear; 3 days. | Diarrhoea occurrence | In the yogurt group 2/39 developed diarrhoea while in the control group 10/37 developed diarrhoea following antibiotic treatment, representing a significant reduction in the yogurt group. | Young children (1-5) |
| Teuri & Korpela (1998) ([170](#_ENREF_170)) | Double-blind two-period cross-over | 14 (14; 14) | Yogurt (130g, 2 servings daily); 2 weeks. | Yogurt, containing galacto-oligosaccharides (130g, 2 servings daily); 2 weeks. No yogurt run in period; 1 week. No yogurt wash out period; 2 weeks. | Constipation symptoms | The defecation frequency per week (mean, range) was higher during the galact-ooligosaccharide yogurt period than during the non-supplemented yogurt. Adverse gastrointestinal symptoms were similar during both periods. | Older adults (65+) |
| Trapp et al. (1993) ([108](#_ENREF_108)) | Cohort study, double blinded | 98 (NR; NR) | Live-active yogurt, 1.5% milk fat, 200g daily vs Heat-killed yogurt, 1.5% milk fat, 200g daily. Yogurt provided by the National Yogurt Foundation; 12 months. | No yogurt consumed (non-regular yogurt eaters told not to consume yogurt); 12 months. | Cardiovascular risk factors; GI discomfort; weight management; nutrient intake; Allergic symptoms | Trend towards less gastrointestinal distress (gas, diarrhoea, constipation) in live active yogurt consumers compared to heat killed and no yogurt. | Adults (18-65 yr) and older adults (>65yr) |
| Vazquez Martinez et al. (2005) ([171](#_ENREF_171)) | Cross sectional | NR | Pasteurised yogurt consumers; Non-pasteurized yogurt consumers; NR. | Non-consumers; NR. | GI quality of life | No significant differences between groups. | NR-Likely to be Adults (18-65 yr) |

GI, gastrointestinal; NR, not reported.

**Supplementary Table I: Summary table for yogurt studies assessing cancer outcomes**

| **Reference** | **Study**  **design** | **Total sample size: (Intervention; Comparator)** | **Intervention details (duration)** | **Comparator details**  **(duration)** | **Eligible outcomes** | **Comments** | **Age group** |
| --- | --- | --- | --- | --- | --- | --- | --- |
| Bonthuis et al. (2010) ([121](#_ENREF_121)) | Prospective cohort | 1529 (NR; NR) | Yogurt; NR. | Other various foods, identified by food frequency questionnaire; NR. | Cancer mortality; cardiovascular mortality; all-cause mortality | Yogurt was not associated with cancer mortality. | Adults (18-65 yr) and Older adults (over 65) |
| Boutron et al. (1996) ([172](#_ENREF_172)) | Case-control | 1268 (NR; NR) | Yogurt (fermented milk containing living Lactobacillus delbrueckii subsp. bulgaricus and Streptococcus salivarius subsp. thermophilus); NR. | Calcium; phosphorous; vitamin D; Milk; Low fat milk; Cheese; Cottage Cheese; NR. | Colorectal adenomas/cancer risk | Consumption of yogurt displayed an inverse relationship with risk of large adenomas, in both men and women. | Adults (18-65 yr) and Older adults (over 65) |
| Cook-Mozaffari et al. (1979) ([173](#_ENREF_173)) | Case-control | 1629 (543 cases and 1086 controls) (NR; NR) | Yogurt; NR. | Sheep's milk, sesame oil, chewing of nass, making of carpets, use of pregnancy diets, salting and sun-drying of meat and use of wild spinach. The use of opium and tea, low socio-economic status, fruit and vegetables; NR. | Oesophageal cancer risk (other cancers as a secondary outcome) | Lack of association of sheep’s yogurt with cancer. | Adults (18-65 yr) and Older adults (over 65) |
| Cramer et al. (1989) ([18](#_ENREF_18)) | Case-control | 474 (NR; NR) | Yogurt; NR. | Other dairy products including Whole milk; Skimmed milk; Ice milk; Ice Cream; Cottage cheese; Cream Cheese; NR. | Ovarian cancer risk; Cardiovascular risk factors | Eating yogurt at least once a month was associated with a risk of ovarian cancer. | Adults (18-65 yr) and Older adults (over 65) |
| Djonovic & Arsenijevic (2011) ([174](#_ENREF_174)) | Hospital based case-control | 200 (NR; NR) | Yogurt; NR. | Other various foods, including milk (identified as part of a food frequency questionnaire); NR. | Larynx cancer risk | Consuming yogurt acts protectively in terms of decreasing risk of cancer development. | NR-Likely to be Adults (18-65 yr) |
| Duarte-Salles et al. (2013) ([175](#_ENREF_175)) | Cohort and nested case-control | 477206 (NR; NR) | Yogurt; NR. | Milk, yogurt, cheese; NR. | Hepatocellular carcinoma (HCC) risk | Non-significant association between yogurt and HCC risk. A nested HCC case and matched control sub-set of the cohort was assessed for hepatitis B/C virus infections status. | Adults (18-65 yr) and Older adults (over 65) |
| Faber et al. (2012) ([176](#_ENREF_176)) | Population-based case-control | 2118 (NR; NR) | Yogurt; NR. | Other various foods, including total dairy, milk, soured milk (identified as part of a food frequency questionnaire); NR. | Ovarian cancer risk | Yogurt was significantly associated with ovarian cancer risk. | Adults (18-65 yr) and Older adults (over 65) |
| Gallus et. al. (2006) ([177](#_ENREF_177)) | Network of 7 case-control studies | NR | Yogurt (glasses/week, a glass corresponding to 125 g); NR. | Milk, cheese and dishes containing cheese; NR. | Risk of cancer (Oral cavity, pharynx, oesophagus, colorectum, larynx, breast, ovary, and prostate) | Non-significant inverse associations with yogurt consumption were found for cancers of the oral cavity and pharynx (OR=0.75), larynx (OR=0.74) and breast (OR=0.90). | Adults (18-65 yr) and Older adults (over 65) |
| Genkinger et al. (2006) ([178](#_ENREF_178)) | Pooled analysis of cohort studies | 553217 (NR; NR) | Yogurt (including low fat); NR. | Total milk vs Hard cheese vs Cottage cheese vs Ice cream; NR. | Ovarian cancer risk | No statistically significant associations were observed between intake of yogurt and dietary and total calcium intake and risk of ovarian cancer. | Adults (18-65 yr) |
| Heck et al. (2008) ([179](#_ENREF_179)) | Hospital-based multicentre case–control | 1231 (NR; NR) | Yogurt (curd) consumption; NR. | Other items, including dairy, as part of a food frequency questionnaire; NR. | Risk of hypopharyngeal cancers | Protective associations were seen at the highest quartile of curds (yogurt). Results were similar, although not as robust, among persons who had never smoked or chewed tobacco. | Adults (18-65 yr) |
| Hsu et al. (2007) ([180](#_ENREF_180)) | Hospital-based case-control | 2574 (NR; NR) | Yogurt; NR. | Various other foodstuffs (including milk, meat, vegetables, cruciferous vegetables, preserved vegetables) identified as part of an epidemiological study; NR. | Kidney cancer risk | Increased association with kidney cancer for consumption of yogurt. | Adults (18-65 yr) and Older adults (over 65) |
| Janoutova et al. (2007) ([181](#_ENREF_181)) | International case-control | 635 (NR; NR) | Yogurt; NR. | Other foods (including milk and cheese), weight and height (assessed by questionnaire); NR. | Kidney cancer risk | Inverse association of kidney cancer was found in high consumption of yogurt | Adults (18-65 yr) and Older adults (over 65) |
| Juarranz Sanz et al. (2004) ([182](#_ENREF_182)) | Case-control | 392(NR; NR) | Yogurt; NR. | Various other food stuffs (assessed by questionnaire); NR. | Colorectal carcinoma association | Yogurt showed a modest inverse association with risk of colorectal cancer | NR-Likely to be Adults (18-65 yr) and Older adults (over 65) |
| Kampman et al. (1994) ([183](#_ENREF_183)) | Case-cohort | 3346 (NR; NR) | Yogurt; 1 year. | Nine dairy items, split into: cheese; unfermented Milk; fermented milk, also analysed separately for some items; 1 year. | Colorectal cancer risk | A non-significantly decreased risk associated with the consumption of yogurt in the highest category of intake compared to lowest intake. | Adults (18-65) and Older Adults (>65) |
| Kampman et al. (2000) ([184](#_ENREF_184)) | Case-control | 4403 (NR; NR) | Yogurt consumption; NR. | Other items, including dairy, as part of a food frequency questionnaire; NR. | Colon cancer risk | Dietary calcium and total low fat dairy were associated but yogurt alone was not specifically associated with colon cancer risk. | Adults (18-65 yr) and older adults (>65 yr) |
| Karagianni et al. (2010) ([185](#_ENREF_185)) | Case control | 104 (NR; NR) | Yogurt; NR. | Lifestyle and dietary factors, including physical activity; cheese; fish; garlic; vegetables (questionnaire); NR. | Colorectal polyp risk (histologically advanced) | Yogurt was inversely associated with colorectal polyp formation. | NR-Likely to be Adults (18-65 yr) and older adults (>65yr) |
| Kawakita et al. (2012) ([186](#_ENREF_186)) | Case-control | 3836 (NR; NR) | Yogurt; NR. | Various other food stuffs (assessed by questionnaire); NR. | Upper aerodigestive tract cancer risk | Significant inverse association between yogurt intake and upper aerodigestive tract cancer risk, and significant inverse association among patients with hypopharyngeal, laryngeal, and oesophageal cancer. High yogurt intake tended to reduce odds ratios in oral cavity cancer. | Adults (18-65 yr) and older adults (>65yr) |
| Kesse et al. (2005) ([187](#_ENREF_187)) | Population based cohort | Adenoma study: 5320; Colorectal cancer study: 67484 | Yogurt consumption; NR. | Other items, including dairy, as part of a food frequency questionnaire; NR. | Colorectal adenoma and cancer risk | No specific effect of yogurt was observed on colorectal tumour risk. Increasing consumption of total dairy products decreased the relative risk of adenoma but not colorectal cancer. | Adults (18-65 yr) |
| Kesse et al. (2006) ([188](#_ENREF_188)) | Case-cohort | 2776 (NR; NR) | Yogurt; NR. | Other dairy products (including: milk, cheese, fresh cheese); NR. | Prostate cancer risk | A higher (significant) risk of prostate cancer was observed among yogurt consumers (independently of the Ca content). | Adults (18-65 yr) |
| Kocic et al. (1997) ([189](#_ENREF_189)) | Case-control | 212 (NR; NR) | Yogurt; NR. | Various other food stuffs (assessed by questionnaire); NR. | Breast cancer risk | Inverse relationship between yogurt consumption and breast cancer risk. | NR-Likely to be Adults (18-65 yr) and older adults (>65 yr) |
| Kojima et al. (2004) ([190](#_ENREF_190)) | Population based cohort | 107824 (NR; NR) | Yogurt consumption; NR. | Other items, including dairy, as part of a food frequency questionnaire; NR. | Colorectal cancer mortality | Yogurt intake was also inversely associated with male rectal cancer mortality; the risk for the high-intake group was less than one-half of the risk for the low-intake group. | Adults (18-65 yr) and older adults (>65 yr) |
| Kurahashi et al. (2008) ([10](#_ENREF_10)) | Case-cohort | 43435 (NR; NR) | Yogurt; NR. | Other foods, including dairy; NR. | Prostate cancer risk | Yogurt was significantly associated with a dose-dependent increase in the risk of prostate cancer. | Adults (18-65) and Older Adults (>65) |
| Le et al. (1986) ([191](#_ENREF_191)) | Case-control | 2960 (NR; NR) | Yogurt; NR. | Butter vs Cheese vs Milk vs Alcoholic beverages with meals; NR. | Breast cancer risk | The risk of breast cancer decreased significantly with increasing consumption of yogurt. | Adults (18-65 yr) and older adults (>65yr) |
| Lin et al. (2005) ([192](#_ENREF_192)) | Population based cohort | 36976 (NR; NR) | Yogurt consumption; NR. | Other items, including dairy, as part of a food frequency questionnaire; NR. | Colorectal cancer risk | Intake of yogurt alone was not significantly associated with colorectal cancer risk. | Adults (18-65 yr) |
| Matsumoto et al. (2007) ([193](#_ENREF_193)) | Prospective cohort | 11606 (NR; NR) | Yogurt; NR. | Milk; butter | Various cancer types (death) risk | Yogurt was not associated with any type of tumour deaths. | Adults (18-65 yr) and Older adults (over 65) |
| Mommers et al. (2006) ([194](#_ENREF_194)) | Population-based Case cohort | 2216 (NR; NR) | Yogurt; NR. | Other dairy products including milk and cheese; NR. | Ovarian cancer risk | No association was seen between consumption of yogurt and ovarian cancer risk. | Adults (18-65 yr) and Older adults (over 65) |
| Murphy et al. (2013) ([195](#_ENREF_195)) | Prospective cohort | 477122 (NR; NR) | Yogurt - natural and flavoured and additionally fermented milk in Sweden, Norway, and Denmark; NR. | Milk vs cheese vs total dairy; NR. | Association with colorectal cancer | Significant inverse associations were observed for yogurt in the categorical models (colon not rectum); although in the linear models, these associations were non-significant. Yogurt included fermented milk in Sweden, Norway and Denmark. | Adults (18-65) and Older Adults (>65) |
| Ornelas et al. (2007) ([196](#_ENREF_196)) | Population-based case-control | 464 (NR; NR) | Yogurt; NR. | Other various food (milk; cheese; fruits; vegetables; processed meats); NR. | Colorectal cancer risk factor (H. pylori seroprevalence) | Compared with those who did not consume yogurt, there was a protective effect of eating up to one serving per week of yogurt. The association was stronger among those who consumed more than one serving per week of yogurt. | Adults (18-65) |
| Pala et al. (2011) ([197](#_ENREF_197)) | Case cohort | 45,241 (NR; NR) | Yogurt; NR. | Other various foods and calcium; NR. | Colorectal cancer risk | Risk of colorectal cancer decreased significantly with increasing yogurt consumption irrespective of fat content | Adults (18-65 yr) and Older adults (over 65) |
| Park et al. (2007) ([198](#_ENREF_198)) | Cohort | 293888 (NR; NR) | Yogurt; NR. | Various other food stuffs and beverages identified through questionnaire; NR. | Prostate cancer risk | Yogurt was not associated with increased risk of prostate cancer. | Adults (18-65 yr) and older adults (>65 yr) |
| Peters et al. (1992) ([199](#_ENREF_199)) | Population based case control | 1492 (NR; NR) | Yogurt; NR. | Other various food groups and nutrients, including milk and calcium (identified as part of a food frequency questionnaire); NR. | Colon cancer risk | Yogurt was significantly protective against the risk of cancer, and remained so, after adjustment for calcium, sources of calories and non-dietary risk factors. | Adults (18-65 yr) and Older adults (over 65) |
| Radosavljevic et al. (2003) ([200](#_ENREF_200)) | Hospital based case control | 260 (NR; NR) | Yogurt; NR. | Various other food stuffs and beverages identified through questionnaire; NR. | Bladder cancer risk | Yogurt was a statistically significant protective factor for bladder cancer. | Adults (18-65 yr) and older adults (>65 yr) |
| Reyhani et al. (2012) ([201](#_ENREF_201)) | Case control | 200 (NR; NR) | Yogurt; NR. | Other various foods, including milk (identified as part of a food frequency questionnaire); NR. | Breast cancer risk | Yogurt shows a significant correlation with breast cancer risk. | Adults (18-65 yr) and Older adults (over 65) |
| Ronco et al. (2002) ([202](#_ENREF_202)) | Case control | 333 (NR; NR) | Skim yogurt; whole yogurt; total yogurt; NR. | Various other foodstuffs, including dairy products (identified by food frequency questionnaire); NR. | Breast cancer risk | Skim yogurt and total yogurt was associated with significant decreased risks of breast cancer (after controlling for age, years of urban status, education, body mass index, age at menarche, menopausal status, family history of breast cancer, number of childbirths, total energy and total fruits). | Adults (18-65 yr) and older adults (>65yr) |
| Sakauchi et al. (2004) ([203](#_ENREF_203)) | Cohort | 114517 (NR; NR) | Yogurt; NR. | Other various foods, including dairy: milk, cheese, butter; NR. | Urothelial cancer death risk | Consumption of yogurt had no associations with the risk (it was difficult to assess a relation with the risk of urothelial cancer death because these foods were not common in Japanese diets and many subjects seldom consumed them). | Adults (18-65 yr) |
| Shannon et al. (1996) ([204](#_ENREF_204)) | Case control | 838 (NR; NR) | Yogurt; NR. | Various foodstuffs identified by questionnaire; NR. | Colon cancer risk | No important association between yogurt and adenocarcinoma of the colon. | Adults (18-65 yr) and older adults (>65 yr) |
| Sorenson et al. (2004) ([41](#_ENREF_41)) | Cross- sectional | 1000 (Approximately) (NR; NR) | Yogurt; NR. | Other dairy products (as part of a questionnaire asking about intake of 15 dairy products); NR. | Association with disease (heart disease, cancer, diabetes, hypertension, osteoporosis and obesity) | No negative associations were found in this study other than yogurt in men. Lacking detail of exact outcome association with yogurt. | Older adults (>65yr) |
| van't Veer et al. (1989) ([205](#_ENREF_205)) | Case-control | 422 (90 cases and 194 controls; 43 cases and 95 controls) | Yogurt users; NR. | Yogurt non-users | Breast cancer risk | Controls consumed more yogurt than cases. | Adults (18-65 yr) |
| Vlajinac et al. (2003) ([206](#_ENREF_206)) | Case-control | 200 (NR; NR) | Yogurt consumption; NR. | Various other food stuffs; NR. | Multiple myeloma (bone marrow cancer) risk | Frequent yogurt consumption (more than 4 times per week) was significantly inversely related to multiple myeloma risk. | Adults (18-65 yr) and Older adults (over 65) |

NR, not reported.

**Supplementary Table J: Summary table for yogurt studies assessing diabetes outcomes**

| **Reference** | **Study**  **design** | **Total sample size: (Intervention; Comparator)** | **Intervention details (duration)** | **Comparator details**  **(duration)** | **Eligible outcomes** | **Comments** | **Age group** |
| --- | --- | --- | --- | --- | --- | --- | --- |
| Choi et al. (2005) ([207](#_ENREF_207)) | Cohort study | 41 254 (NR; NR) | Yogurt; NR. | Other items, including dairy, as part of a food frequency questionnaire; NR. | Diabetes risk (type 2) | Amount of yogurt intake showed an inverse trend with the risk of type 2 diabetes, but it was not statistically significant. | Adults (18-65 yr) |
| Dougkas et al. (2012) ([64](#_ENREF_64)) | Randomised crossover trial | 40 (40; 40) | Natural set biopot yogurt (Dr Oetker) as a pre-load to a meal; One serving. | Isoenergetic semi-skimmed milk (Cravendale; Arla Foods), a mild Cheddar cheese (Sainsbury’s); isovolumetric serving (compared with milk) of non-carbonated water as a pre-load to a meal; One serving. | Weight management; Diabetes risk factors | Although there was no difference in the postprandial responses of hormones, alanine and isoleucine concentrations were higher after the intake of yogurt than cheese and milk. | Adults (18-65 yr) |
| Gheller et al. (2013) ([208](#_ENREF_208)) | Randomised repeated measures crossover design | 11 (11; 11) | Greek yogurt (171 kcal, 25g available carbohydrates, 17g protein); One serving. | Mini-sandwich type cookies (174 kcal, 25g available carbohydrates, 1.3g protein); One serving. | Diabetes risk factors | The yogurt treatment resulted in lower glycaemic and higher insulin responses compared to the cookies treatment over 120 minutes. | Older children (6-18 yrs) |
| Grantham et al. (2013) ([209](#_ENREF_209)) | Population based cohort | 5582 (NR; NR) | Yogurt consumption | Other dairy items as part of a food frequency questionnaire | Diabetes risk | Consumption of total dairy was associated with a decreased incidence of diabetes, but yogurt was not a driver of this association: there was a lack of association of yogurt consumption and incidence of diabetes. | Adults (18-65 yr) |
| Kirii et al. (2009) ([210](#_ENREF_210)) | Population based cohort | 59796 (NR; NR) | Yogurt consumption; NR. | Other items, including dairy, as part of a food frequency questionnaire; NR. | Diabetes (type 2) risk | In models with adjustment of age and area only, a significant inverse relationship between risk of type 2 diabetes and yogurt in women. The effect was not significant before the adjustment for women, and was not significant before or after adjustment for men. | Adults (18-65 yr) and older adults (>65 yr) |
| Liu et al. (2006) ([211](#_ENREF_211)) | Population based cohort | 37183 (NR; NR) | Yogurt consumption; NR. | Other items, including dairy, as part of a food frequency questionnaire; NR. | Diabetes (type 2) risk | Intake of yogurt was inversely associated with risk of type 2 diabetes. | Adults (18-65 yr) |
| Margolis et al. (2011) ([77](#_ENREF_77)) | Prospective cohort | 82076 (NR; NR) | Yogurt; NR. | High fat dairy intake; low fat dairy intake; total dairy intake; NR. | Type 2 diabetes risk; body weight | Yogurt consumption was associated with a decreased risk of type 2 diabetes. | Adults (18-65 yr) and Older adults (over 65) |
| Nakamura et al. (2002) ([136](#_ENREF_136)) | Crossover trial | 89 (89; 89) | Normal yogurt (200ml daily); 4 weeks. | Yogurt supplemented with insoluble dietary fibres (200ml daily); 4 weeks. | Cardiovascular disease risk factors; Diabetes risk factors | For the Normal yogurt group, Fasting blood sugar was significantly higher than baseline for cross-over group A. For cross-over group B, fasting blood sugar was significantly higher than baseline. There were no significant differences between group A and B. | Adults (18-65 yr) |
| O'Connor et al. (2014) ([6](#_ENREF_6)) | Nested case cohort | 4749 (NR; NR) | Yogurt; NR. | Various food stuffs identified by food diaries; NR. | Diabetes (type 2) risk; weight management | Substituting yogurt for snacks was associated with 47% lower hazard of type 2 diabetes. | Adults (18-65 yr) and older adults (>65 yr) |
| Sluijs et al. (2012) ([212](#_ENREF_212)) | Case cohort | 24475 (NR; NR) | Yogurt and thick fermented milk (e.g., sour milk); NR. | Other dairy products, including: Cheese, milk (nonfermeneted milk and fermented milk such as buttermilk), milk beverages (chocolate milk), curd, cream desserts, diary creams and milk for coffee and creamers; NR. | Type 2 diabetes risk | Yogurt and thick fermented milk intake was associated with a lower hazard of diabetes in the age- and sex-adjusted model. Note: Thick fermented milk is included here, as well as yogurt. | NR-Likely to be Adults (18-65 yr) and older adults (>65 yr) |
| Soedamah-Muthu et al. (2012) ([26](#_ENREF_26)) | Cohort | 4526 (NR; NR) | Yogurt; NR. | Various food stuffs identified by a Food Frequency Questionnaire; NR. | Diabetes (type 2) risk; Coronary heart disease risk; all-cause mortality | Intakes of yogurt were not associated with incident diabetes (type 2). | Adults (18-65 yr) |
| Sorenson et al. (2004) ([41](#_ENREF_41)) | Cross- sectional | 1000 (Approximately) (NR; NR) | Yogurt; NR. | Other dairy products (as part of a questionnaire asking about intake of 15 dairy products); NR. | Association with disease (heart disease, cancer, diabetes, hypertension, osteoporosis and obesity) | No negative associations were found in this study other than yogurt in men. Lacking detail of exact outcome association with yogurt. | Older adults (>65yr) |
| Wang et al. (2013) ([113](#_ENREF_113)) | Cross- sectional | 6526 (3510; 3016) | Yogurt (with 9 frequency categories); NR. | Non yogurt consumers; NR. | Nutrient status; Cardiovascular disease risk factors; Diabetes risk factors; Weight management | In addition, yogurt consumption was associated with insulin resistance. Compared with nonconsumers, yogurt consumers appeared to have lower fasting glucose and insulin. | Adults (18-65 yr) |

NR, not reported.

**Supplementary Table K: Summary table for yogurt studies assessing other outcomes**

| **Reference** | **Study**  **design** | **Total sample size: (Intervention; Comparator)** | **Intervention details (duration)** | **Comparator details (duration)** | **Eligible outcomes** | **Comments** | **Age group** |
| --- | --- | --- | --- | --- | --- | --- | --- |
| Berkey et al. (2013) ([224](#_ENREF_224)) | Prospective Cohort | 7011 (NR; NR) | Yogurt; NR. | Total dairy vs milk vs cheese vs dairy protein vs dairy fat; NR. | Benign breast disease risk | No signiﬁcant associations of any intervention or comparator with benign breast disease risk. | Older children  (6-18) |
| Bonthuis et al. (2010) ([121](#_ENREF_121)) | Prospective cohort | 1529 (NR; NR) | Yogurt; NR. | Other various foods, identified by food frequency questionnaire; NR. | Cancer mortality; cardiovascular mortality; all-cause mortality | Yogurt was not associated with all-cause mortality. | Adults  (18-65 yr) and Older adults (over 65) |
| Campbell et al. (1999) ([54](#_ENREF_54)) | Randomized trial | 16 (9; 7) | Low-fat, vanilla or plain, nonpasteurized yogurt (Darigold, a 1:1 ratio of L. delbruekeii ssp. bulgaricus and S. salivarius ssp. Thermophiles). Serving size was two cups (454 g); approximately 3 months. | No yogurt products; approximately 3 months. | Nutrient intake; oestrogen metabolism | No significant differences were observed within or between groups. | Adults  (18-65 yr) |
| Chen et al. (2007) ([213](#_ENREF_213)) | Cohort | 130864 (NR; NR) | Yogurt; NR. | Ice cream vs milk vs cheese; NR. | Parkinson's disease risk | Among men and women combined, total consumption of dairy products was positively associated with Parkinson’s disease risk. | Older Adults (65+) |
| Crichton et al. (2010) ([225](#_ENREF_225)) | Retrospective cross-sectional | 1183 (NR; NR) | Reduced fat Yogurt; Total Yogurt | Other dairy products; NR. | General mental/psychological health | Consumption of low fat yogurt was associated with increased quality of memory recall (p=0.029) and greater social functioning (p=0.045) in men. | Adults  (18-65 yr) |
| Goldbohm et al. (2011) ([16](#_ENREF_16)) | Case-cohort | 20782 (NR; NR) | Yogurt (full fat (3.6% fat) and skim yogurt (0.1% fat)) | Milk, buttermilk, quark and dishes were dairy products were used. 3 items of cheese and 3 of butter. These were further specified by fat content (full, low or skim) | Total (all-cause) and cardiovascular mortality (Ischemic heart disease and stroke) | In men, yogurt was inversely associated with mortality. Exclusion of the first 2 y of follow-up or prevalent cases with cardiovascular disease did not change these estimates. In women, no association between yogurt and mortality was observed. | NR-Likely to be Adults (18-65 yr) and Older adults (over 65) |
| Hyland & Sodergren (1998) ([226](#_ENREF_226)) | Cross- sectional | 595 (NR; NR) | Yogurt; NR. | Various other foods; NR. | Minor health complaints | Yogurt was one of the lifestyle factors least associated with psychological and somatic complaints (namely, accidents, anxiety, colds, depression, migraines, mouth ulcers, sleeping badly, tiredness and wheeze) but not with a separate group of somatic complaints, (namely, constipation, dandruff, diarrhoea, heartburn and thrush). | Adults  (18-65 yr) |
| Kim et al. (2010) ([216](#_ENREF_216)) | Open, double-blind, placebo controlled randomized clinical | 56 (28 (18 completers); 28 (18 completers)) | Fermented milk containing probiotics (L. bulgaricus and Streptococcus thermophilus); 12 weeks. | Lactoferrin mixed in fermented milk containing probiotics (L. bulgaricus and Streptococcus thermophilus); 12 weeks. | Acne severity | In the non lactoferrin group, no alterations in skin hydration or pH; decrease in total lesion count but not significantly lower than baseline at 12 weeks; decrease in inflammatory lesion count and skin lipid count across 12 weeks; no statistically significant difference in changes of ILC, TLC, and acne grade of all subjects between the lactoferrin and placebo groups. | Adults  (18-65 yr) |
| Kyrozis et al. (2013) ([214](#_ENREF_214)) | Prospective cohort | 26173 (NR; NR) | Yogurt; NR. | Other various food and beverage groups, including dairy products, cheese and milk; NR. | Parkinson's disease risk | No positive association with Parkinson’s disease. | Adults  (18-65) and Older Adults (>65) |
| Makino et al. (2010) ([227](#_ENREF_227)) | Randomized Controlled Trial | 142 (29 (Funagata) and 43 (Arita); 28 (Funagata) and 42 (Arita)) | Yogurt (90g daily) prepared using two lactic acid bacterial strains, L. bulgaricus OLL1073R-1 and Streptococcus thermophilus OLS3059 (1073R-1 yogurt); 8 weeks in Funagata and 12 weeks in Arita. | Milk (100g daily); 8 weeks in Funagata and 12 weeks in Arita. | Immune function | Risk of catching the common cold was about 2·6 times lower in the yogurt group than in the milk group and the increase of natural killer cell activity was significantly higher in the yogurt group than in the milk group. In addition, the quality of life score for the ‘eye/nose/throat’ system after intake was significantly higher in the yogurt group than in the milk group and the improvement of the score was correlated with the promotion of natural killer cell activity. | Adults  (18-65) and Older Adults (>65) |
| Martinez-Puig et al. (2013) ([222](#_ENREF_222)) | Prospective, randomized, double-blind, placebo controlled | 37 (19; 18) | Yogurt consumed daily; 90 days. | Yogurt supplemented with 80 mg of a preparation containing hyaluronic acid (65 %) from rooster comb (MobileeTM, Bioiberica S.A., Palafolls, Spain) per day; 90 days. | Joint discomfort | Minimal, non-significant improvements or deterioration in maximum peak torque and mean power were recorded for the non-supplemented yogurt group in terms of both control and extension. | Adults  (18-65 yr) and older adults  (>65 yr) |
| Maslova et al. (2012) ([219](#_ENREF_219)) | Prospective cohort study | 61 909 (NR; NR) | Yogurt; low fat yogurt and full fat yogurt (consumption by mothers); NR. | Whole milk; semi-skimmed milk; total dairy product, total milk and total full-fat (consumption by mothers); NR. | Association with child asthma, ever asthma and child allergic rhinitis. | No associations were found for full-fat yogurt intake and ever asthma diagnosis; Children of women who ate low-fat yogurt >1 serving/d had greater odds of a medication-related ever asthma diagnosis compared with children of women reporting no intake. They were also more likely to have a registry-based ever diagnosis and report allergic rhinitis. Low-fat yogurt intake was directly related to increased risk of both child asthma and allergic rhinitis. At 18 months whole milk was inversely associated with child asthma, the reverse was true for semi-skimmed milk, whole milk appeared protective for early-life outcomes only. | Older children  (6-18yr) and Adults  (18-65 yr) |
| Miyake et al. (2010) ([220](#_ENREF_220)) | Prospective cohort | 763 (NR; NR) | Yogurt; NR. | Total dairy products; Milk; Cheese; Calcium; Vitamin D; NR. | Wheeze and eczema risk in infants aged 16–24 months | No association with yogurt. | Infants (<1 year) and Young children  (1-5 years) |
| Miyake et al. (2011) ([215](#_ENREF_215)) | Case-control | 617 (NR; NR) | Yogurt; NR. | Dairy products, including Milk; Cheese; Ice Cream; Calcium; Vitamin D; NR. | Parkinson's disease risk | Intake of yogurt was not independently related to the risk of Parkinson’s disease. | Adults  (18-65) and Older Adults (>65) |
| Miyake et al. (2012) ([221](#_ENREF_221)) | Cross- sectional | 1745 (NR; NR) | Yogurt; NR. | Total dairy products; Milk; Cheese; calcium; Vitamin D; NR. | Wheeze, asthma, eczema and rhinoconjunctivitis | Intake of yogurt, was not related to the prevalence of asthma, eczema or rhinoconjunctivitis. Yogurt intake was significantly associated with a lower prevalence of wheeze. | Older children  (6-18yr) and Adults  (18-65 yr) |
| Morina et al. (2013) ([223](#_ENREF_223)) | Meta-analysis of two randomized controlled, double blind, parallel trials | 199 (unclear reporting) (NR; NR) | Low fat dairy product; 3 months. | Low fat dairy product supplemented with Mobilee, a rooster comb extract rich in HA and other glycosaminoglycans (80 mg/d); 3 months. | Knee function | Compared to low fat dairy product, supplemented yogurt improved total work in flexion at 180degree/s; reduced synovial effusion; and perception of pain diminished on affected knee, particularly in men older than 50. | Adults  (18-65 yr) and Older adults (over 65) |
| Mossavar-Rahmani et al. (2002) ([83](#_ENREF_83)) | Cross- sectional | 3396 (2173; 1223) | Yogurt (as eaten by consumers); duration NR. | No yogurt (non-consumers of yogurt); NR. | Weight management; nutrient intake; general health | Yogurt consumers exhibited healthier behaviour including being more physically active, having a lower rate of smoking, and consuming a healthier diet. | Adults  (18-65 yr) and older adults  (>65 yr) |
| Ramezani Tehrani et al. (2013) ([228](#_ENREF_228)) | Cohort | 134 (NR; NR) | Yogurt; NR. | Milk, cheese (and nutrients); NR. | Age of menarche | There were no significant associations between yogurt and menarcheal age. | Young children  1-5) and Older children |
| Soedamah-Muthu et al. (2012) ([26](#_ENREF_26)) | Cohort | 4526 (NR; NR) | Yogurt; NR. | Various food stuffs identified by a Food Frequency Questionnaire; NR. | Diabetes (type 2) risk; Coronary heart disease risk; all-cause mortality | For yogurt, a borderline inverse association was found with all-cause mortality. | Adults  (18-65 yr) |
| Trapp et al. (1993) ([108](#_ENREF_108)) | Cohort study, double blinded | 98 (NR; NR) | Live-active yogurt, 1.5% milk fat, 200g daily vs Heat-killed yogurt, 1.5% milk fat, 200g daily. Yogurt provided by the National Yogurt Foundation; 12 months. | No yogurt consumed (non-regular yogurt eaters told not to consume yogurt); 12 months. | Cardiovascular risk factors; GI discomfort; weight management; nutrient intake; Allergic symptoms | Yogurt consumption was associated with a decrease in allergic symptoms in both the senior and young live-active yogurt groups, compared to the heat-killed and no yogurt groups. Seniors consuming yogurt experienced a significant decrease in GGT (liver enzyme associated with fat and/or alcohol metabolism). | Adults (18-65 yr) and older adults (>65yr) |
| Uenishi et al. (2004) ([217](#_ENREF_217)) | Children with atopic dermatitis, showing irregular aggravation of skin lesions. | 59 (NR; NR) | Yogurt; NR. | Various food stuffs identified by food diaries; NR. | Aggravation of skin lesions | Yogurt was identified as one of the predominant offending foods for aggravating skin lesions. | Older children  (6-18 yr) |
| Uenishi et al. (2008) ([218](#_ENREF_218)) | Challenge trial | 69 (14 challenge tests in 14 patients; 85 challenge tests, unreported number of patients) | Yogurt (identified as a candidate trigger); One serving. | Other foods (including Chocolate; Cheese) identified as a candidate trigger; One serving. | Atopic dermatitis | Yogurt was one of the predominant offending foods in Japanese children with atopic dermatitis examined in the present study. | Older children  (6-18 yr) |

GGT, gamma-glutamyl transferase; GI, gastrointestinal; HA, hyaluronic acid; ILC, inflammatory lesion count; NR, not reported; TLC, total lesion count (TLC).

**Supplementary Material: Full Search Strategies**

**Database / information source: MEDLINE(R) In-Process & Other Non-Indexed Citations and Ovid MEDLINE(R) 1946 to Present**

Interface / URL: OvidSP

Search date: 11/11/13

Retrieved records: 4681

1 yogurt/ (1356)

2 streptococcus thermophilus/ (500)

3 lactobacillus delbrueckii/ (239)

4 (yogurt$ or yoghurt$ or yoghourt$ or yaourt$ or joghurt$ or yogourt$ or yaghourt$ or yahourth$ or yoghurd$ or joghourt$ or jogourt$).ti,ab. (2417)

5 (maas or amasi$1 or dahi$1 or da-hi$1 or dohi$1 or meesti$1 or perugu$1 or thayir$1 or thayiru$1 or mosaru$1 or curd$1 or matsun$1 or matsoon$1 or matsoun$1 or matzoun$1 or madzoon$1 or madzoun$1 or matson$1 or matsoni$1 or dadiah$1 or dadih$1 or stragisto$1 or q?zana$1 or dhahi$1 or dhaunro$1 or juju dhau$1 or rahmjoghurt$ or jameed$1 or zabadi$1 or labni$1 or lebni$1 or labneh$1 or m?st chekide$1).ti,ab. (1496)

6 (streptococcus adj3 thermophilus).ti,ab. (1318)

7 s thermophilus.ti,ab. (587)

8 (lactococcus adj3 thermophilus).ti,ab. (46)

9 l thermophilus.ti,ab. (5)

10 (lactobacillus adj3 delbruecki$).ti,ab. (762)

11 l delbruecki$.ti,ab. (314)

12 bulgaricus.ti,ab. (942)

13 or/1-12 (6063)

14 (letter or editorial or comment or case reports).pt. (2826899)

15 case report.ti. (156156)

16 animals/ not (animals/ and humans/) (3966245)

17 13 not (14 or 15 or 16) (4681)

**Database / information source: Embase <1996 to 2013 Week 45>**

Interface / URL: OvidSP

Search date: 11/11/13

Retrieved records: 4613

1 yoghurt/ (2533)

2 streptococcus thermophilus/ (1448)

3 lactobacillus bulgaricus/ (228)

4 (yogurt$ or yoghurt$ or yoghourt$ or yaourt$ or joghurt$ or yogourt$ or yaghourt$ or yahourth$ or yoghurd$ or joghourt$ or jogourt$).ti,ab. (2664)

5 (maas or amasi$1 or dahi$1 or da-hi$1 or dohi$1 or meesti$1 or perugu$1 or thayir$1 or thayiru$1 or mosaru$1 or curd$1 or matsun$1 or matsoon$1 or matsoun$1 or matzoun$1 or madzoon$1 or madzoun$1 or matson$1 or matsoni$1 or dadiah$1 or dadih$1 or stragisto$1 or q?zana$1 or dhahi$1 or dhaunro$1 or juju dhau$1 or rahmjoghurt$ or jameed$1 or zabadi$1 or labni$1 or lebni$1 or labneh$1 or m?st chekide$1).ti,ab. (1418)

6 (streptococcus adj3 thermophilus).ti,ab. (1183)

7 s thermophilus.ti,ab. (517)

8 (lactococcus adj3 thermophilus).ti,ab. (46)

9 l thermophilus.ti,ab. (1)

10 (lactobacillus adj3 delbruecki$).ti,ab. (746)

11 l delbruecki$.ti,ab. (295)

12 bulgaricus.ti,ab. (786)

13 or/1-12 (6668)

14 (letter or editorial).pt. (902665)

15 case report/ or case report.ti. (1101110)

16 (animal experiment/ or animal model/ or animal tissue/ or nonhuman/) not human/ (2086644)

17 13 not (14 or 15 or 16) (4613)

**Database / information source: Cochrane Database of Systematic Reviews (CDSR)**

Interface / URL: Cochrane Library/Wiley Interscience (online - Issue 11 of 12, Nov 2013)

Search date: 11/11/13

Retrieved records: 45

ID Search

#1 MeSH descriptor: [Yogurt] this term only (199)

#2 MeSH descriptor: [Streptococcus thermophilus] this term only (30)

#3 MeSH descriptor: [Lactobacillus delbrueckii] this term only (3)

#4 (yogurt* or yoghurt* or yoghourt* or yaourt* or joghurt* or yogourt* or yaghourt* or yahourth* or yoghurd* or joghourt* or jogourt*) (403)

#5 (maas or amasi* or dahi* or da-hi* or dohi* or meesti* or perugu* or thayir* or thayiru* or mosaru* or curd* or matsun* or matsoon* or matsoun* or matzoun* or madzoon* or madzoun* or matson* or matsoni* or dadiah* or dadih* or stragisto* or q?zana* or dhahi* or dhaunro* or juju next dhau* or rahmjoghurt* or jameed* or zabadi* or labni* or lebni* or labneh* or m?st next chekide*):ti,ab,kw (61)

#6 (streptococcus near/3 thermophilus) (94)

#7 s next thermophilus (21)

#8 (lactococcus near/3 thermophilus) (1)

#9 l next thermophilus (0)

#10 (lactobacillus near/3 delbruecki*) (17)

#11 l next delbruecki* (12)

#12 bulgaricus (57)

#13 #1 or #2 or #3 or #4 or #5 or #6 or #7 or #8 or #9 or #10 or #11 or #12 (536)

CDSR subset = 45

**Database / information source: Cochrane Central Register of Controlled Trials (CENTRAL)**

Interface / URL: Cochrane Library/Wiley Interscience (online - Issue 11 of 12, Nov 2013)

Search date: 11/11/13

Retrieved records: 464

Search strategy:

ID Search

#1 MeSH descriptor: [Yogurt] this term only (199)

#2 MeSH descriptor: [Streptococcus thermophilus] this term only (30)

#3 MeSH descriptor: [Lactobacillus delbrueckii] this term only (3)

#4 (yogurt* or yoghurt* or yoghourt* or yaourt* or joghurt* or yogourt* or yaghourt* or yahourth* or yoghurd* or joghourt* or jogourt*) (403)

#5 (maas or amasi* or dahi* or da-hi* or dohi* or meesti* or perugu* or thayir* or thayiru* or mosaru* or curd* or matsun* or matsoon* or matsoun* or matzoun* or madzoon* or madzoun* or matson* or matsoni* or dadiah* or dadih* or stragisto* or q?zana* or dhahi* or dhaunro* or juju next dhau* or rahmjoghurt* or jameed* or zabadi* or labni* or lebni* or labneh* or m?st next chekide*):ti,ab,kw (61)

#6 (streptococcus near/3 thermophilus) (94)

#7 s next thermophilus (21)

#8 (lactococcus near/3 thermophilus) (1)

#9 l next thermophilus (0)

#10 (lactobacillus near/3 delbruecki*) (17)

#11 l next delbruecki* (12)

#12 bulgaricus (57)

#13 #1 or #2 or #3 or #4 or #5 or #6 or #7 or #8 or #9 or #10 or #11 or #12 (536)

CENTRAL subset = 464

**Database / information source: Database of Abstracts of Reviews of Effects (DARE)**

Interface / URL: CRD interface

Search date: 11/11/13

Retrieved records: 57

1 MeSH DESCRIPTOR Yogurt IN DARE (1)

2 MeSH DESCRIPTOR streptococcus thermophilus IN DARE (3)

3 MeSH DESCRIPTOR Lactobacillus delbrueckii IN DARE (0)

4 (yogurt* or yoghurt* or yoghourt* or yaourt* or joghurt* or yogourt* or yaghourt* or yahourth* or yoghurd* or joghourt* or jogourt*) IN DARE (17)

5 (maas or amasi* or dahi* or da-hi* or dohi* or meesti* or perugu* or thayir* or thayiru* or mosaru* or curd* or matsun* or matsoon* or matsoun* or matzoun* or madzoon* or madzoun* or matson* or matsoni* or dadiah* or dadih* or stragisto* or dhahi* or dhaunro* or juju dhau* or rahmjoghurt* or jameed* or zabadi* or labni* or lebni* or labneh* or chekide*) IN DARE (28)

6 (thermophilus) IN DARE (13)

7 (delbruecki*) IN DARE (1)

8 (bulgaricus) IN DARE (5)

9 #1 OR #2 OR #3 OR #4 OR #5 OR #6 OR #7 OR #8 (57)

**Database / information source: Database of Abstracts of Reviews of Effects (HTA)**

Interface / URL: CRD interface

Search date: 11/11/13

Retrieved records: 57

1 MeSH DESCRIPTOR Yogurt IN DARE (1)

2 MeSH DESCRIPTOR streptococcus thermophilus IN DARE (3)

3 MeSH DESCRIPTOR Lactobacillus delbrueckii IN DARE (0)

4 (yogurt* or yoghurt* or yoghourt* or yaourt* or joghurt* or yogourt* or yaghourt* or yahourth* or yoghurd* or joghourt* or jogourt*) IN DARE (17)

5 (maas or amasi* or dahi* or da-hi* or dohi* or meesti* or perugu* or thayir* or thayiru* or mosaru* or curd* or matsun* or matsoon* or matsoun* or matzoun* or madzoon* or madzoun* or matson* or matsoni* or dadiah* or dadih* or stragisto* or dhahi* or dhaunro* or juju dhau* or rahmjoghurt* or jameed* or zabadi* or labni* or lebni* or labneh* or chekide*) IN DARE (28)

6 (thermophilus) IN DARE (13)

7 (delbruecki*) IN DARE (1)

8 (bulgaricus) IN DARE (5)

9 #1 OR #2 OR #3 OR #4 OR #5 OR #6 OR #7 OR #8 (57)

**Database / information source: Health Technology Assessment (HTA)**

Interface / URL: CRD interface

Search date: 11/11/13

Retrieved records: 0

Same strategy as used in DARE – All searches produced zero records.

**Database / information source: Science Citation Index Expanded (SCI-EXPANDED)**

Interface / URL: Web of Science

Search date: 11/11/13

Retrieved records: 1556

| # 11 | 1,556 | #8 NOT (#9 OR #10) |
| --- | --- | --- |
| # 10 | 159,294 | TI=(“case study” or “case report”) |
| # 9 | 2,313,014 | TI=(animal or animals or rat or rats or mouse or mice or murine or hamster or hamsters or dog or dogs or porcine or swine or pig or pigs or cat or cats or bovine or cow or cows or ovine or sheep or equine or horse or horses or monkey$ or macaque$) |
| # 8 | 1,740 | #7 AND #4 |
| # 7 | 2,640,881 | #6 AND #5 |
| # 6 | 7,996,681 | TS=(epidemiol* or outcome$ or cohort or open-label or “clinical trial” or “clinical trials” or rct$ or random* or control* or compar* or placebo* or triple-blind* or treble-blind* or double-blind* or single-blind* or prospective) |
| # 5 | 6,432,010 | TS=(epidemiol* or medical or health or disease$ or bone$ or osteo* or weight or overweight or obes* or metabol* or heart or cardiovascular or malnutrition or malnourish* or gastrointestinal or digestive or bowel$ or diarrhea$ or diarrhoea$ or bmi or hypertens* or “blood pressure”) |
| # 4 | 13,102 | #3 OR #2 OR #1 |
| # 3 | 3,971 | TS=(maas or amasi$ or dahi$ or da-hi$ or dohi$ or meesti$ or perugu$ or thayir$ or thayiru$ or mosaru$ or curd$ or matsun$ or matsoon$ or matsoun$ or matzoun$ or madzoon$ or madzoun$ or matson$ or matsoni$ or dadiah$ or dadih$ or stragisto$ or q?zana$ or dhahi$ or dhaunro$ or “juju dhau” or “juju dhaus” or rahmjoghurt* or jameed$ or zabadi$ or labni$ or lebni$ or labneh$ or “m?st chekide$”) |
| # 2 | 4,217 | TS=(streptococcus NEAR/3 thermophilus or “s thermophilus” or lactococcus NEAR/3 thermophilus or “l thermophilus” or lactobacillus NEAR/3 delbrueckii or “l delbrueckii” or lactobacillus NEAR/3 bulgaricus or “l bulgaricus”) |
| # 1 | 6,177 | TS=(yogurt* or yoghurt* or yoghourt* or yaourt* or joghurt* or yogourt* or yaghourt* or yahourth* or yoghurd* or joghourt* or jogourt*) |

**Database / information source: Conference Proceedings Citation Index – Science (CPCI-S)**

Interface / URL: Web of Science

Search date: 11/11/13

Retrieved records: 115

| # 11 | 115 | #8 NOT (#9 OR #10) |
| --- | --- | --- |
| # 10 | 25,811 | TI=(“case study” or “case report”) |
| # 9 | 192,047 | TI=(animal or animals or rat or rats or mouse or mice or murine or hamster or hamsters or dog or dogs or porcine or swine or pig or pigs or cat or cats or bovine or cow or cows or ovine or sheep or equine or horse or horses or monkey$ or macaque$) |
| # 8 | 120 | #7 AND #4 |
| # 7 | 265,775 | #6 AND #5 |
| # 6 | 1,687,026 | TS=(epidemiol* or outcome$ or cohort or open-label or “clinical trial” or “clinical trials” or rct$ or random* or control* or compar* or placebo* or triple-blind* or treble-blind* or double-blind* or single-blind* or prospective) |
| # 5 | 78,990 | TS=(epidemiol* or medical or health or disease$ or bone$ or osteo* or weight or overweight or obes* or metabol* or heart or cardiovascular or malnutrition or malnourish* or gastrointestinal or digestive or bowel$ or diarrhea$ or diarrhoea$ or bmi or hypertens* or “blood pressure”) |
| # 4 | 1,061 | #3 OR #2 OR #1 |
| # 3 | 368 | TS=(maas or amasi$ or dahi$ or da-hi$ or dohi$ or meesti$ or perugu$ or thayir$ or thayiru$ or mosaru$ or curd$ or matsun$ or matsoon$ or matsoun$ or matzoun$ or madzoon$ or madzoun$ or matson$ or matsoni$ or dadiah$ or dadih$ or stragisto$ or q?zana$ or dhahi$ or dhaunro$ or “juju dhau” or “juju dhaus” or rahmjoghurt* or jameed$ or zabadi$ or labni$ or lebni$ or labneh$ or “m?st chekide$”) |
| # 2 | 242 | TS=(streptococcus NEAR/3 thermophilus or “s thermophilus” or lactococcus NEAR/3 thermophilus or “l thermophilus” or lactobacillus NEAR/3 delbrueckii or “l delbrueckii” or lactobacillus NEAR/3 bulgaricus or “l bulgaricus”) |
| # 1 | 530 | TS=(yogurt* or yoghurt* or yoghourt* or yaourt* or joghurt* or yogourt* or yaghourt* or yahourth* or yoghurd* or joghourt* or jogourt*) |

**Database / information source: BIOSIS Citation Index 1969 to current**

Interface / URL: Web of Science

Search date: 14/11/13

Retrieved records: 2947

| # 11 | #9 NOT #10 | 2,947 |
| --- | --- | --- |
| # 10 | TI=(“case study” or “case report”) | 83,741 |
| # 9 | #7 NOT #8 | 2,949 |
| # 8 | TI=(animal or animals or rat or rats or mouse or mice or murine or hamster or hamsters or dog or dogs or porcine or swine or pig or pigs or cat or cats or bovine or cow or cows or ovine or sheep or equine or horse or horses or monkey$ or macaque$) | 2,221,719 |
| # 7 | #4 AND #5 AND #6 | 3,274 |
| # 6 | TS=(epidemiol* or outcome$ or cohort or open-label or “clinical trial” or “clinical trials” or rct$ or random* or control* or compar* or placebo* or triple-blind* or treble-blind* or double-blind* or single-blind* or prospective) | 8,864,866 |
| # 5 | TS=(epidemiol* or medical or health or disease$ or bone$ or osteo* or weight or overweight or obes* or metabol* or heart or cardiovascular or malnutrition or malnourish* or gastrointestinal or digestive or bowel$ or diarrhea$ or diarrhoea$ or bmi or hypertens* or “blood pressure”) | 14,315,347 |
| # 4 | #3 OR #2 OR #1 | 12,914 |
| # 3 | TS=(maas or amasi$ or dahi$ or da-hi$ or dohi$ or meesti$ or perugu$ or thayir$ or thayiru$ or mosaru$ or curd$ or matsun$ or matsoon$ or matsoun$ or matzoun$ or madzoon$ or madzoun$ or matson$ or matsoni$ or dadiah$ or dadih$ or stragisto$ or q?zana$ or dhahi$ or dhaunro$ or “juju dhau” or “juju dhaus” or rahmjoghurt* or jameed$ or zabadi$ or labni$ or lebni$ or labneh$ or “m?st chekide$”) | 4,065 |
| # 2 | TS=(streptococcus NEAR/3 thermophilus or “s thermophilus” or lactococcus NEAR/3 thermophilus or “l thermophilus” or lactobacillus NEAR/3 delbrueckii or “l delbrueckii” or lactobacillus NEAR/3 bulgaricus or “l bulgaricus”) | 5,127 |
| # 1 | TS=(yogurt* or yoghurt* or yoghourt* or yaourt* or joghurt* or yogourt* or yaghourt* or yahourth* or yoghurd* or joghourt* or jogourt*) | 5,017 |

**Database / information source: African Index Medicus**

Interface / URL: http://indexmedicus.afro.who.int/

Search date: 11/11/13

Retrieved records: 4

Searched using Advanced form interface.

yogurt or yoghurt or yoghourt or yaourt or joghurt or yogourt or yaghourt or yahourth or yoghurd or joghourt or jogourt or yogurts or yoghurts or yoghourts or yaourts or joghurts or yogourts or yaghourts or yahourths or yoghurds or joghourts or jogourts or thermophilus or delbrueckii or bulgaricus [Key Word]

OR

maas or amasi or dahi or da-hi or dohi or doi or meesti or perugu or thayir or thayiru or mosaru or curd or matsun or matsoon or matsoun or matzoun or madzoon or madzoun or matson or matsoni or dadiah or dadih or stragisto or dhahi or dhaunro or juju dhau or rahmjoghurt$ or jameed or zabadi or labni or lebni or labneh or amasis or dahis or da-his or dohis or dois or meestis or perugus or thayirs or thayirus or mosarus or curds or matsuns or matsoons or matsouns or matzouns or madzoons or madzouns or matsons or matsonis or dadiahs or dadihs or stragistos or dhahis or dhaunros or rahmjoghurts or jameeds or zabadis or labnis or lebnis or labnehs [Key Word]

**Database / information source: LILACS**

Interface / URL: [http://bvsalud.org/portal/advanced/](http://bvsalud.org/portal/advanced/?lang=en)

Search date: 12/11/13

Retrieved records: 239

(tw:(“streptococcus thermophilus”)) OR (tw:(“s thermophilus”)) OR (tw:(“lactococcus thermophilus”)) OR (tw:(“l thermophilus”)) OR (tw:(“lactobacillus delbrueckii”)) OR (tw:(“l delbrueckii”)) OR (tw:(“lactobacillus bulgaricus”)) OR (tw:(“l bulgaricus”)) OR (tw:(delbrueckii and bulgaricus)) OR (tw:(yogurt or yoghurt or yoghourt or yaourt or joghurt or yogourt or yaghourt or yahourth or yoghurd or joghourt or jogourt or yogurts or yoghurts or yoghourts or yaourts or joghurts or yogourts or yaghourts or yahourths or yoghurds or joghourts or jogourts or yogur or iogurte))

(3101 records.

Filtered out records from Medline and Cochrane Library. Left with (239 records)

*The following terms were very noisy and were not searched for:*

maas or amasi or dahi or da-hi or dohi or meesti or perugu or thayir or thayiru or mosaru or curd or matsun or matsoon or matsoun or matzoun or madzoon or madzoun or matson or matsoni or dadiah or dadih or stragisto or dhahi or dhaunro or juju dhau or rahmjoghurt$ or jameed or zabadi or labni or lebni or labneh or amasis or dahis or da-his or dohis or dois or meestis or perugus or thayirs or thayirus or mosarus or curds or matsuns or matsoons or matsouns or matzouns or madzoons or madzouns or matsons or matsonis or dadiahs or dadihs or stragistos or dhahis or dhaunros or rahmjoghurts or jameeds or zabadis or labnis or lebnis or labnehs

**Database / information source: OAISTER**

Interface / URL: <http://oaister.worldcat.org>

Search date: 12/11/13

Retrieved records: 66

(“streptococcus thermophilus” OR “s thermophilus” OR “lactococcus thermophilus” OR “l thermophilus” OR “lactobacillus delbrueckii” OR “l delbrueckii” OR “lactobacillus bulgaricus” OR “l bulgaricus” OR yogurt OR yoghurt OR yoghourt OR yaourt OR joghurt OR yogourt OR yaghourt OR yahourth OR yoghurd OR joghourt OR jogourt OR yogurts OR yoghurts OR yoghourts OR yaourts OR joghurts OR yogourts OR yaghourts OR yahourths OR yoghurds OR joghourts OR jogourts) AND (epidemiol* OR medical OR health OR disease* OR bone* OR osteo* OR weight OR overweight OR obes* OR metabol* OR heart OR cardiovascular OR malnutrition OR malnourish* OR gastrointestin* OR digest* OR bowel* OR diarrhea* OR diarrhoea* OR bmi OR hypertens* OR “blood pressure” OR outcome* OR cohort OR open-label OR “clinical trial” OR “clinical trials” OR rct* OR random* OR control* OR compar* OR placebo* OR triple-blind* OR treble-blind* OR double-blind* OR single-blind* OR prospective)

(66 records)

*The following terms were very noisy and were not searched for:*

maas OR amasi OR dahi OR “da-hi” OR dohi OR meesti OR perugu OR thayir OR thayiru OR mosaru OR curd OR matsun OR matsoon OR matsoun OR matzoun OR madzoon OR madzoun OR matson OR matsoni OR dadiah OR dadih OR stragisto OR dhahi OR dhaunro OR “juju dhau” OR rahmjoghurt OR jameed OR zabadi OR labni OR lebni OR labneh OR amasis OR dahis OR “da-his” OR dohis OR dois OR meestis OR perugus OR thayirs OR thayirus OR mosarus OR curds OR matsuns OR matsoons OR matsouns OR matzouns OR madzoons OR madzouns OR matsons OR matsonis OR dadiahs OR dadihs OR stragistos OR dhahis OR dhaunros OR rahmjoghurts OR jameeds OR zabadis OR labnis OR lebnis OR labnehs

**Database / information source: OpenGrey**

Interface / URL: <http://www.opengrey.eu/>

Search date: 12/11/13

Retrieved records: 205

“streptococcus thermophilus” OR “s thermophilus” OR “lactococcus thermophilus” OR “l thermophilus” OR “lactobacillus delbrueckii” OR “l delbrueckii” OR “lactobacillus bulgaricus” OR “l bulgaricus” OR yogurt OR yoghurt OR yoghourt OR yaourt OR joghurt OR yogourt OR yaghourt OR yahourth OR yoghurd OR joghourt OR jogourt OR yogurts OR yoghurts OR yoghourts OR yaourts OR joghurts OR yogourts OR yaghourts OR yahourths OR yoghurds OR joghourts OR jogourts OR maas OR amasi OR dahi OR “da-hi” OR dohi OR meesti OR perugu OR thayir OR thayiru OR mosaru OR curd OR matsun OR matsoon OR matsoun OR matzoun OR madzoon OR madzoun OR matson OR matsoni OR dadiah OR dadih OR stragisto OR dhahi OR dhaunro OR “juju dhau” OR rahmjoghurt OR jameed OR zabadi OR labni OR lebni OR labneh OR amasis OR dahis OR “da-his” OR dohis OR dois OR meestis OR perugus OR thayirs OR thayirus OR mosarus OR curds OR matsuns OR matsoons OR matsouns OR matzouns OR madzoons OR madzouns OR matsons OR matsonis OR dadiahs OR dadihs OR stragistos OR dhahis OR dhaunros OR rahmjoghurts OR jameeds OR zabadis OR labnis OR lebnis OR labnehs

(205 records)

**Database / information source: ClinicalTrials.gov**

Interface / URL:

<http://www.clinicaltrials.gov>

Search date: 13/11/13

Retrieved records: 357 (not deduplicated)

**158 studies found for**:    “streptococcus thermophilus” OR “s thermophilus” OR “lactococcus thermophilus” OR “l thermophilus” OR “lactobacillus delbrueckii” OR “l delbrueckii” OR “lactobacillus bulgaricus” OR “l bulgaricus” OR yogurt OR yoghurt OR yoghourt OR yaourt OR joghurt 158

**138 studies found for**:    Jogourt OR yogurts OR yoghurts OR yoghourts OR yaourts OR joghurts OR yogourts OR yaghourts OR yahourths OR yoghurds OR joghourts OR jogourts OR curd OR curds

**46 studies found for**:    maas OR amasi OR dahi OR “da-hi” OR dohi OR meesti OR perugu OR thayir OR thayiru OR mosaru OR matsun OR matsoon OR matsoun OR matzoun OR madzoon OR madzoun OR matson OR matsoni OR dadiah OR dadih OR stragisto OR dhahi OR dhaunro OR “juju dhau”

**2 studies found for**:    rahmjoghurt OR jameed OR zabadi OR labni OR lebni OR labneh OR amasis OR dahis OR “da-his” OR dohis OR dois OR meestis OR perugus OR thayirs OR thayirus OR mosarus OR matsuns

**13 studies found for**:    matsoons OR matsouns OR matzouns OR madzoons OR madzouns OR matsons OR matsonis OR dadiahs OR dadihs OR stragistos OR dhahis OR dhaunros OR rahmjoghurts OR jameeds OR zabadis OR labnis OR lebnis OR labnehs

**Database / information source: International Clinical Trials Registry Platform (ICTRP)**

Interface / URL: <http://apps.who.int/trialsearch/>

Search date: 12/11/13

Retrieved records: 606

streptococcus thermophilus OR s thermophilus OR lactococcus thermophilus OR l thermophilus OR lactobacillus delbrueckii OR l delbrueckii OR lactobacillus bulgaricus OR l bulgaricus OR yogurt OR yoghurt OR yoghourt OR yaourt OR joghurt OR yogourt OR yaghourt OR yahourth OR yoghurd OR joghourt OR jogourt OR yogurts OR yoghurts OR yoghourts OR yaourts OR joghurts OR yogourts OR yaghourts OR yahourths OR yoghurds OR joghourts OR jogourts OR maas OR amasi OR dahi OR da-hi OR dohi OR meesti OR perugu OR thayir OR thayiru OR mosaru OR curd OR matsun OR matsoon OR matsoun OR matzoun OR madzoon OR madzoun OR matson OR matsoni OR dadiah OR dadih OR stragisto OR dhahi OR dhaunro OR juju dhau OR rahmjoghurt OR jameed OR zabadi OR labni OR lebni OR labneh OR amasis OR dahis OR da-his OR dohis OR dois OR meestis OR perugus OR thayirs OR thayirus OR mosarus OR curds OR matsuns OR matsoons OR matsouns OR matzouns OR madzoons OR madzouns OR matsons OR matsonis OR dadiahs OR dadihs OR stragistos OR dhahis OR dhaunros OR rahmjoghurts OR jameeds OR zabadis OR labnis OR lebnis OR labnehs

(606 records)

**Database / information source: CAB Abstracts**

Interface / URL: Proquest Dialog

Search date: 14/11/13

Retrieved records: 171

| Set#: S1 | Searched for: SU.EXACT.EXPLODE("cultured milks") | Results: 16759* |
| --- | --- | --- |
| Set#: S2 | Searched for: SU.EXACT("Streptococcus thermophilus") | Results: 3700° |
| Set#: S3 | Searched for: SU.EXACT.EXPLODE("Lactobacillus delbrueckii") | Results: 4481° |
| Set#: S4 | Searched for: TI,AB(yogurt* or yoghurt* or yoghourt* or yaourt* or joghurt* or yogourt* or yaghourt* or yahourth* or yoghurd* or joghourt* or jogourt*) | Results: 14124* |
| Set#: S5 | Searched for: TI,AB(maas OR amasi OR dahi OR "da-hi" OR dohi OR meesti OR perugu OR thayir OR thayiru OR mosaru OR curd OR matsun OR matsoon OR matsoun OR matzoun OR madzoon OR madzoun OR matson OR matsoni OR dadiah OR dadih OR stragisto OR dhahi OR dhaunro OR "juju dhau" OR rahmjoghurt OR jameed OR zabadi OR labni OR lebni OR labneh OR amasis OR dahis OR "da his" OR dohis OR dois OR meestis OR perugus OR thayirs OR thayirus OR mosarus OR curds OR curd OR matsuns OR matsoons OR matsouns OR matzouns OR madzoons OR madzouns OR matsons OR matsonis OR dadiahs OR dadihs OR stragistos OR dhahis OR dhaunros OR rahmjoghurts OR jameeds OR zabadis OR labnis OR lebnis OR labnehs) | Results: 13818* |
| Set#: S6 | Searched for: TI,AB(streptococcus N/3 thermophilus) | Results: 3871° |
| Set#: S7 | Searched for: TI,AB("s thermophilus") | Results: 1270° |
| Set#: S8 | Searched for: TI,AB(lactococcus N/3 thermophilus) | Results: 102° |
| Set#: S9 | Searched for: TI,AB("l thermophilus") | Results: 15° |
| Set#: S10 | Searched for: TI,AB(lactobacillus N/3 delbruecki*) | Results: 1835° |
| Set#: S11 | Searched for: TI,AB("l delbruecki*") | Results: 859° |
| Set#: S12 | Searched for: S1 OR S2 OR S3 OR S4 OR S5 OR S6 OR S7 OR S8 OR S9 OR S10 OR S11 | Results: 37366* |
| Set#: S13 | Searched for: TI,AB(epidemiol* or medical or health or disease* or bone* or osteo* or weight or overweight or obes* or metabol* or heart or cardiovascular or malnutrition or malnourish* or gastrointestinal or digestive or bowel* or diarrhea* or diarrhoea* or bmi or hypertens* or "blood pressure") | Results: 2224762* |
| Set#: S14 | Searched for: TI,AB(epidemiol* or outcome* or cohort or open-label or “clinical trial” or “clinical trials” or rct* or random* or control* or compar* or placebo* or triple-blind* or treble-blind* or double-blind* or single-blind* or prospective) | Results: 2978093* |
| Set#: S15 | Searched for: SU.EXACT.EXPLODE("clinical trials") | Results: 26531* |
| Set#: S16 | Searched for: SU.EXACT.EXPLODE("bone diseases") | Results: 27263* |
| Set#: S17 | Searched for: SU.EXACT("obesity") | Results: 63401* |
| Set#: S18 | Searched for: SU.EXACT.EXPLODE("weight") | Results: 134187* |
| Set#: S19 | Searched for: SU.EXACT.EXPLODE("cardiovascular diseases") OR SU.EXACT.EXPLODE("cardiovascular disorders") | Results: 83401* |
| Set#: S20 | Searched for: SU.EXACT("malnutrition") OR SU.EXACT("nutritional disorders") | Results: 19938* |
| Set#: S21 | Searched for: SU.EXACT.EXPLODE("gastrointestinal disorders") OR SU.EXACT.EXPLODE("gastrointestinal diseases") | Results: 62792* |
| Set#: S22 | Searched for: (S15 OR S14) AND (S13 OR S16 OR S17 OR S18 OR S19 OR S20 OR S21) | Results: 1019537* |
| Set#: S23 | Searched for: S22 AND S12 | Results: 2447° |
| Set#: S24 | Searched for: SU.EXACT.EXPLODE("animals") NOT SU.EXACT("man") | Results: 3039560* |
| Set#: S25 | Searched for: S23 NOT S24 | Results: 1717° |

* Duplicates are removed from your search, but included in your result count.

° Duplicates are removed from your search and from your result count.

**Database / information source: National Technical Information Service (NTIS)**

Interface / URL: Proquest Dialog

Search date: 14/11/13

Retrieved records: 143

| Set#: S1 | Searched for: su.Exact("yogurt") | Results: 13° |
| --- | --- | --- |
| Set#: S2 | Searched for: TI,AB(yogurt* or yoghurt* or yoghourt* or yaourt* or joghurt* or yogourt* or yaghourt* or yahourth* or yoghurd* or joghourt* or jogourt*) | Results: 43° |
| Set#: S3 | Searched for: TI,AB(maas OR amasi OR dahi OR "da-hi" OR dohi OR meesti OR perugu OR thayir OR thayiru OR mosaru OR curd OR matsun OR matsoon OR matsoun OR matzoun OR madzoon OR madzoun OR matson OR matsoni OR dadiah OR dadih OR stragisto OR dhahi OR dhaunro OR "juju dhau" OR rahmjoghurt OR jameed OR zabadi OR labni OR lebni OR labneh OR amasis OR dahis OR "da his" OR dohis OR dois OR meestis OR perugus OR thayirs OR thayirus OR mosarus OR curds OR curd OR matsuns OR matsoons OR matsouns OR matzouns OR madzoons OR madzouns OR matsons OR matsonis OR dadiahs OR dadihs OR stragistos OR dhahis OR dhaunros OR rahmjoghurts OR jameeds OR zabadis OR labnis OR lebnis OR labnehs) | Results: 93° |
| Set#: S4 | Searched for: TI,AB(streptococcus N/3 thermophilus) | Results: 4° |
| Set#: S5 | Searched for: TI,AB("s thermophilus") | Results: 2° |
| Set#: S6 | Searched for: TI,AB(lactococcus N/3 thermophilus) | Results: 0° |
| Set#: S7 | Searched for: TI,AB("l thermophilus") | Results: 0° |
| Set#: S8 | Searched for: TI,AB(lactobacillus N/3 delbruecki*) | Results: 2° |
| Set#: S9 | Searched for: TI,AB("l delbruecki*") | Results: 0° |
| Set#: S10 | Searched for: S1 OR S2 OR S3 OR S4 OR S5 or S6 OR S7 OR S8 OR S9 | Results: 143° |

° Duplicates are removed from your search and from your result count.

**Database / information source: FSTA®**

Interface / URL: Proquest Dialog

Search date: 18/11/13

Retrieved records: 755

| Set#: S1 | Searched for: SU.EXACT.EXPLODE("YOGHURT") | Results: 5533* |
| --- | --- | --- |
| Set#: S2 | Searched for: SU.EXACT("STREPTOCOCCUS THERMOPHILUS") | Results: 769° |
| Set#: S3 | Searched for: SU.EXACT("LACTOBACILLUS DELBRUECKII") | Results: 409° |
| Set#: S4 | Searched for: TI,AB(yogurt* or yoghurt* or yoghourt* or yaourt* or joghurt* or yogourt* or yaghourt* or yahourth* or yoghurd* or joghourt* or jogourt*) | Results: 10202* |
| Set#: S5 | Searched for: TI,AB(maas OR amasi OR dahi OR "da-hi" OR dohi OR meesti OR perugu OR thayir OR thayiru OR mosaru OR curd OR matsun OR matsoon OR matsoun OR matzoun OR madzoon OR madzoun OR matson OR matsoni OR dadiah OR dadih OR stragisto OR dhahi OR dhaunro OR "juju dhau" OR rahmjoghurt OR jameed OR zabadi OR labni OR lebni OR labneh OR amasis OR dahis OR "da his" OR dohis OR dois OR meestis OR perugus OR thayirs OR thayirus OR mosarus OR curds OR curd OR matsuns OR matsoons OR matsouns OR matzouns OR madzoons OR madzouns OR matsons OR matsonis OR dadiahs OR dadihs OR stragistos OR dhahis OR dhaunros OR rahmjoghurts OR jameeds OR zabadis OR labnis OR lebnis OR labnehs) | Results: 7623* |
| Set#: S6 | Searched for: TI,AB(streptococcus N/3 thermophilus) | Results: 2825° |
| Set#: S7 | Searched for: TI,AB("s thermophilus") | Results: 741° |
| Set#: S8 | Searched for: TI,AB(lactococcus N/3 thermophilus) | Results: 66° |
| Set#: S9 | Searched for: TI,AB("l thermophilus") | Results: 4° |
| Set#: S10 | Searched for: TI,AB(lactobacillus N/3 delbruecki*) | Results: 1221° |
| Set#: S11 | Searched for: TI,AB("l delbruecki*") | Results: 448° |
| Set#: S13 | Searched for: S1 OR S2 OR S3 OR S4 OR S5 or S6 OR S7 OR S8 OR S9 OR S10 OR S11 | Results: 19822* |
| Set#: S14 | Searched for: TI,AB(epidemiol* or medical or health or disease* or bone* or osteo* or weight or overweight or obes* or metabol* or heart or cardiovascular or malnutrition or malnourish* or gastrointestinal or digestive or bowel* or diarrhea* or diarrhoea* or bmi or hypertens* or "blood pressure") | Results: 164050* |
| Set#: S15 | Searched for: TI,AB(epidemiol* or outcome* or cohort or open-label or "clinical trial" or "clinical trials" or rct* or random* or control* or compar* or placebo* or triple-blind* or treble-blind* or double-blind* or single-blind* or prospective) | Results: 272247* |
| Set#: S16 | Searched for: SU.EXACT.EXPLODE("CARDIOVASCULAR DISEASES") OR SU.EXACT("BONE HEALTH") OR SU.EXACT("OSTEOPOROSIS") OR SU.EXACT("OBESITY") OR SU.EXACT("BODY WT.") OR SU.EXACT("OVERWEIGHT") OR SU.EXACT("MALNUTRITION") OR SU.EXACT("DIARRHEA") OR SU.EXACT("INFLAMMATORY BOWEL DISEASE") OR SU.EXACT("CONSTIPATION") OR SU.EXACT("DIGESTION") | Results: 12482* |
| Set#: S17 | Searched for: S15 AND (S16 OR S14) | Results: 67454* |
| Set#: S18 | Searched for: S13 AND S17 | Results: 755° |

* Duplicates are removed from your search, but included in your result count.

° Duplicates are removed from your search and from your result count.

**Database / information source: Conferences of various organisations**

Interface / URL: [as](http://apps.who.int/trialsearch/) specified below

Search date: 13/11/13

Retrieved records: xxx

Search strategy (unless otherwise specified):

Searched text for any of:

- thermophilus
- lactococcus
- lactobacillus
- delbrueckii
- bulgaricus
- yogurt
- yoghurt
- yoghourt
- curd

***ESPEN congress*** (<http://www.espen.org>)

Leipzig, August 2013: programme only

Barcelona, September 2012: abstracts online

Gothenburg, September 2011: abstracts online

Nice, September 2010: abstracts online

(1 potentially relevant record from 2012, 1 potentially relevant record from 2011,)

***International Scientific Conference on Nutraceuticals and Functional Foods*** (International Society for Nutraceuticals and Functional Foods): <http://isnff.org>

Taipei, November 2013: programme only

Hawaii, December 2012: poster and oral presentation programmes only

Sapporo, November 2011: poster and oral presentation programmes only

Bali, October 2010: poster and oral presentation programmes only

San Francisco, November 2009: poster and oral presentation programmes only

(1 record from 2012, 3 from 2011)

***European Nutrition Conference (FENS***, <http://www.fensnutrition.eu/>)

Madrid, October 2011

[Held every four years, no conferences in 2010, 2012, 2013]

(1 record from 2011)

***Federation of American Societies for Experimental Biology (FASEB)***

FASEB Journal meeting abstracts: <http://www.fasebj.org/search>

Search strategy:

(thermophilus or delbrueckii or bulgaricus or yogurt* or yoghurt* or yoghourt* or curd*) in titles and abstracts (112 records, downloaded)

maas* or amasi* or dahi* or dohi* or meesti* or perugu* or thayir* or thayiru* or mosaru* or matsun* or matsoon* or matsoun* or matzoun* or madzoon* or madzoun* or matson* or matsoni* or dadiah* or dadih* or stragisto* or dhahi* or dhaunro* or rahmjoghurt* or jameed* or zabadi* or labni* or lebni* or labneh* (14 records, all irrelevant)

***Academy of Nutrition and Dietetics (formerly American Dietetic Association)***

*Food & Nutrition Conference and Expo (FNCE)* <http://www.eatright.org/fnce/>

October 2013, Houston

October 2012, Philadelphia

September 2011, San Diego

November 2010, Boston,

***2013*** *Food & Nutrition Conference & Expo Houston Texas*

19 October 2012 - 22 October 2013.

[Journal of the Academy of Nutrition and Dietetics](http://www.adajournal.org/search/results).: Vol 113, No 9, Sept 2013 pA1-A120 Supplement. <http://www.sciencedirect.com/science/journal/22122672/113/9/supp/S>.

Abstracts manually scanned. 0 relevant results

***2012*** *Food & Nutrition Conference & Expo Pennsylvania Convention Center*

06 October 2012 - 09 October 2012.

[Journal of the Academy of Nutrition and Dietetics](http://www.adajournal.org/search/results).: Vol 112, No 9, Sep 2012 pA1-A120 Supplement.

<http://www.adajournal.org/supplements>.

Abstracts manually scanned. 0 relevant results

***2011*** *ADA Food & Nutrition Conference & Expo*

Journal of the American Dietetic Association: Volume 111, Issue 9, Supplement, Pages A1-A128 (September 2011)

<http://www.sciencedirect.com/science/journal/00028223/111/9/supp/S>
0 relevant results

***2010*** *ADA Food & Nutrition Conference & Expo*

Journal of the American Dietetic Association: Vol 110 (9) September A1-A144 (Supplement). <http://www.sciencedirect.com/science/journal/00028223/110/9/supp/S>

0 relevant results

***Federation of European Microbiological Societies (FEMS)***

<http://www.fems-microbiology.org/congress/>

***2013*** *5^th^ Congress Leipzig, Germany*

July 21-25 <http://www2.kenes.com/FEMS2013/SCIENTIFIC/Pages/Programa_Abstracts.aspx>

***2012****: No conference held*

***2011****: 5^th^ Congress July 21-25 Geneva, Switzerland* <http://www2.kenes.com/fems2011/sci/Documents/ScientificProgram.pdf>.

***2010****: No conference held.*

0 potentially relevant records

***American Society for Microbiology (ASM):*** <http://conferences.asm.org/>

Potentially relevant conferences:

San Antonio, October 2012: abstracts online

Puerto Rico, March 2011: abstracts online

Miami, October 2010: abstracts online

0 potentially relevant records.

***American College of Gastroenterology (ACG)*** Annual Scientific Meetings: <http://gi.org/>

San Diego, 2013

Las Vegas, 2012

Washington DC, 2011

San Antonio, 2010

Abstracts published in *American Journal of Gastroenterology*

***2013*** Volume 108, Issue S1 (October 2013) Abstracts submitted for the 77th Annual Scientific Meeting of the American College of Gastroenterology <http://www.nature.com/ajg/journal/v108/n1s/index.html>

***2012***: [Oct 2012; 107 (Supplement 1s) Abstracts submitted for the 77th Annual Scientific Meeting of the American College of Gastroenterology: S1 - S880](http://www.nature.com/ajg/journal/v107/n1s/index.html). (<http://www.nature.com/search/adv_search?sp-a=sp1001702d&sp-x-1=ujournal&sp-q-1=ajg,ajgsup>)

***2011:*** [Oct 2011; 106 (Supplement 2s) Abstracts Submitted for the 76: S1 - S631](http://www.nature.com/ajg/journal/v106/n2s/index.html) (http://www.nature.com/search/adv_search?sp-a=sp1001702d&sp-x-1=ujournal&sp-q-1=ajg,ajgsup).

***2010:*** October 2010; Volume 105, Issue S1 (October 2010) (http://www.nature.com/search/adv_search?sp-a=sp1001702d&sp-x-1=ujournal&sp-q-1=ajg,ajgsup).

0 potentially relevant records

***Digestive Disease Week*** (<http://www.ddw.org/>)

Abstracts published in spring supplements to journal *Gastroenterology* (<http://www.gastrojournal.org>). Searchable on journal website.

Searches for the terms listed below were run against the relevant issues for 2013, 2012, 2011, 2010.

thermophilus OR delbrueckii OR bulgaricus OR yogurt* OR yoghurt* OR yoghourt* OR curd*

Some abstracts referring to probiotics and animals were excluded.

1 potentially relevant record downloaded from 2012, one from 2013.

***British Society of Gastroenterology*** meetings

(<http://www.bsg.org.uk/education/meeting/index.html>)

2013 BSG Glasgow, 2013: programme online

2012 DDF Liverpool, 2012: programme online

2011 BSG Birmingham, 2011: programme online

2010 BSG Liverpool, 2010: programme online

0 potentially relevant records.

***United European Gastroenterology Week***

***2013*** available as a journal supplement United European Gastroenterology Journal 2013 1: A1 <http://ueg.sagepub.com/content/1/1_suppl/A1.full>

***2012, 2011, 2010*** – titles only available at <http://www.e-learning.ueg.eu/>. Also included in Gut journal which is indexed by MEDLINE.

1 potentially relevant record.

***American Dairy Products Institute* (ADPI)**

Conference proceedings not freely available online – cannot find them indexed in any journal, have emailed to query. Response: “No we don't publish our Annual Conference proceedings in a journal.”
The Annual Conference proceedings are available on the ADPI website for 90 days after the conference.

***International Dairy Federation* (IDF)**

Not searchable – browsed.

***2013*** *World Dairy Summit* – YokoHama, Japan. Programme for Nutrition and Health aspect of the conference available <http://www.wds2013.com/eng/p_conference7.html>

***2012*** *World Dairy Summit* – Cape Town, South Africa. Programme for Nutrition and Health aspect of the conference available <http://wds2012.com/ScientificProgramme/DetailedProgrammes/Conf07.htm>

***2011*** *World Dairy Summit* – Parma, Italy. Programme for Nutrition and Health aspect of the conference available

<http://www.wds2011.com/Public/ProgramDetailPage.php?ID=1457>

***2010*** *World Dairy Summit* - New Zealand – website no longer available and doesn’t appear to be indexed.

5 potentially relevant records identified.

***International Functional Food Conference***

***2013*** conference held in August 2013 Malaysia – can’t find abstracts online or indexed. No trace of earlier meetings. No contact information.

***American Society for Nutrition (ASN)***

Abstracts published in the FASEB Journal – already searched above.

***2013:*** ASN Scientific Sessions & Annual Meeting April 2013 Boston

***2012:*** ASN Scientific Sessions & Annual Meeting April 2012 San Diego

***2011:*** ASN Scientific Sessions & Annual Meeting April 2011 Washington DC

***2010:*** ASN Scientific Sessions & Annual Meeting April 2010 Anaheim, CA

**European Society for Paediatric Gastroenterology Hepatology and Nutrition (ESPGHAN)**

***2013*** The 46^th^ Annual Meeting of The European Society of Paediatric Gastroenterology,

Hepatology and Nutrition 8 - 11 May 2013 London, United Kingdom. Abstracts available at

<http://www.espghan2013.org/docs/conference-files/46th-annual-meeting-of-espghan-abstracts>

***2012*** not available

***2011*** European Society for Paediatric Gastroenterology, Hepatology, and Nutrition Annual Meeting May 25–28, 2011 Sorrento, Italy

<http://journals.lww.com/jpgn/Documents/may2011.pdf>

***2010*** European Society for Paediatric Gastroenterology, Hepatology, and Nutrition Annual Meeting June 9–12, 2010 Istanbul, Turkey

<http://journals.lww.com/jpgn/Documents/ESPGHAN2010%20mtg%20abstr.pdf>

0 potentially relevant records

**North American Society for Pediatric Gastroenterology, Hepatology, and Nutrition (NASPGHAN)**

***2013*** North American Society for Pediatric Gastroenterology, Hepatology and Nutrition Annual Meeting October 10 – 12, 2013 Chicago, IL <http://journals.lww.com/jpgn/Documents/NASPGHAN2013_Abstract_Book%20-%20revised%20Sept%2018,%202013.pdf>

***2012*** NASPGHAN annual meeting October 18 – 20, 2012 Salt Lake City, UT

<http://journals.lww.com/jpgn/Documents/Revised%20-%20NASPGHAN2012_Abstract_BookJPGN.pdf>

***2011*** North American Society for Pediatric Gastroenterology, Hepatology, and Nutrition Annual Meeting October 20–23, 2011 Orlando, FL
 <http://journals.lww.com/jpgn/Documents/2011%20NASPGHAN%20abstracts_JPGN.pdf>

***2010*** North American Society for Pediatric Gastroenterology, Hepatology, and Nutrition Annual Meeting October 21–23, 2010 New Orleans, Louisiana

<http://journals.lww.com/jpgn/Documents/MPG201667_abs.pdf>

0 potentially relevant records

**International Scientific Association for Probiotics and Prebiotics ISAPP**

***2013*** 11^th^ Meeting of The International Scientific Association for Probiotics and Prebiotics

In Collaboration with The Sackler Institute for Nutrition Sciences and The New York Academy of Sciences (The Academy) 7 World Trade Center, 250 Greenwich St, 40^th^ Fl, New York, NY June 12‐14, 2013

<http://www.isapp.net/docs/2013_meeting_materials/2013_ISAPP_program_5June2013.pdf>

***2012*** 10th Meeting of the International Scientific Association for Probiotics and Prebiotics River Lee Hotel and University College Cork Cork, Ireland October 1-3, 2012

<http://www.isapp.net/docs/2012_program.pdf?v2>

***2011*** 9th Meeting of the International Scientific Association for Probiotics and Prebiotics

Berkeley, California October 23-25, 2011
<http://www.isapp.net/docs/2011_ISAPP_Meeting_Report.pdf>

***2010*** 8th Meeting of the International Scientific Association for Probiotics and Prebiotics

August 28-30, 2010 Gran Hotel Rey Don Jaime, Castelldefels, Spain
<http://www.isapp.net/docs/2010_ISAPP_Meeting_Report.pdf>

0 potentially relevant records
